# Taming Super-Reduced Bi_2_^3–^ Radicals with Rare Earth Cations

**DOI:** 10.1021/jacs.3c01058

**Published:** 2023-04-12

**Authors:** Peng Zhang, Rizwan Nabi, Jakob K. Staab, Nicholas F. Chilton, Selvan Demir

**Affiliations:** † Department of Chemistry, Michigan State University, 578 South Shaw Lane, East Lansing, Michigan 48824, United States; ‡ Department of Chemistry, The University of Manchester, Oxford Road, Manchester M13 9PL, U.K.

## Abstract

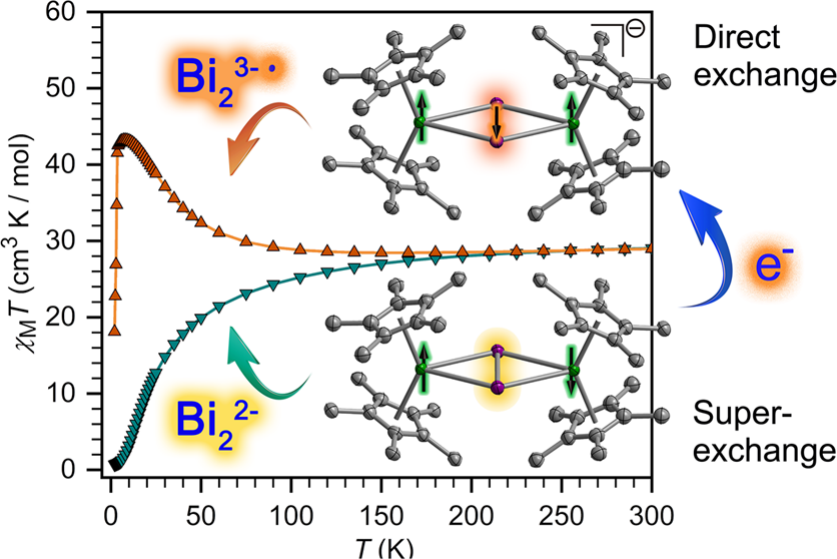

Here, we report the synthesis of two new sets of dibismuth-bridged rare earth molecules. The first series contains a bridging diamagnetic Bi_2_^2–^ anion, (Cp*_2_RE)_2_(μ-η^2^:η^2^-Bi_2_), **1-RE** (where Cp* = pentamethylcyclopentadienyl; RE = Gd (**1-Gd**), Tb (**1-Tb**), Dy (**1-Dy**), Y (**1-Y**)), while the second series comprises the first Bi_2_^3–^ radical-containing complexes for any d- or f-block metal ions, [K(crypt-222)][(Cp*_2_RE)_2_(μ-η^2^:η^2^-Bi_2_^•^)]·2THF (**2-RE**, RE = Gd (**2-Gd**), Tb (**2-Tb**), Dy (**2-Dy**), Y (**2-Y**); crypt-222 = 2.2.2-cryptand), which were obtained from one-electron reduction of **1-RE** with KC_8_. The Bi_2_^3–^ radical-bridged terbium and dysprosium congeners, **2-Tb** and **2-Dy**, are single-molecule magnets with magnetic hysteresis. We investigate the nature of the unprecedented lanthanide–bismuth and bismuth–bismuth bonding and their roles in magnetic communication between paramagnetic metal centers, through single-crystal X-ray diffraction, ultraviolet–visible/near-infrared (UV–vis/NIR) spectroscopy, SQUID magnetometry, DFT and multiconfigurational ab initio calculations. We find a π_z_^*^ ground SOMO for Bi_2_^3–^, which has isotropic spin–spin exchange coupling with neighboring metal ions of ca. −20 cm^–1^; however, the exchange coupling is strongly augmented by orbitally dependent terms in the anisotropic cases of **2-Tb** and **2-Dy**. As the first examples of p-block radicals beneath the second row bridging any metal ions, these studies have important ramifications for single-molecule magnetism, main group element, rare earth metal, and coordination chemistry at large.

## Introduction

Radical chemistry figures prominently in synthetic chemistry, life, and material sciences.^1^ In fact, radicals appear in a variety of chemical processes, especially relevant for catalysis, electrochemistry, photochemistry, and biochemistry.^2^ Despite their importance, the generation and isolation of long-lived radicals is challenging owing to the high reactivity arising from the presence of an unpaired electron located in a valence or frontier molecular orbital.^3−5^ The most prevalent synthetic approaches for the isolation of radicals typically employ the introduction of bulky substituents to impose kinetic stabilization or delocalization of unpaired electron density into conjugated systems to stabilize the singly occupied molecular orbital (SOMO).^6,7^ These strategies have led to significant advances in the generation of long-lived organic radicals and those containing heavy main group elements.^8^ In particular, diatomic radicals are highly reactive and thus remain extremely rare, except for the textbook examples NO and O_2_^–^, which possess biological and industrial relevance.^9^ Reduced species of diatomic group 15 elements are of fundamental importance, foremost regarding the activation and conversion of dinitrogen to nitride (ammonia) via various paramagnetic and diamagnetic polyanions as reactive intermediates (i.e., N_2_^–^,^10^ N_2_^2–^,^11^ N_2_^3–^,^12^). A successful synthetic strategy to tame the super-reduced N_2_^3–^ radical employs coordination of suitable electropositive metal ions, of which rare earth metals are highly efficient.^13^ Coordination of the latter dinitrogen radical with rare earth cations is not only valuable on its own right but can also engender interesting magnetic properties owing to its diffuse spin orbitals, which can penetrate the core electron density of the deeply buried 4f orbitals of the lanthanide ions.^12,14−16^

Notably, the combination of a N_2_^3–^ radical with two magnetically anisotropic ions (e.g., Dy^III^ or Tb^III^) gives rise to single-molecule magnets (SMMs) that display permanent magnet-like behavior at low temperatures (often quantified as below the blocking temperature *T*_B_, though this is somewhat a misnomer^17^) with substantial coercivities (*H*_c_) and remanent magnetization (*M*_r_).^16,18,19^ It should be noted that direct magnetic coupling between anisotropic lanthanide ions in Cp^iPr5^LnI_3_LnCp^iPr5^ via a 5d*_z_*^2^-5d*_z_*^2^ half σ bond is the only example with magnetic properties that surpasses the *H*_c_ and *M*_r_ metrics of radical-bridged multinuclear SMMs.^20^ Direct magnetic interactions notwithstanding, the lanthanide-radical approach is appealing as it is simpler to envisage more diverse coupling topologies, and yet it is still largely underexplored; this is mainly due to the challenging synthesis, isolation, and purification of highly reactive radical compounds.^21−25^ In particular, other than N_2_^3–^, no other diatomic radicals bridging magnetically anisotropic lanthanide ions are known. Even the isolation of bare, highly charged, diatomic radicals of the heavier p-block elements is extremely challenging by virtue of their high reactivity, and the pursuit of radical chemistry adds an additional layer of difficulty.^26^ When traversing from top to bottom within a group, the radicals of heavier elements are anticipated to exhibit larger covalent radii that could potentially bond to lanthanide ions with more covalent character, and thus potentially exhibit stronger magnetic exchange coupling. This in turn may improve upon the state of the art in radical-bridged SMMs, and yet, such chemistry has thus far been elusive.

A particularly intriguing candidate for generating a radical ligand is the heaviest nitrogen homolog bismuth that should engender strong coupling due to its 6s and 6p valence orbitals that have much larger radial extents compared to the 2s and 2p valence orbitals for the existing nitrogen bridges, alongside significant relativistic effects that could enhance magnetic anisotropy.^27,28^ When it comes to lanthanide chemistry, however, bismuth is a poor donor ligand.^29^ A viable synthetic path to render bismuth more accessible in coordination chemistry involves dibismuthane ligands that are formed through reductive coupling of BiR_3_, BiRX_2_, or BiCl_3_, where R = phenyl or 2,6-dimesitylphenyl,^30−32^ and Zintl anionic ligands.^33,34^ Such ligands coordinate relatively strongly to metals through their p-orbital valence electrons and give rise to complexes with d-block metals such as Mn, Fe, Co, Mo, W, and Zr.^34,35^ While dibismuth serving as a π-donor ligand has been described in the realm of transition metals,^33^ only one example with a rare earth metal is currently known,^30^ but none of them contain a dibismuth radical. The only other rare earth metal complex in which bismuth binds directly to the metal ion is the recently isolated lanthanide–bismuth heterometallocubane cluster from our group containing an unprecedented Bi_6_^6–^ Zintl ion.^36^ This highly charged closed-shell anion promotes ferromagnetic superexchange between the lanthanide ions, giving rise to SMM behavior. This report on the magnetic properties of bismuth-containing complexes shows the potential of using heavy p-block elements in molecular magnets, even if the bismuth bridge is diamagnetic. Notably, only one dibismuth radical has been crystallographically characterized, namely, in an end-on coordination toward gallium ions.^37^

Here, we report the synthesis of two new sets of bismuth-bridged molecules: the first series contains a bridging diamagnetic Bi_2_^2–^ anion between the late lanthanides gadolinium, terbium, dysprosium, and the rare earth yttrium, (Cp*_2_RE)_2_(μ-η^2^:η^2^-Bi_2_),**1-RE** (where Cp* = pentamethylcyclopentadienyl; RE = Gd (**1-Gd**), Tb (**1-Tb**), Dy (**1-Dy**), Y (**1-Y**)), and the second series comprises the first Bi_2_^3–^ radical-containing complexes for any d- or f-block metal ions, [K(crypt-222)][(Cp*_2_RE)_2_(μ-η^2^:η^2^-Bi_2_^•^)]·2THF (**2-RE**, RE = Gd (**2-Gd**), Tb (**2-Tb**), Dy (**2-Dy**), Y (**2-Y**); crypt-222 = 2.2.2-cryptand), which were obtained from one-electron reduction of **1-RE** with KC_8_. The Bi_2_^3–^ radical-bridged terbium and dysprosium congeners, **2-Tb** and **2-Dy**, are SMMs with significant magnetic hysteresis. Both sets of compounds, **1-RE** and **2-RE**, provide a valuable opportunity to probe the nature of lanthanide–bismuth and bismuth–bismuth bonding as a function of the oxidation state of the two bismuth ions. Importantly, an invaluable comparison of magnetic exchange mediated via a diamagnetic and paramagnetic side-on coordinate Bi_2_-bridge to the same metal ions with comparable geometries, and the consequences thereof, can be drawn. Equally relevant is the possibility to compare our discoveries to the properties of lighter main group radical bridges in terms of the frontier orbital compositions, covalency, magnetism, and spectroscopic transitions.

## Results and Discussion

### Synthesis and Structure

**Figure 1:**
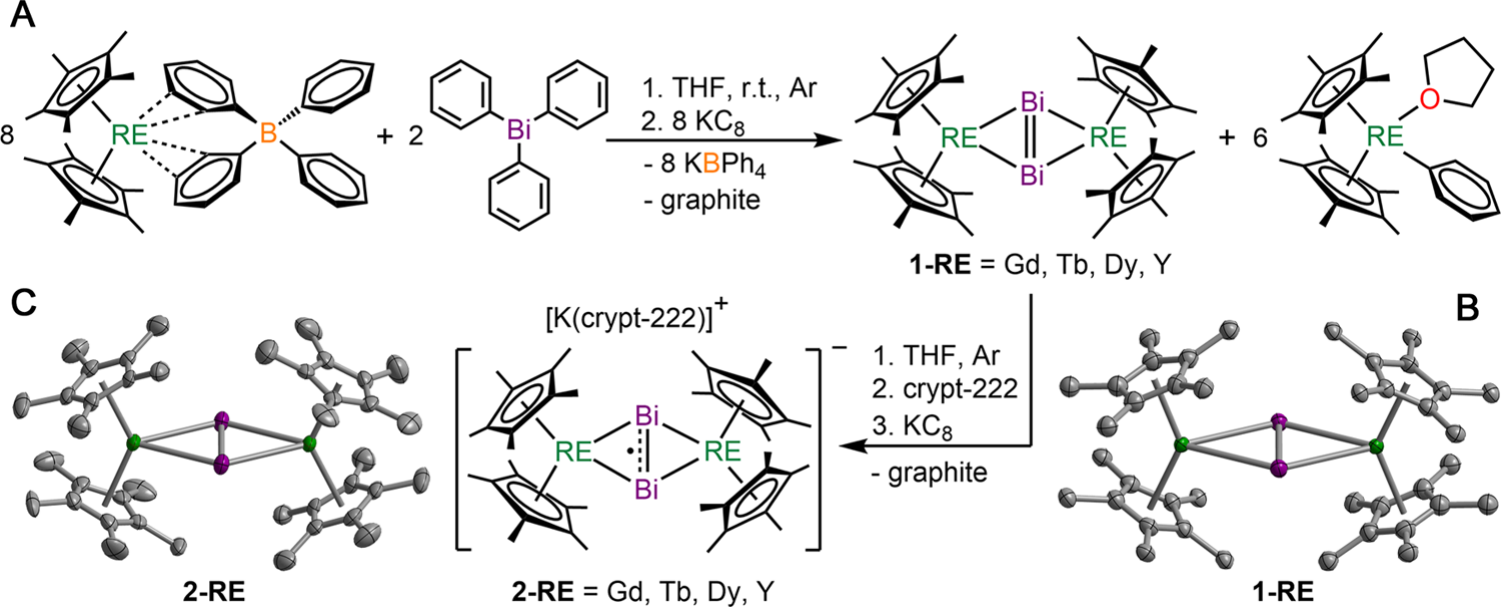
(A) Synthetic schemes for **1-RE** and **2-RE**. (B) Thermal ellipsoid plot of **1-Dy**. **1-Gd**, **1-Tb**, and **1-Y** are isostructural to **1-Dy**, drawn at the 50% probability level. (C) Thermal ellipsoid plot of the anion [(Cp*_2_RE)_2_(μ-η^2^:η^2^-Bi_2_^•^)]^−^ in a crystal of **2-Dy**, drawn at the 50% probability level. **2-Gd**, **2-Tb**, and **2-Y** are isostructural to **2-Dy**. Green, purple, and gray ellipsoids represent RE, Bi, and C atoms, respectively. H atoms and [K(crypt-222)]^+^ have been omitted for clarity. Selected interatomic distances (Å) and angles (deg) for **1-Gd**, **1-Tb**, **1-Dy**, and **1-Y**, respectively: Bi–Bi = 2.8549(9), 2.8528(11), 2.8418(10), 2.8419(7); mean RE–Bi = 3.2611(8), 3.2398(17), 3.2354(13), 3.2335(15); RE···RE = 5.8638(9), 5.8167(22), 5.8127(15), 5.8081(21); mean Cp*_centroid_-RE-Cp*_centroid_ = 134.59(4), 135.38(7), 135.20(5), 134.71(6). Selected interatomic distances (Å) and angles (deg) for **2-Gd**, **2-Tb**, **2-Dy**, and **2-Y**, respectively, for one of the two or three molecules in the unit cell for all complexes: Bi–Bi = 2.9310(11), 2.9405(6), 2.9450(13), 2.9366(6); mean RE–Bi = 3.2064(5), 3.1945(6), 3.1865(8), 3.1879(6); RE···RE = 5.7039(7), 5.6721(9), 5.6528(12), 5.6593(9); mean Cp*_centroid_-RE-Cp*_centroid_ = 131.99(1), 131.19(5), 131.80(4), 131.13(8).

The rare earth tetraphenyl salts Cp*_2_RE(BPh_4_) are extremely well suited for insertion, salt-metathesis, and reduction reactions, owing to a weakly, equatorially coordinating (BPh_4_)^−^ to the metal ion, and thus, readily displaceable.^38^ Recently, we have shown that the Tb^III^ and Dy^III^ congeners are excellent lanthanide sources to bind bismuth ions to the metal centers under reducing conditions at elevated temperatures, giving rise to the first organometallic lanthanide–bismuth clusters.^36^ Taking a stirring THF solution of eight equivalents of Cp*_2_RE(BPh_4_) (RE = Gd, Tb, Dy, and Y) and two equivalents of triphenylbismuth at room temperature under argon, and adding eight equivalents of potassium graphite, KC_8_, produces the toluene-soluble neutral compounds **1-RE**. KC_8_ addition generates highly reactive species that initiate a reduction of Bi^III^ to Bi^–I^, allowing the generation of the bimetallic complexes **1-RE**. The poorly soluble byproducts are KBPh_4_ and graphite precipitates, and Cp*_2_RE(Ph)(THF) which is readily removed due to solubility in hexane.^36^ Crystals of **1-RE** suitable for X-ray analysis were grown from concentrated toluene solutions at −35 °C. One-electron reduction of **1-RE** with KC_8_ in the presence of 2.2.2-cryptand in THF at −78 °C afforded the Bi_2_^3–•^ radical-bridged complexes **2-RE**, Figure 1, in approximately 50% yield. Crystals of **2-RE** suitable for X-ray analysis were grown from the diffusion of diethyl ether into THF solutions of **2-RE** at −35 °C.

Complexes **1-RE** are isostructural and crystallize in the monoclinic space group *P*2_1_, Tables S1–S4. Despite the absence of an inversion center, **1-RE** feature a nearly coplanar arrangement of the side-on bridging Bi_2_^2–^ unit and the two lanthanide centers, Figure 1, with the RE–Bi–Bi–RE dihedral angles ranging between 178.80 and 179.99°. Each lanthanide is eightfold-coordinated by two η^5^-Cp* rings and a bridging Bi_2_^2–^ moiety. The Bi–Bi distances range from 2.8419(7) Å for **1-Gd** to 2.8549(9) Å for **1-Y**, indicating a Bi–Bi double bond,^39^ and that differences in ionic radii between different RE ions cause only a small impact on the positions of the bismuth nuclei. We note that the Bi–Bi distances in **1-Tb** and **1-Dy** (2.8528(11) and 2.8418(10) Å, respectively) are substantially shorter than the corresponding distances in the lanthanide–bismuth clusters [K(THF)_4_]_2_[Cp*_2_Ln_2_Bi_6_] (3.0352(6) and 3.0313(7) Å for Ln = Tb and Dy, respectively), where the bond order was assigned to one.^36^ The mean Ln–Bi distances range from 3.2611(8) Å for **1-Gd** to 3.2335(2) Å for **1-Y**, as a direct consequence of the lanthanide contraction. The average Cp*_centroid_-RE-Cp*_centroid_ angle for all **1-RE** complexes falls in a narrow range of 134.59(4) to 135.38(7)°, similar to those found in other complexes containing [Cp*_2_RE]^+^ moieties.^40^ Compounds **1-RE** are isostructural to the Sm variant which requires a vastly different synthetic pathway involving the unsolvated, divalent, single-electron-transfer (SET) reagent Cp*_2_Sm^II^ acting simultaneously as reductant and lanthanide source.^30^ An analogous synthetic route with Gd, Tb, Dy, and Y is not possible owing to the inaccessibility of the required divalent reagents.

Compounds **2-Gd** and **2-Tb** crystallize in the monoclinic space group *I*2/*a* and *C*2/*c*, respectively, as opposed to **2-Dy** and **2-Y** which crystallize in the *C*2/*m* space group. The crystal structures exhibit two independent centrosymmetric molecules for all **2-RE** in spite of their different space groups, Figure S1 and Tables S5–S8. The [(Cp*_2_RE)_2_(μ-η^2^:η^2^-Bi_2_^•^)]^−^ anion features eight-coordinate metal ions where each is bound to two η^5^-Cp* rings and a bridging Bi_2_^3–•^ radical anion, and is paired with a noninteracting [K(crypt-222)]^+^ cation. The Bi–Bi distances are 2.9310(11) (**2-Gd**), 2.9405(6) (**2-Tb**), 2.9450(13) (**2-Dy**), and 2.9366(6) (**2-Y**) Å for one of the two molecules, respectively, and fall in between the bond lengths of 2.8 and 3.1 Å found for single and double bonds, respectively.^39^ The Bi ions are approximately 0.1 Å further apart from each other compared to **1-RE**, indicating a bond order of 1.5 and suggesting an oxidation state of −1.5 for each bismuth ion.^39^ This constitutes the first crystallographic evidence of a new diatomic radical species captured between d-/f-block metal ions; the only other Bi_2_^3–•^-containing compound was recently isolated with gallium ions, which features a slightly shorter Bi–Bi bond of 2.9266(3) Å, possibly owing to an end-on coordination of the dibismuth bridge^37^ contrary to the side-on mode apparent in **2-RE**. The mean RE–Bi distances are 3.2064(5) (**2-Gd**), 3.1945(6) (**2-Tb**), 3.1865(8) (**2-Dy**), and 3.1879(6) (**2-Y**) Å, and are diminished relative to those in **1-RE**, potentially due to both a larger negative charge on Bi_2_^3–^ increasing electrostatic interactions, and more covalency in the RE–Bi bonds. Here, the shorter RE–Bi bond lengths and elongated Bi–Bi diagonal in the RE_2_Bi_2_ rhombic core lead to substantially decreased RE···RE distances in **2-RE** compared to **1-RE**, Figure 1. As a result, the **2-RE** complexes possess smaller average Cp*_centroid_-RE-Cp*_centroid_ angles than **1-RE** due to more steric crowding.

### Spectroscopy and Electronic Structure

**Figure 2:**
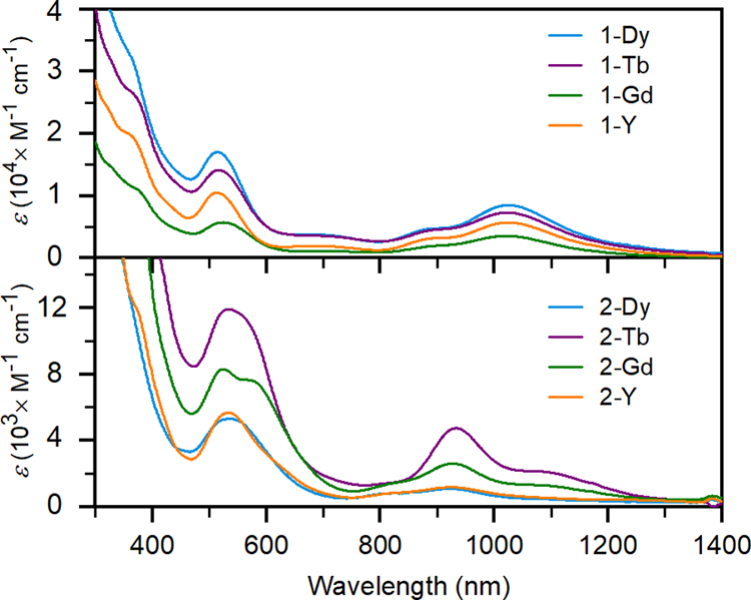
UV–vis spectra of THF soultions of complexes **1-RE** and **2-RE**.

The ultraviolet–visible (UV–vis) spectra of complexes **1-RE** in THF show two intense absorption bands around 510 and 1020 nm, with strongly increasing absorption below 400 nm (Figure 2). Notably, the UV–vis spectrum of the bare Bi_2_^2–^ anion in [K(crypt-222)]_2_Bi_2_ differs substantially.^41^ The increase below 400 nm arises from transitions on the Cp ligands,^20^ while the band at 510 nm corresponds to the electric-dipole allowed π → π* transition on the Bi_2_^2–^ fragment, as characterized previously for dibismuthenes.^37^ The UV–vis spectra of **2-RE** are significantly more intense than of **1-RE**, by a factor of about 6 to 8, and display different features. The higher-energy band is red-shifted to approximately 550 nm and is significantly broader, with evidence of multiple features for **2-Gd** and **2-Tb**, and the lower-energy bands have a different intensity pattern, with the largest feature blue-shifted to 930 nm, though intensity remains out toward 1200 nm as for **1-RE**. It is not clear, a priori, if these are the same features as observed in **1-RE**.

**Figure 3:**
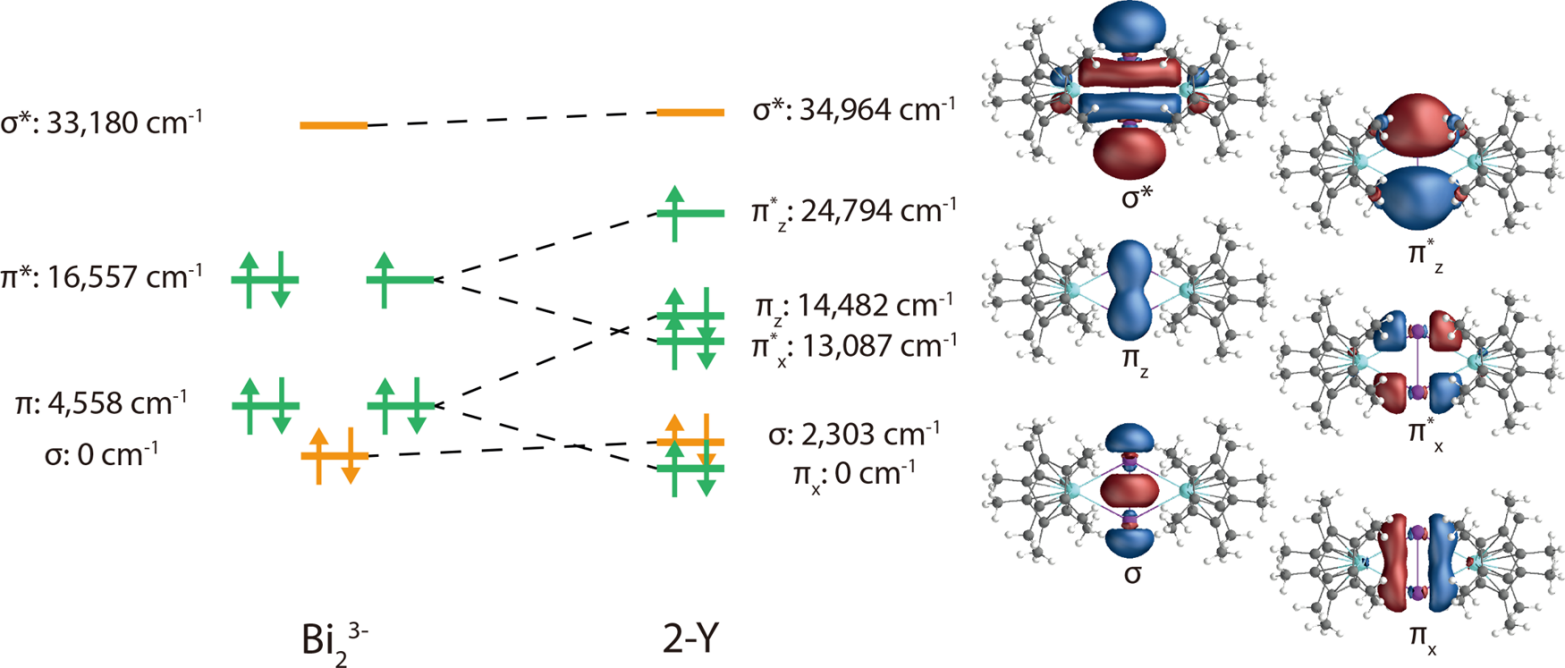
Quantitative MO diagram of the 6p valence space for the Bi_2_^3–^ anion isolated (left) and in **2-Y** (right), with energies derived from CASCI-MCPDFT calculations. The effective relative energies of the Y(4d)-based orbitals included in the active space are 33,242 cm^–1^ (4d_*x*^2^–*y*^2^_) and 34,087 cm^–1^ (4d_*y*^2^_), and are not shown for clarity. Isosurfaces drawn at 0.05 a.u.

To understand the electronic spectra of the **1-RE** and **2-RE** compounds, we first calculate the electronic structure of **1-Y** and **2-Y** along with the isolated Bi_2_^3–^ anion (at its XRD geometry from **2-Y**) using multiconfigurational methods. For all calculations on **1-RE** and **2-RE** compounds herein, we use a coordinate frame where RE–RE vector is *x*, the Bi–Bi vector is *y*, and the normal to this plane (tangential to Cp*_centroid_-RE-Cp*_centroid_) is *z* (Figure S2). State-averaged complete active space self-consistent field (SA-CASSCF) calculations were performed for the Bi_2_^3–^ anion, considering two roots of the *S* = 1/2 ground state with a (9,6) active space consisting of the 6p atomic orbitals, and show that the ground state is a doubly degenerate σ^2^ π^4^π^*3^ configuration (Figure 3). Excited states were then obtained with a complete active space configuration interaction (CASCI) expansion of the lowest seven roots in the optimized ground state orbitals, followed by corrections for dynamic correlation using multiconfigurational pair-density-functional theory (MCPDFT; Table S9).^42^ From these calculations, we can approximate a quantitative MO diagram accounting for electron correlation (Figure 3).

**Figure 4:**
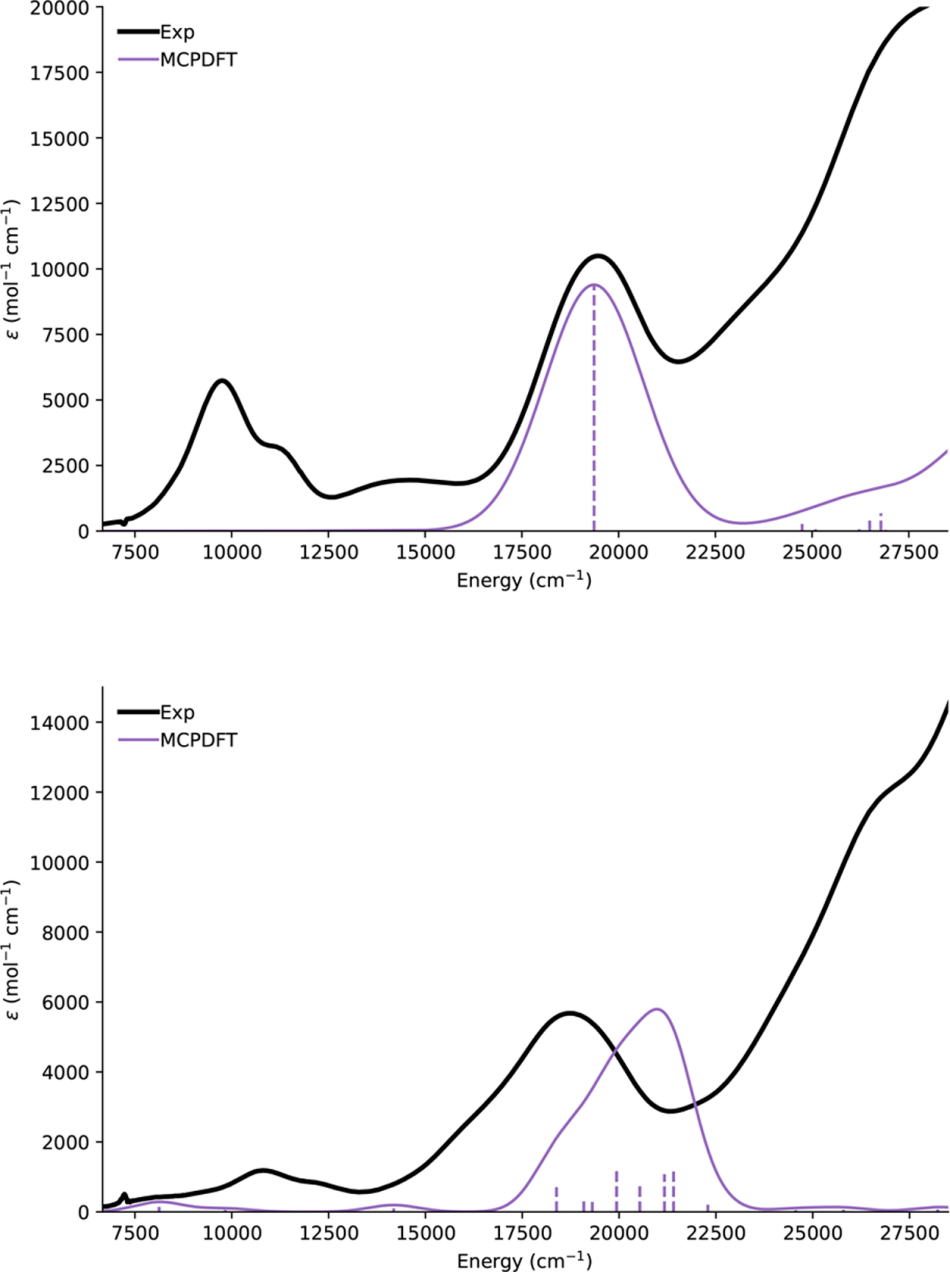
Calculated UV–vis spectra of **1-Y** (top) and **2-Y** (bottom). Calculated intensities are velocity gauge Einstein coefficients, convoluted with Gaussian functions with a linewidth of 3000 and 1500 cm^–1^, respectively, and scaled to a similar magnitude as the experimental molar absorption coefficients.

We then performed SA-CASSCF calculations for **1-Y** with 12 singlet roots and 5 triplet roots in an (8,9) active space consisting of the Bi_2_ 6p orbitals and three Y(4d)/Bi(6d) hybrids (Table S10). Adding corrections for dynamic correlation using MCPDFT and including spin–orbit (SO) coupling (henceforth we refer to this method as SA-CASSCF-MCPDFT-SO), shows the ground state is best described as a singlet configuration Bi_2_(σ^2^π_*x*_^2^π_x_^*2^π_*z*_^2^) with the first excited state being the triplet Bi_2_(σ^2^π_*x*_^2^ π_x_^*2^ π_z_^1^ π_z_^*1^) at ca. 10,400 cm^–1^. The most significant interactions between Y^III^ and the Bi_2_^2–^ anion in the ground state are via the doubly occupied Bi_2_ 6p π_*x*_ and π_x_^*^ orbitals, which have σ* (Bi 6p–Y 4d) and π_*x*_ (Bi 6p–Y 4d) character, respectively, regarding the Y^III^ and Bi_2_^2–^ interaction (Table S10). Calculation of the optical transition intensities shows only one intense band at 19,400 cm^–1^ (ca. 515 nm, Figure 4), which agrees well with the experimental peak at 510 nm, and corresponds to a singlet → singlet (π_*z*_ → π_z_^*^ ) transition, confirming our original assignment. The singlet → triplet (π_*z*_ → π_z_^*^) transition at 10,400 cm^–1^ (ca. 962 nm) is in good agreement with the presence of an experimental transition at ca. 1000 nm; however, the calculated intensity is so weak that it cannot even be seen on the calculated spectrum (Figure 4); the larger experimental intensity is likely due to SO effects that have not been captured in our calculations, enhancing this spin-forbidden transition.

For **2-Y**, we performed SA-CASSCF calculations with 16 doublet roots and 7 quartet roots for a (9,8) active space consisting of the Bi_2_ 6p orbitals and two Y(4d) nonbonding orbitals (Table S11). We find the ground state is best described as a doublet configuration Bi_2_(σ^2^π_*x*_^2^π_x_^*2^π_*z*_^2^π_z_^*1^), with the first excited state being a doublet Bi_2_(σ_2_π_*x*_^2^π_x_^*2^π_*z*_^2^σ^*1^) at ca. 10,900 cm^–1^. Using the relative energies of the doublet excitations (Table S12) we can approximate a quantitative MO diagram (including electron correlation energy) for **2-Y** (Figure 3). The bonding interactions between Y^III^ and Bi_2_^3–^ are remarkably similar to **1-Y**, being dominated by the doubly occupied Bi_2_ π_*x*_ and π_x_^*^ orbitals having σ* and π_*x*_ characters, respectively (Table S11). The SA-CASSCF-MCPDFT-SO-calculated UV–vis absorption spectrum is in reasonably good agreement with the experimental spectrum (Figure 4). The region around 18,000–22,000 cm^–1^ (ca. 450–550 nm) is far more featured than for **1-Y**, in agreement with experiment, including a longer tail out toward 16,000 cm^–1^ (ca. 625 nm). The most intense transition in this region, calculated at 19,900 cm^–1^ (ca. 503 nm), is best described as an SO coupled singlet → singlet/triplet ( π_x_^*^ → 4d_*x*^2^–*y*^2^_) ligand to metal charge-transfer (LMCT) transition. The next most intense transitions are calculated at 21,400, 21,200, 20,500, and 18,400 cm^–1^ (ca. 467, 472, 488, and 543 nm, respectively), and are complicated SO transitions arising from partial MLCT singlet → singlet/triplet (π_x_^*^/σ/π_*z*_ → σ*/4d_*x*^2^–*y*^2^_/4d_*y*^2^_) states. It is clear that the far richer spectrum in the 500–550 nm region is substantially different in nature from **1-Y**, and is indicative of more significant SO effects than observed in **1-Y**, which likely contributes to the enhanced intensity in this region. The transition calculated at 14,200 cm^–1^ (ca. 704 nm) is the singlet → singlet (π_*z*_ → π_z_^*^) transition, which supports the large experimental intensity of the tail in this region; this feature is strongly red-shifted compared to that calculated for **1-Y** (ca. 515 nm). The feature predicted at 9,800 cm^–1^ (ca. 1020 nm) is the singlet → singlet (π_z_^*^ → σ*) transition.

### Magnetism and Electronic Structure

**Figure 5:**
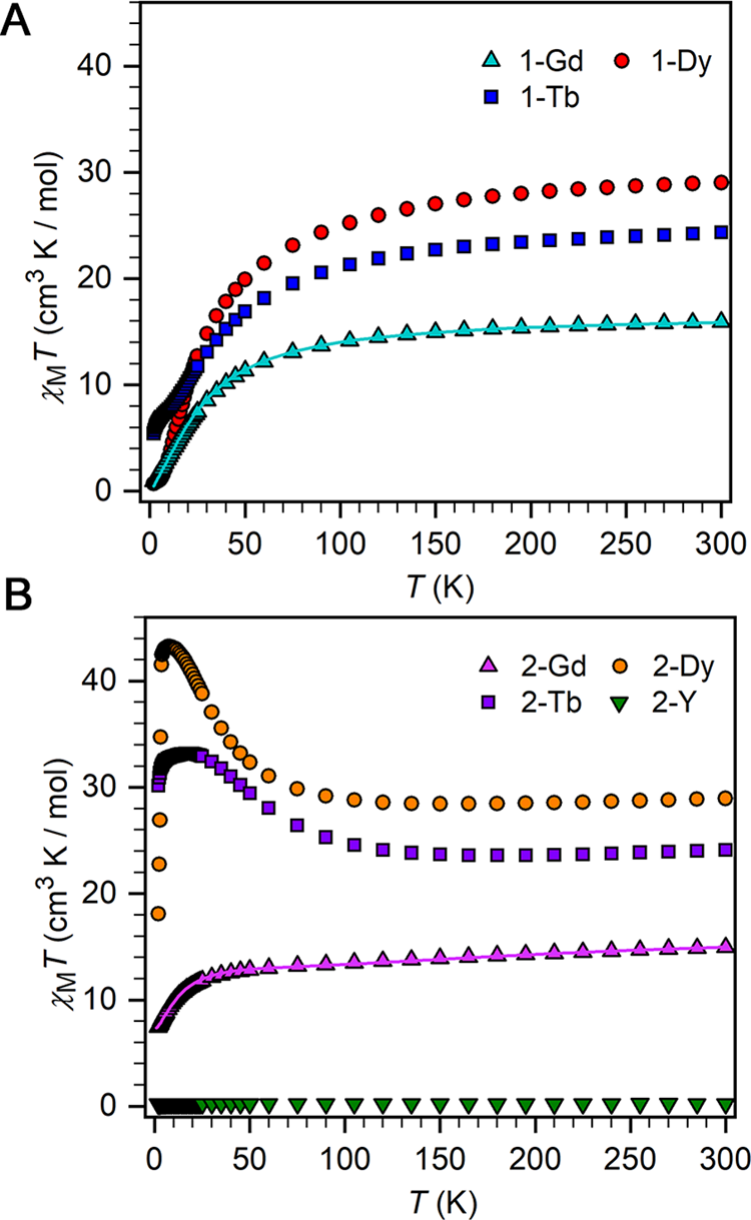
Temperature dependence of the χ_M_*T* product for polycrystalline samples of **1-RE** (A) and **2-RE** (B) under a 1000 Oe applied dc field. Solid lines for **1-Gd** and **2-Gd** are best fits to the data as described in the text.

Static magnetic susceptibility measurements on a polycrystalline sample of **2-Y** between 2 and 300 K under a 1000 Oe direct current (dc) field show that the product of molar magnetic susceptibility and temperature (χ_M_*T*) is 0.23 cm^3^ K/mol at 300 K and is relatively temperature-independent (Figures 5 and S3). This value is consistent with, although lower than expected for, a typical organic radical with *S* = 1/2 and *g* = 2 (0.375 cm^3^ K/mol). Of the only two other structurally characterized bismuth radicals, one a monomer and one a dimer, both have substantially anisotropic *g*-values (monomeric: *g*_1_ = 1.621, *g*_2_ = 1.676, *g*_3_ = 1.832; dimeric: *g*_1_ = 3.12, *g*_2_ = 2.01, *g*_3_ = 1.78) and the monomeric example also shows a low χ_M_*T* value of 0.27 cm^3^ K/mol.^37,43^ The *g*-anisotropy and low χ_M_*T* values are indicative of significant magnetic anisotropy resulting from strong SO coupling of 6p orbitals. We have attempted to collect electron paramagnetic resonance spectra for **2-Y** to confirm the electronic *g*-value, but no spectrum could be obtained (experiments spanning 10 K to room temperature, both frozen solution and pure solid); we suspect this is due to fast spin relaxation due to strong SO coupling.

Measurement of χ_M_*T* for a polycrystalline sample of **1-Gd** shows a value of 15.87 cm^3^ K/mol at 300 K, which is consistent with the expected value of 15.76 cm^3^ K/mol for two uncoupled Gd^III^ ions. χ_M_*T* steadily decreases as the temperature is lowered, hastening below 150 K to reach 0.92 cm^3^ K/mol at 2 K (Figure 5), suggesting antiferromagnetic coupling between the two Gd^III^ ions. The magnetization (*M*) vs field data are linear and show overlapping 2, 4, and 6 K isotherms, supporting the assignment of an antiferromagnetic ground state (Figure S4). Simultaneously fitting the χ_M_*T* and the *M* data to the isotropic Heisenberg spin Hamiltonian *Ĥ* = −2*Ŝ̂*_Gd1_·*Ŝ*_Gd2_ + μ_B_*g*(*Ŝ*_Gd1_ + *Ŝ*_Gd2_)·*B⃗* in the PHI program^44^ gives *J* = −1.143(4) cm^–1^ and *g* = 2.071(1). The magnitude of this Gd–Gd coupling constant is unprecedented for gadolinium complexes containing diamagnetic bridges which typically show |*J*| < 0.1 cm^–1^;^45^ the next largest Gd–Gd exchange coupling is found for an aromatic arene bridge (*J* = −0.664 cm^–1^; Table S13).^46^ Calculation of the exchange coupling employing broken-symmetry DFT suggests that *J* = −1.36 cm^–1^ using the B3LYP density functional (Table S14), in very good agreement with experiment. For **2-Gd**, the χ_M_*T* product at 300 K is 14.95 cm^3^ K/mol, lower than the anticipated value for two noninteracting Gd^III^ ions and a radical (16.14 cm^3^ K/mol), and decreases as the temperature is lowered, reaching 7.44 cm^3^ K/mol at 2 K (Figure 5). The *M* vs field isotherms (Figure S4) are clearly separated at low fields with the standard ordering (2 > 4 > 6 K), are nonlinear at intermediate fields, and appear to overlap and increase linearly at high fields. While at face value the χ_M_*T* data for **1-Gd** and **2-Gd** are similar, the former clearly tends toward zero at the lowest temperatures, indicating a nonmagnetic (antiferromagnetic) ground state, while the latter trend toward 7.44 cm^3^ K/mol suggesting an *S* = 7/2 ground state (expected 7.88 cm^3^ K/mol for *g* = 2). Coupled with the magnetization data, which also suggest a magnetic ground state for **2-Gd**, the presence of the radical spin and its interaction with the Gd^III^ ions is unmistakable. Based on literature precedent, we can safely assume the Gd-radical exchange coupling is stronger than the Gd–Gd exchange coupling;^16,19,38,47,48^ that is, assuming a spin Hamiltonian of the form *Ĥ* = −2*J*_1_ (*Ŝ*_Gd1_·*Ŝ*_rad_ + *Ŝ*_rad_·*Ŝ*_Gd2_) – 2*J*_2_*Ŝ*_Gd1_·*Ŝ*_Gd2_ + μ_B_*g*(*Ŝ*_Gd1_ + *Ŝ*_rad_ + *Ŝ*_Gd2_)·*B⃗*, we can assume |*J*_1_| > |*J*_2_|. In this regime, the experimental χ_M_*T* data provide direct evidence of antiferromagnetic interactions between all spins: if either were ferromagnetic, the plot would show an upturn at low temperatures owing to the population of a high-spin (*S* = 15/2) ground state. Simultaneously fitting the χ_M_*T* and *M* data give *J*_1_ = −15.9(2) cm^–1^, *J*_2_ = −1.92(3) cm^–1^ and *g* = 2.069(2). Even though the Gd-radical interaction *J*_1_ is much larger than the Gd–Gd interaction *J*_2_, the large spin *S* = 7/2 of Gd^III^ vs *S* = 1/2 of the radical means *J*_2_ has a strongly frustrating effect, leading to an *S* = 7/2 ground state with *S* = 5/2, 9/2 and 3/2 all lying within 5 cm^–1^. Hence, it is likely that the magnetic susceptibility experiment is not accurate enough to precisely quantify the energies of these excited states. Broken-symmetry DFT calculations suggest that *J*_1_ = −15.5 and *J*_2_ = −2.1 cm^–1^ (Table S14), which are in very good agreement with the values obtained from the fit of the experimental data. This value of *J*_1_ is smaller than that found in N_2_^3–^ bridged Gd dimers, for example both [K(18-C-6)]{[((Me_3_Si)_2_N)_2_(THF)Gd]_2_(μ-η^2^:η^2^-N_2_)} and K{[((Me_3_Si)_2_N)_2_(THF)Gd]_2_(μ-η^2^:η^2^-N_2_)} have *J*_1_ ca. −27 cm^–1^,^16,48^ and [K(crypt-222)(THF)][(Cp_2_^tet^Gd)_2_(μ-η^2^:η^2^-N_2_)] (Cp^tet^ = tetramethylcyclopentadienyl) has *J*_1_ = −20 cm^–1^.^19^ It is possible that the smaller magnitude of *J*_1_ here owes to the more diffuse character of the Bi 6p SOMO in **2-Gd** vs. the N 2p SOMO in those other examples.

**Figure 6:**
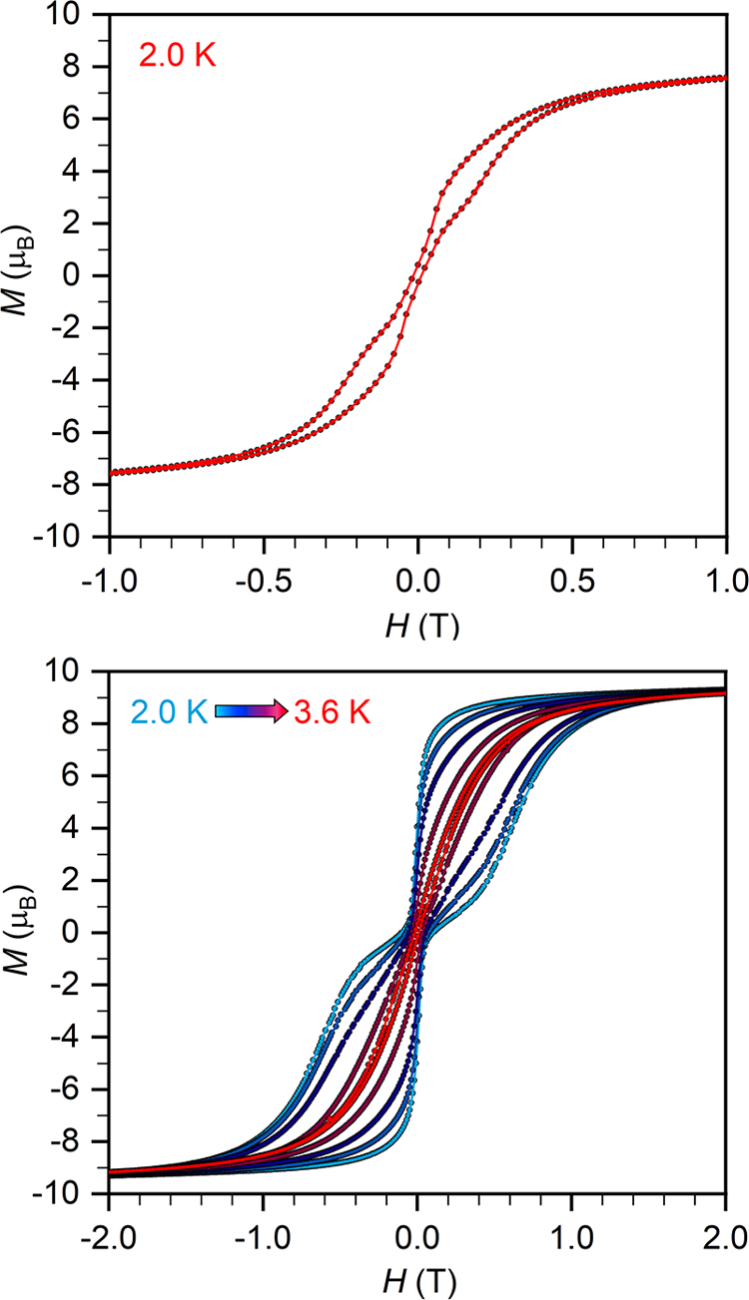
Variable-field magnetization (*M*) data collected for **2-Tb** (top) and **2-Dy** (bottom) at a sweep rate of 100 and 50 Oe/s, respectively. Solid lines are a guide to the eye.

For the complexes containing anisotropic lanthanide ions, the χ_M_*T* values at 300 K are 24.34 (**1-Tb**), 24.11 (**2-Tb**), 29.03 (**1-Dy**), and 28.95 (**2-Dy**) cm^3^ K/mol (Figure 5), are in reasonable agreement with the expected values for two noninteracting lanthanides (**1-RE**) and for **2-RE** including a radical spin center, respectively. We note that the values for **2-RE** are all lower than those for **1-RE**, which is counter-intuitive assuming negligible Ln-radical interactions, and hence is the first indication of antiferromagnetic interactions in **2-RE**. With decreasing temperature, distinct trends of the χ_M_*T* products are observed for the Bi_2_^2–^ and Bi_2_^3–^ compounds. Complexes **1-Tb** and **1-Dy** display a quick decline in χ_M_*T*, largely owing to depopulation of crystal field (CF) states of the ground SO manifolds, but antiferromagnetic exchange coupling could also be a contributing factor. In contrast, the χ_M_*T* values for **2-Tb** and **2-Dy** exhibit a slight decrease upon lowering the temperature to reach shallow minima at 195 and 165 K, respectively, followed by clear rises to 33.1 and 43.2 cm^3^ K/mol at 8 and 20 K, respectively. This latter behavior can only occur due to strong lanthanide-radical coupling and is characteristic of radical-bridged di-lanthanide complexes.^21^ However, a simple interpretation of the type of interactions is precluded due to the unquenched orbital angular momentum of Tb^III^ and Dy^III^. The magnetization curves of **1-Tb** and **1-Dy** have a striking S-shape at low temperatures (Figure S5), with a gradual rise of the magnetization at low magnetic fields and inflection points at 4.2 and 2.4 T, respectively, which strongly suggests a sizeable antiferromagnetic coupling between the two lanthanide centers. In contrast, the *M*–*H* curves obtained for **2-Tb** and **2-Dy** (Figure S6) exhibit a steep rise at low fields, suggesting a ground state with a large magnetic moment, followed by a gradual increase at higher fields up to 7 T, indicative of large magnetic anisotropy. Waist-constricted hysteresis loops are observed at low temperatures for both **2-Tb** and **2-Dy** (Figure 6), remaining open up to 3.6 K for **2-Dy**.

To probe the underlying magnetization dynamics, we performed variable-frequency alternating current (ac) magnetic susceptibility measurements. Only a shoulder of a peak in the out-of-phase magnetic susceptibility (χ_M_″) was observed for **1-Dy** (Figure S11), indicating relatively fast magnetization dynamics. This contrasts to a series of pnictogen-bridged tri-lanthanide compounds, [Cp_2_^Me^Dy(μ-E(H)Mes)]_3_ (E = P, As, Sb; Cp^Me^ = methylcyclopentadienyl; Mes = mesityl), that show slow magnetization dynamics on the timescale of a.c. susceptibility measurements, with increasing effective spin-reversal barriers increasing from P, As, to Sb.^26^ For **1-Tb**, no out-of-phase (χ_M_″) signals were observed (Figure S12). Here, we suspect that strong intramolecular antiferromagnetic coupling between the two Ln^III^ centers, as suggested by the static magnetic measurements, results in a nonmagnetic ground state.

**Figure 7:**
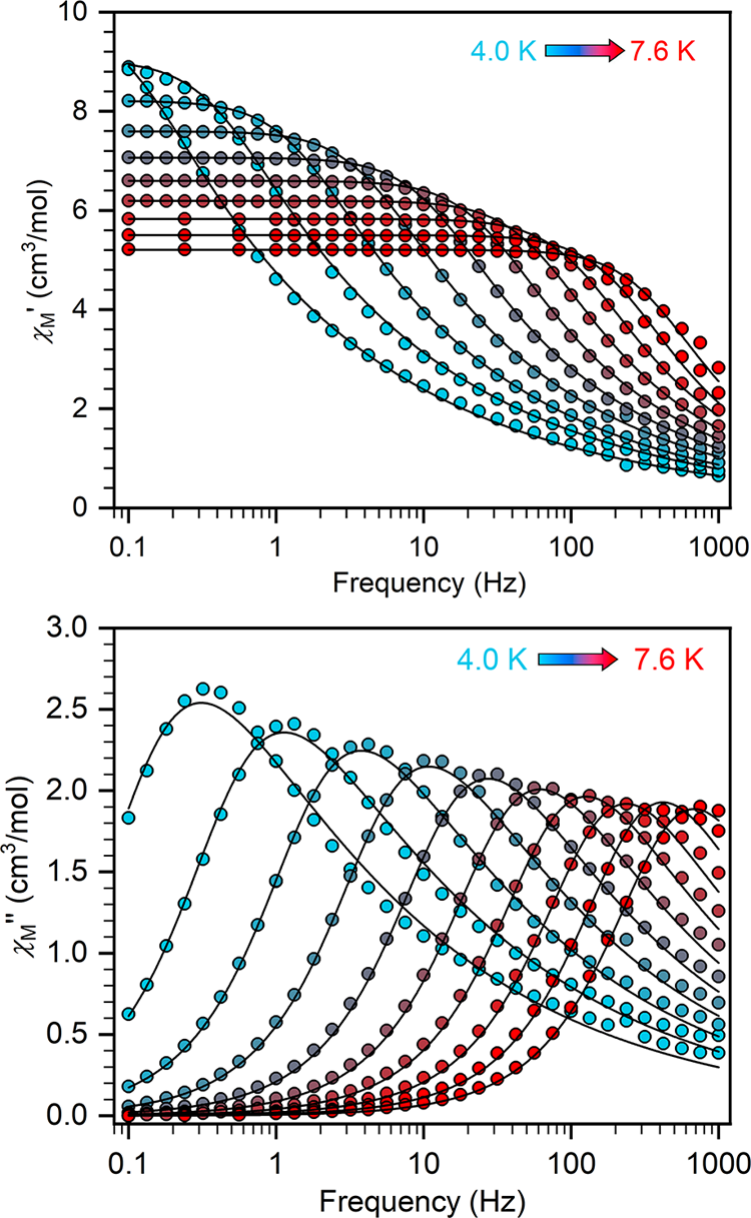
Dynamic magnetic susceptibility data. Variable-temperature, variable-frequency in-phase (χ_M_′) and out-of-phase (χ_M_″) ac magnetic susceptibility data collected under a zero applied dc field for **2-Dy** from 4.0 to 7.6 K. Solid lines indicate the fits to the Cole–Davidson model.

In contrast, clear χ_M_″ signals are observed for both **2-Dy** and **2-Tb** under zero applied dc field (Figures 7 and S13). Nevertheless, **2-Tb** shows temperature-independent χ_M_″ peaks at low temperatures in zero dc field (Figure S13), consistent with quantum tunneling of the magnetization (QTM). This can be suppressed by applying a dc field, and we found the optimum dc field to be 1500 Oe (Figure S14), which results in much stronger temperature dependencies of χ_M_″ within the range of 4 and 7 K (Figure S15). However, a generalized Debye function was insufficient to model these broad peaks well, suggesting a substantially skewed distribution of relaxation times; this likely originates from the presence of two inequivalent molecules in the crystal structure and the highly disordered Cp* ligands. Satisfactory fits could be obtained with the Cole–Davidson model (eq 1; Figures 7 and S15–S17),^49^ where the relaxation time of the sample is related to the fitted value of τ_AC_ in eq 1 via the logarithmic moment, giving τ = *e*^(Ln[τAC]+ψ(β)+Eu)^ (where ψ(*x*) is the digamma function, ψ′(*x*) is the trigamma function and Eu is Euler’s constant),^50,51^ and where β reports on the breadth and skewness of the distribution of relaxation times.^49^ Here, we find β values between 0.25 and 0.52 (Tables S15 and S16), which correspond to approximately similar distributions as the generalized Debye model for α values between 0.58 and 0.28, respectively. A fit of the relaxation data for **2-Dy** to the Orbach expression, τ^–1^ = 10^–*A*^ exp(−*U*_eff_/*kT*), shows good agreement with experiment and gives *U*_eff_ = 38(15) cm^–1^ and τ_0_ = 10^–7(2)^ s (Figure S18), while for **2-Tb**, we obtain *U*_eff_ = 51(26) cm^–1^ and τ_0_ = 10^–9(3)^ s (Figure S19).
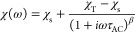
1

Due to unquenched orbital angular momentum in the ground states for **1-Tb/Dy** and **2-Tb/Dy**, the analysis is significantly more complicated and neither simple model Hamiltonians nor DFT calculations are appropriate here. SA-CASSCF-SO calculations with an (8,7) active space (4f orbitals only) for the isolated Tb^III^ ions in **1-Tb** or a (9,7) active space for the Dy^III^ ions in the case of **1-Dy** give us direct access to the CF splitting of the ground SO manifolds for each ion. For both **1-Tb** and **1-Dy**, the bis-Cp* ligands dictate the magnetic anisotropy at each Ln ion, just like for [Tb(Cp^ttt^)_2_][B(C_6_F_5_)_4_]^52^ and [Dy(Cp^ttt^)_2_][B(C_6_F_5_)_4_],^53^ and hence the Ln^III^ ions in these compounds have parallel Ising-like ground (pseudo-)doublets that are well described by *m*_J_ = ±6 functions with *m*_J_ = ±5 excited states at ca. 120 cm^–1^ for **1-Tb** (Table S17), and by *m*_J_ = ±15/2 functions with *m*_J_ = ±13/2 excited states at ca. 180 cm^–1^ for **1-Dy** (Tables S18 and S19). In this sense, the magnetic anisotropy induced by the ligand framework has a similar effect for Tb^III^ and Dy^III^ ions, as expected due to sharing similar 4f electron densities for their *m*_J_ states.^54^

Given the well-isolated ground doublets, the low-temperature magnetic data can be approximated by an Ising Hamiltonian considering two pseudo-spin *S* = 1/2 states: *Ĥ* = −2*J*_*z*_*Ŝ*_*z*_Ln1__*Ŝ*_*z*_Ln2__ + μ_B_*g*_*_z_*_*B*_*z*_ (*Ŝ*_*z*_Ln1__ + *Ŝ*_*z*_Ln2__). While this approximate model is not detailed enough to allow us to fit the data, the inflection points in the magnetization data can be replicated with *J*_*z*_ = −32 cm^–1^ for **1-Tb** (with *g_z_* = 17.95 from SA-CASSCF-SO; Figure S7) and *J_z_* = −22 cm^–1^ for **1-Dy** (with *g_z_* = 19.50 from SA-CASSCF-SO; Figure S8). Crucially, owing to the parallel Ising ground (pseudo-)doublets for both **1-Tb** and **1-Dy**, *J*_*z*_ must be antiferromagnetic to replicate the form of the low-temperature magnetization data.

The magnetic properties of **2-Tb** and **2-Dy** are further complicated by the presence of significant Ln^III^-radical interactions, and low-lying CF states. Ideally, we would use SA-CASSCF-SO to calculate the magnetic exchange, but even the minimal active space required is far too large to access all of the spin states for the full **2-Tb** and **2-Dy** molecules directly. Hence, we use SA-CASSCF-(MSCASPT2-)SO calculations to parameterize the exchange interactions between a Ln^III^-radical pair (along with CF and SO effects at Ln^III^, eq 2), and then build a model Hamiltonian of the full complex; see Supporting Information and refs (20) and (55) for details. For **2-Tb**, we observe that (MSCASPT2 values given in braces), compared to the Tb^III^ ions in **1-Tb**, the axial *B*_2_^0^ CF parameter decreases (av. 544 down to 387 {350} cm^–1^) and that the equatorial CF parameter axial *B*_2_^+2^ increases (av. 576 up to 916 {757} cm^–1^) (Tables S20, S22, and S23), indicating the competitive effect the radical has on the axial field imposed by the bis-Cp motif. Considering the CF states alone (values given for Tb1), while the ground state is still well described as *m*_J_ = ±6, the first excited state can no longer be described as *m*_J_ = ±5, but rather is highly mixed, and is reduced in energy from 114 cm^–1^ down to 52 {30} cm^–1^ (Tables S17, S24, and S25). Similarly for **2-Dy**, the axial *B*_2_^0^ CF parameter decreases (av. 588 down to 310 {171} cm^–1^) and the equatorial CF parameter axial *B*_2_^+2^ increases (av. 705 up to 1033 {819} cm^–1^) relative to the Dy^III^ sites in **1-Dy** (Tables S21, S26 and S27). This results in the ground state changing from 90% *m*_J_ = ±15/2 to <50% *m*_J_ = ±15/2, and the first excited state being reduced in energy from 180 down to 37 {24} cm^–1^ (Tables S18, S19, and S29).

It is clear that the presence of the radical perpendicular to the local magnetic anisotropy axes induced by the Cp* ligands is detrimental to the magnetic anisotropy at the Tb^III^ and Dy^III^ sites in **2-Tb** and **2-Dy**; this mirrors recent findings for [(Cp_2_^Me4^Tb)_2_(μ-η^2^:η^2^-N_2_)]^−^.^56^ This is quite different from the situation for Cp^iPr5^LnI_3_LnCp^iPr5^,^20^ where the radical is co-parallel to the local magnetic anisotropy axes dictated by the Cp^iPr5^ ligands, and thus enhances the magnetic anisotropy; we have discussed this effect in a recent review.^17^
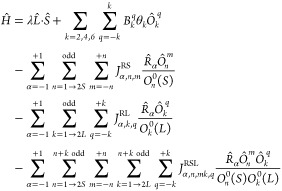
2

**Figure 8:**
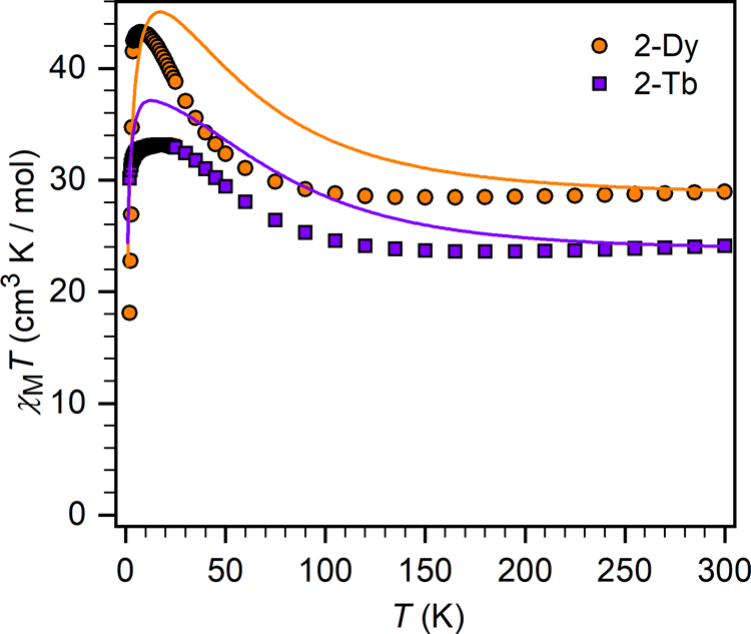
Temperature dependence of the χ_M_*T* product for polycrystalline samples of **2-Tb** and **2-Dy** under a 1000 Oe applied dc field. Solid lines are models based on SA-CASSCF-MSCASPT2-SO-calculated parameters.

Subsequently, we can use the calculated pair-wise Hamiltonian parameters to build a model Hamiltonian for the full molecules of **2-Tb** and **2-Dy** (see refs (20) and (55) for details), and subsequently calculate the temperature dependence of the magnetic susceptibility. The prediction obtained using the SA-CASSCF-SO parameters is not in good agreement with experiment for **2-Tb** (Figure S9), while it shows fair agreement for **2-Dy** (Figure S10). This poor-to-fair agreement is unsurprising given that CASSCF does not include dynamic correlation, which is known to be a crucial ingredient in calculation of exchange coupling.^57^ Using the parameters from MSCASPT2 calculations to correct for dynamic correlation leads to very good agreement for both **2-Tb** and **2-Dy** (Figure 8). Based on these results (Table S23), the dominant term in the exchange coupling for **2-Tb** is the tripartite *R̂*_α_*Ŝ*_α_*Ô*_4_^+4^ term (α ∈ *x,y,z*; note that for the *Ô*_*n*_^*m*^ operators in eq 2: *Ô*_1_^–1^ = *Ŝ*_*y*_, *Ô*_1_^0^ = *Ŝ*_*z*_, and *Ô*_1_^+1^ = *Ŝ*_*x*_) with an average coefficient of −131 cm^–1^, with the isotropic Heisenberg term (*R̂*_α_*Ŝ*_α_) a close second with av. −112 cm^–1^ (equivalent to *J* = −19 cm^–1^ in the standard Heisenberg −2*J* notation); there are numerous other tripartite terms with significant magnitudes including *R̂*_α_*Ŝ*_α_*Ô*_2_^–1^ (av. 88 cm^–1^), *R̂*_α_*Ŝ*_α_*Ô*_4_^–3^ (av. 63 cm^–1^) and *R̂*_α_*Ŝ*_α_*Ô*_2_^+2^ (av. 55 cm^–1^). We note that there are significant differences in both the exchange coupling and CF terms between the nonsymmetric Tb sites in the chosen molecule of **2-Tb** (Table S23); it appears that there is a trade-off between the exchange and CF terms induced by the radical, where the CF effects are larger for Tb1, and the exchange coupling terms are larger for Tb2. For **2-Dy** (Table S27), the dominant exchange terms are the isotropic Heisenberg terms (*R̂*_α_*Ŝ*_α_) at −121 cm^–1^ (*J* = −24 cm^–1^ in Heisenberg −2*J* notation), followed by numerous tripartite terms of the form *R̂*_α_*Ŝ*_α_*Ô*_*k*_^*q*^ which are first-rank isotropic in spin–spin coupling with higher-rank orbitally dependent terms, such as *R̂*_α_*Ŝ*_α_*Ô*_8_^–5^ (−102 cm^–1^), *R̂*_α_*Ŝ*_α_*Ô*_4_^+4^ (99 cm^–1^), *R̂*_α_*Ŝ*_α_*Ô*_6_^+4^ (−85 cm^–1^) and *R̂*_α_*Ŝ*_α_*Ô*_8_^+8^(−63 cm^–1^). Overall, these results paint a similar picture for **2-Tb** and **2-Dy**: isotropic first-rank spin–spin interactions dominate with significant anisotropies induced by the orbital angular momentum. For the latter part, higher-rank terms seem more important for **2-Dy** than for **2-Tb** (e.g., *k* = 4, 6, 8 appear toward the top of the list for **2-Dy**, while *k* = 2 and 4 appear at the top for **2-Tb**), which we believe is due to the larger orbital angular momentum for Dy^III^ compared to Tb^III^ (*L* = 5 cf. *L* = 3).

**Figure 9:**
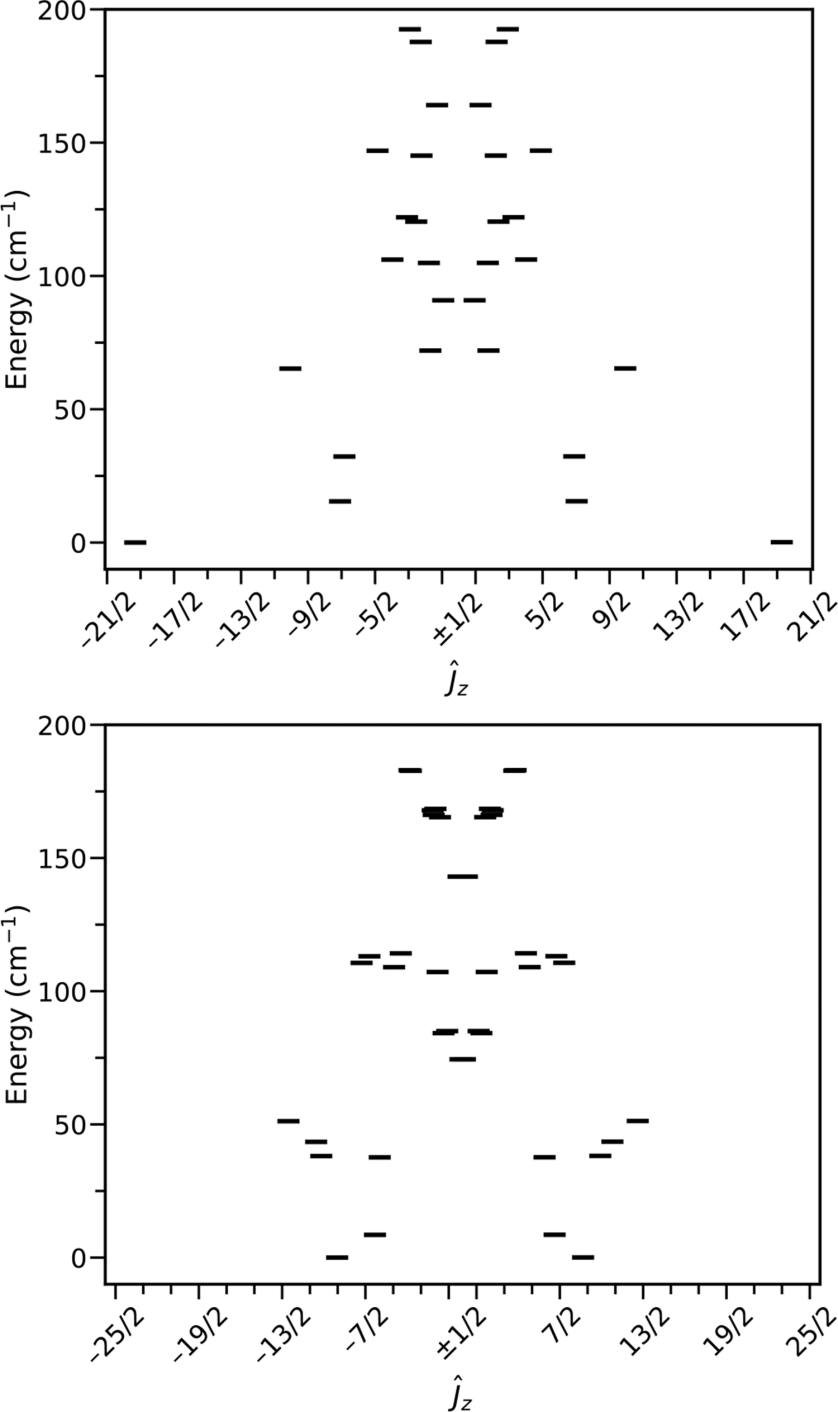
SA-CASSCF-MSCASPT2-SO-derived energy spectra for **2-Tb** (top) and **2-Dy** (bottom). Eigenstates of model Hamiltonian are shown in an 0.1 T field along the *z*-axis.

Based on the MSCASPT2-derived model Hamiltonians, we find the ground doublet for **2-Tb** is approximately 50% |*J* = 23/2, *m*_J_ = ± 23/2⟩ (where the projection *m*_J_ is defined along the molecular *z*-axis, perpendicular to the Tb_2_Bi_2_ plane) with several low-lying excited states having no more than 15% contribution from any one state. The competition between exchange coupling, CF and SO coupling effects leads to a very mixed low-energy spectrum (Figure 9), with low-angular-momentum states (known to facilitate magnetic relaxation) appearing as low in energy as 72 cm^–1^ above the ground state, in good agreement with the experimental energy barrier of 51(26) cm^–1^. The ground doublet for **2-Dy** is even more mixed and has leading terms 11% |*J* = 29/2, *m*_J_ = ±25/2⟩ + 8% |*J* = 29/2, *m*_J_ = ±19/2⟩ (other components <7%). This ground state is not at all well isolated, with the first excited state lying only 9 cm^–1^ higher in energy, and low-angular-momentum states appearing at 74 cm^–1^ above the ground state (Figure 9). This is in fair agreement with the experimental energy barrier of 38(15) cm^–1^; however, there are also four low-lying doublets predicted between 38 and 51 cm^–1^ that are more consistent with the experimental energy barrier.

Compared to analogous N_2_^3–^-bridged complexes [K(crypt-222)][(Cp_2_^Me4^Ln)_2_(μ-η^2^:η^2^-N_2_)],^19^ the magnetic properties of **2-Ln** show some notable differences: (i) the *U*_eff_ barriers are much smaller (*U*_eff_ = 51(26) and 38(15) cm^–1^ for **2-Tb** and **2-Dy** herein, cf. *U*_eff_ = 276(1)/564(17) and 108.1(2) for Ln = Tb and Dy, respectively); (ii) the coercive magnetic fields are much smaller (*H*_c_ ∼ 0 T at 2 K for both **2-Tb** and **2-Dy** herein, cf. *H*_c_ = 7.9 T at 10 K for Ln = Tb and *H*_c_ = 1 T at 5.5 K for Ln = Dy); and (iii) QTM is much more efficient in **2-Ln** than in [(Cp_2_^Me4^Ln)_2_(μ-η^2^:η^2^-N_2_^•^)]^−^, as observed by the large zero-field steps in the hysteresis loops here (Figure 6), but absent for the N_2_^3–•^-bridged complexes (which goes some way to explaining the smaller values of *H*_c_).^19^ The only similarity is that the Tb^III^ analogues both show larger *U*_eff_ than their Dy^III^ counterparts. Indeed, these significant differences are not borne out in the simple isotropic Gd^III^-radical exchange coupling values where *J*_1_ = −15.9(2) cm^–1^ for **2-Gd** while *J*_1_ = −20 cm^–1^ for [K(crypt-222)(THF)][(Cp_2_^Me4^Gd(THF))_2_(μ-η^2^:η^2^-N_2_)],^19^ indicating that it must be the anisotropic orbital exchange interactions that differ between the N_2_^3–^ and Bi_2_^3–^ radicals.

While it appears that the use of Bi_2_^3–^ radicals is worse for SMM properties than N_2_^3–^, perhaps this is an over-simplified conclusion. Given we have shown that having a radical perpendicular to the local magnetic anisotropy axes of the Ln^III^ ions is detrimental to the overall magnetic anisotropy, we postulate that it is precisely because of stronger effects induced by the Bi_2_^3–^ radical compared to the N_2_^3–^ radical, that the SMM properties of **2-Ln** are worse than their N_2_^3–^ predecessors. This is compatible with the observation that both Tb^III^ examples show better magnetic properties than Dy^III^, which is opposite to the case of Cp^iPr5^LnI_3_LnCp^iPr5^;^20^ i.e., stronger orbital exchange contributions arise for Dy^III^ than for Tb^III^ owing to larger orbital angular momentum (*L* = 5 vs *L* = 3), which leads to better properties for the co-parallel arrangement in Cp^iPr5^LnI_3_LnCp^iPr5^, where the exchange coupling is supporting the CF anisotropy, and worse properties for the perpendicular arrangement herein where the exchange coupling is working against the CF anisotropy. Clearly, this advocates for more examples of paramagnetic Bi_2_^3–^-bridged complexes to test this hypothesis. Indeed, complexes with N- and Bi-based radical bridges co-parallel with the other anisotropy-generating ligands would be ideal to compare to the present perpendicular class of compounds. Given that unique electronic states can be stabilized in reduced di-lanthanide compounds,^20^ and that di-lanthanide compounds can support unprecedented bridging zintl ions,^36^ perhaps more exotic radical inorganic bridges are possible. Furthermore, as elements from both the top (i.e., N) and bottom (i.e., Bi) of group 5 can support similar chemistry and host analogous electronic structures, this suggests that P-, As-, and Sb-based radical bridges are possible; this would allow unprecedented insights into periodic trends in exchange coupling that were previously unthinkable.

## Conclusions

We have synthesized the first series of dibismuthene Bi_2_^2–^-bridged complexes containing the heavy lanthanide ions gadolinium, terbium, dysprosium, and the rare earth yttrium ion. Treatment with potassium graphite initiates one-electron reduction of the Bi_2_^2–^ complexes to afford four Bi_2_^3–^ radical-bridged compounds. These molecules represent the first Bi_2_^3–^ coordination complexes containing any d- or f-block element. In fact, this constitutes the only second report of a Bi_2_^3–^ radical which differs from the first in that the Bi_2_^3–^ radical anion is side-on ligated to both rare earth ions forming a planar RE_2_(μ-η^2^:η^2^) arrangement. We have studied these molecules with single-crystal X-ray diffraction, UV–vis/NIR spectroscopy, SQUID magnetometry, and multiconfigurational ab initio calculations. Our analysis reveals a π̂_z_^*^ SOMO for the Bi_2_^3–^ radical bridge, engendering strong antiferromagnetic exchange coupling with the paramagnetic metal ions, leading to a ferrimagnetic ground state. The isotropic Ln-radical exchange coupling is −15.9(2) cm^–1^ in **2-Gd**, while the equivalent terms are ca. −19 and −24 cm^–1^ for **2-Tb** and **2-Dy**, respectively. However, the magnetic interactions for the latter two complexes are significantly more complicated owing to nonzero orbital angular momentum and SO coupling. Here, exchange terms of the form *R̂*_α_*Ŝ*_α_*Ô*_*k*_^*q*^ (α ∈ *x,y,z*; *k* ∈ 2,4,6; *q* ∈ −*k*··· + *k*), which represent isotropic spin–spin interactions modulated by anisotropic orbital angular momentum contributions, are important in both compounds. Both **2-Tb** and **2-Dy** are single-molecule magnets; however, their performance is hindered due to exchange interactions which are orthogonal to the intrinsic single-ion magnetic anisotropy of each site. Nonetheless, these complexes constitute the first SMMs containing purely p-block radicals beneath the second row as a mediator of magnetic exchange for any metal. In particular, the demonstration that the heaviest most stable p-block element bismuth can be employed in a radical state to mediate magnetic coupling and engender magnet-like properties paves the way for the generation and study of unprecedented radicals of almost the entirety of the p-block which will have important ramifications for single-molecule magnetism, main group element, rare earth metal and coordination chemistry at large.

## Experimental Section

All experimental procedures are shown in the Supporting Information, including synthesis methods, crystallographic measurements, magnetic measurements, and computational methodology.

## Supporting Information Available

Experimental synthetic details; crystallographic details; molecular structures and bond lengths/angles; UV–vis–NIR spectra; magnetic measurements; and calculation details (PDF)

## Abbreviations

SOMO: singly occupied molecular orbital
SMM: single-molecule magnet
Bi: bismuth
Cp*: pentamethylcyclopentadienyl
Cp^tet^: tetramethylcyclopentadienyl
Cp^Me^: methylcyclopentadienyl
Mes: mesityl
DFT: density functional theory
SA-CASSCF: state-averaged complete active space self-consistent field
CASCI: complete active space configuration interaction
CASPT2: complete active space second-order perturbation theory
MCPDFT: multiconfigurational pair-density-functional theory
SO: spin–orbit
