# Supplementary material for: Taming Super-Reduced Bi23– Radicals with Rare Earth Cations
Source: J Am Chem Soc. 2023 Apr 12;145(16):9152–63. doi: 10.1021/jacs.3c01058 (PMC10141245; doi:10.1021/jacs.3c01058)
Supplement: Supplementary file 1 — ja3c01058_si_001.pdf [file ja3c01058_si_001.pdf]

Supporting Information

for

Taming Super-Reduced Bi_2^{3-} Radicals with Rare Earth Cations

Peng Zhang[1], Rizwan Nabi,[2] Jakob K. Staab,[2] Nicholas F. Chilton,[2]* Selvan Demir[1]*

[1] Department of Chemistry, Michigan State University, 578 South Shaw Lane, East Lansing, MI 48824, USA; sdemir@chemistry.msu.edu

[2] Department of Chemistry, The University of Manchester, Oxford Road, Manchester, M13 9PL, UK; nicholas.chilton@manchester.ac.uk

Table of Contents

Experimental Section......S4
Synthesis of $(Cp^*{}_2Gd)_2(\mu\text{-}\eta^2{:}\eta^2\text{-}Bi_2)$, **1-Gd**......S4
Synthesis of $(Cp^*{}_2Dy)_2(\mu\text{-}\eta^2{:}\eta^2\text{-}Bi_2)$, **1-Dy**......S5
Synthesis of $(Cp^*{}_2Tb)_2(\mu\text{-}\eta^2{:}\eta^2\text{-}Bi_2)$, **1-Tb**......S5
Synthesis of $(Cp^*{}_2Y)_2(\mu\text{-}\eta^2{:}\eta^2\text{-}Bi_2)$, **1-Y**......S5
Synthesis of $[K(crypt\text{-}222)][(Cp^*{}_2Gd)_2(\mu\text{-}\eta^2{:}\eta^2\text{-}Bi_2)]\cdot 2THF$, **2-Gd**......S6
Synthesis of $[K(crypt\text{-}222)][(Cp^*{}_2Dy)_2(\mu\text{-}\eta^2{:}\eta^2\text{-}Bi_2)]\cdot 2THF$, **2-Dy**......S6
Synthesis of $[K(crypt\text{-}222)][(Cp^*{}_2Tb)_2(\mu\text{-}\eta^2{:}\eta^2\text{-}Bi_2)]\cdot 2THF$, **2-Tb**......S6
Synthesis of $[K(crypt\text{-}222)][(Cp^*{}_2Y)_2(\mu\text{-}\eta^2{:}\eta^2\text{-}Bi_2)]\cdot 2THF$, **2-Y**......S7
Crystallography......S7
Magnetic measurements......S8
Computational methodology......S8
Figure S1. Structures of two independent molecules in **2-Gd**, **2-Tb**, and **2-Dy**......S10
Figure S2. Molecular reference frame for **1-RE** and **2-RE**......S11
Figure S3. Temperature dependence of the $\chi_M T$ product for polycrystalline **1-Y**......S11
Figure S4. The field (H) dependence of the magnetization (M) for **1-Gd** and **2-Gd**......S12
Figure S5. The field (H) dependence of the magnetization (M) for **1-Tb** and **2-Dy**......S13
Figure S6. The field (H) dependence of the magnetization (M) for **2-Tb** and **2-Dy**......S14
Figure S7. Simulated magnetization curves for **1-Tb** with an Ising spin Hamiltonian......S15
Figure S8. Simulated magnetization curves for **1-Dy** with an Ising spin Hamiltonian......S15
Figure S9. Simulated magnetic susceptibility for **2-Tb**......S16
Figure S10. Simulated magnetic susceptibility for **2-Dy**......S16
Figure S11. Ac magnetic susceptibility data collected for **1-Dy**......S17
Figure S12. Ac magnetic susceptibility data collected for **1-Tb**......S18
Figure S13. Ac magnetic susceptibility data collected for **2-Tb**......S19
Figure S14. Ac magnetic susceptibility data collected for **2-Tb** in dc fields......S20
Figure S15. Ac magnetic susceptibility data collected for **2-Tb** in 1500 Oe......S21
Figure S16. Cole-Cole plots for **2-Dy** in zero applied dc field......S22
Figure S17. Cole-Cole plots for **2-Tb** in 1500 Oe......S22
Figure S18. Relaxation time data for **2-Dy**......S23
Figure S19. Relaxation time data for **2-Tb** in 1500 Oe......S23
Figure S20. IR spectra for **1-RE** and **2-RE**......S24
Figure S21. ^{1}H NMR spectrum for **1-Y** in benzene-d^6......S24
Table S1 Crystal data and structure refinement for **1-Gd**......S25
Table S2 Crystal data and structure refinement for **1-Tb**......S25
Table S3 Crystal data and structure refinement for **1-Dy**......S26
Table S4 Crystal data and structure refinement for **1-Y**......S27
Table S5 Crystal data and structure refinement for **2-Gd**......S28
Table S6 Crystal data and structure refinement for **2-Tb**......S29
Table S7 Crystal data and structure refinement for **1-Dy**......S29
Table S8 Crystal data and structure refinement for **1-Y**......S30
Table S9 Natural orbital occupations and energies for Bi_2^{3-} with CASCI-MCPDFT......S32

Table S10 Average (pseudo-natural) orbitals for **1-Y** from SA-CASSCF.......................... S32
Table S11 Average (pseudo-natural) orbitals for **2-Y** from SA-CASSCF.......................... S33
Table S12 Natural orbital occupations and energies for **2-Y**.. S34
Table S13 Exchange coupling constants, *J*, for dinuclear Gd complexes....................... S35
Table S14 Broken-symmetry DFT results on **1-Gd** and **2-Gd**.. S36
Table S15 Cole-Davidson fits of AC data for **2-Dy**.. S36
Table S16 Cole-Davidson fits of AC data for **2-Tb**... S36
Table S17 SA-CASSCF-SO results for Tb1 and Tb2 in **1-Tb**.. S37
Table S18 SA-CASSCF-SO results for Dy1 in **1-Dy**.. S38
Table S19 SA-CASSCF-SO results for Dy2 in **1-Dy**.. S38
Table S20 CF parameters from SA-CASSCF-SO calculations Tb1 and Tb2 in **1-Tb**...... S39
Table S21 CF parameters from SA-CASSCF-SO calculations Dy1 and Dy2 in **1-Dy**...... S40
Table S22 Projected model Hamiltonian parameters from SA-CASSCF-SO calculations for the Tb^{III}-radical pairs in **2-Tb**.. S41
Table S23 Projected model Hamiltonian parameters from SA-CASSCF-CASPT2-SO calculations for the Tb^{III}-radical pairs in **2-Tb**.. S42
Table S24 CF energies and wavefunctions for Tb1 in **2-Tb** based on projected parameters from SA-CASSCF-SO calculations on a Tb^{III}-radical pair... S43
Table S25 CF energies and wavefunctions for Tb1 in **2-Tb** based on projected parameters from SA-CASSCF-CASPT2-SO calculations on a Tb^{III}-radical pair................................... S43
Table S26 Projected model Hamiltonian parameters from SA-CASSCF-SO calculations for the Dy^{III}-radical pair in **2-Dy**.. S44
Table S27 Projected model Hamiltonian parameters from SA-CASSCF-CASPT2-SO calculations for the Dy^{III}-radical pair in **2-Dy**.. S45
Table S28 SA-CASSCF-SO results for the Dy^{III} sites in **2-Dy**.. S47
Table S29 SA-CASSCF-CASPT2-SO results for the Dy^{III} sites in **2-Dy**.......................... S47
References... S48

Experimental Section

The following manipulations below were performed under argon with rigorous exclusion of oxygen and moisture using Schlenk, vacuum line, and glovebox techniques. House nitrogen was purified through a MBraun HP-500-MO-OX gas purifier. 1,4-dioxane, *n*-hexane, and THF were dried over potassium using benzophenone as an indicator and then were distilled before use. Diethyl ether and toluene were passed through alumina columns to remove water after being sparged with dry nitrogen to remove oxygen. Pentamethylcyclopentadiene (Cp*H), allylmagnesium chloride (2.0 M in THF), and anhydrous $RECl_3$ (RE = Gd, Tb, Dy, Y) were purchased from Aldrich and used as received. Triphenylbismuth and 2.2.2.-cryptand (crypt-222) were purchased from Aldrich and recrystallized from hexane. Potassium graphite (KC_8)[1] and $Cp^*{}_2RE(BPh_4)$[2] (RE = Gd, Tb, Dy, Y) were prepared according to literature procedures. Elemental Analyses were performed by Mr. Rui Huang at Michigan State University. IR spectra (Fig. S 20) were recorded with an Agilent Cary 630 ATR spectrometer in an argon-filled glovebox.

NMR spectra (Fig. S21) were recorded on a 500 MHz Agilent DirectDrive2 and calibrated to the residual solvent signal (benzene-d^6: δ_H = 7.16 ppm). Signal multiplicities are abbreviated as: s (singlet), d (doublet), m (multiplet), br (broad). Air-sensitive samples were prepared in a argon-filled glovebox using standard NMR tubes air-tight sealed. NMR solvents (benzene-d^6) were dried by storing over Na/K alloy for several days and filtered prior to use.

UV/vis/NIR spectra were recorded using Perkin-Elmer Lambda 1050 UV-VIS-NIR spectrophotometer in THF solutions with the concentration of **1-Gd** (7.14×10^{-5} mol/L), **1-Tb** (5.08×10^{-5} mol/L), **1-Dy** (5.77×10^{-5} mol/L), **1-Y** (7.74×10^{-5} mol/L), and **2-Gd** (6.92×10^{-5} mol/L), **2-Tb** (3.90×10^{-5} mol/L), **2-Dy** (7.24×10^{-5} mol/L), **2-Y** (6.40×10^{-5} mol/L). The air-sensitive samples were prepared in an argon-filled glovebox and sealed in air-free cuvettes for measurements.

Synthesis of $(Cp^*{}_2Gd)_2(\mu\text{-}\eta^2\text{:}\eta^2\text{-}Bi_2)$, 1-Gd

In an argon- filled glovebox, 0.0200 g (0.045 mmol) of triphenylbismuth ($BiPh_3$) and 0.1358 g of $Cp^*{}_2Gd(BPh_4)$ (0.182 mmol) were weighed into two separate 20 mL scintillation vials and dissolved in 2 and 6 mL of THF, respectively. The $BiPh_3$ solution was added to the $Cp^*{}_2Gd(BPh_4)$ solution, and stirred for 10 min at -30 ˚C. 0.0248 g (0.183 mmol) of KC_8 was added to the reaction mixture and stirred for 60 min at -30 ˚C. Subsequently, the reaction mixture was warmed up to room temperature and stirred for another 10 min. THF was removed in vacuum to afford a dark red solid, which was extracted with toluene, and filtered to produce a dark red solution. After removal of toluene under reduced pressure, the yielded red solid was washed five times with hexane to give a brown powder. Dark red crystals of **1-Gd** suitable for X-ray analysis were grown from concentrated toluene solution at -35 °C in 29% yield (0.0083 g) based on elemental Bi. Anal. Calcd for $Gd_2Bi_2C_{40}H_{60}$: C, 37.73; H, 4.74; N, 0. Found: C,

38.48; H, 5.05; N, 0.06. IR (ATR, cm^{-1}): 2954 (w), 2893 (s), 2850 (s), 2721 (w),1430 (s), 1376 (vs), 1161 (w),1018 (s), 945 (w), 798 (m), 727 (m), 694 (w).

Synthesis of $(Cp^*{}_2Dy)_2(\mu$-η^2:η^2-$Bi_2)$, 1-Dy

In an argon- filled glovebox, 0.0301 g (0.068 mmol) of triphenylbismuth ($BiPh_3$) and 0.2064 g of $Cp^*{}_2Dy(BPh_4)$ (0.274 mmol) were weighed into two separate 20 mL scintillation vials and dissolved in 2 and 8 mL of THF, respectively. The $BiPh_3$ solution was added to the $Cp^*{}_2Dy(BPh_4)$ solution, and stirred for 10 min. 0.0376 g (0.278 mmol) of KC_8 was added to the reaction mixture and stirred for 15 min at 25 °C. THF was removed in vacuum to afford a dark red solid, which was extracted with toluene to afford a dark red solution. After removal of toluene under reduced pressure, the yielded dark red solid was first washed five times with hexane and then with minimum amount of toluene to remove all $Cp^*{}_2DyPh(THF)$ byproduct yielding a brown powder (crude yield: ~35 mg). The entire experiment was repeated twice to obtain approximately 100 mg of product. Dark red crystals of **1-Dy** suitable for X-ray analysis were grown from concentrated toluene solution at -35 °C in 39% yield (0.050 g) based on elemental Bi. Anal. Calcd for $Dy_2Bi_2C_{40}H_{60}$: C, 37.42; H, 4.71; N, 0. Found: C, 37.86; H, 4.72; N, 0.10. IR (ATR, cm^{-1}): 2955 (w), 2894 (s), 2852 (s), 2720 (w),1431 (s), 1375 (vs), 1161 (w),1019 (s), 944 (w), 800 (m), 726 (m), 693 (w). Crystals of $Cp^*{}_2DyPh(THF)$ were obtained from hexane solution at -35 °C and analyzed via X-ray diffraction giving rise to cell parameters that match the reported structure.[3]

Synthesis of $(Cp^*{}_2Tb)_2(\mu$-η^2:η^2-$Bi_2)$, 1-Tb

Following the synthesis route described for **1-Dy**, the reaction of $BiPh_3$ (0.0300 g, 0.068 mmol) in 2 mL of THF, $Cp^*{}_2Tb(BPh_4)$ (0.2041g, 0.272 mmol) in 8 mL of THF, and KC_8 (0.0376 g, 0.278 mmol) afforded after work-up a brown solid. Dark red crystals of **1-Tb** suitable for X-ray analysis were grown from concentrated toluene solution at -35 °C in 37% yield (0.048 g) based on elemental Bi. Anal. Calcd for $Tb_2Bi_2C_{40}H_{60}$: C, 37.63; H, 4.73; N, 0. Found: C, 37.48; H, 4.94; N, 0.08. IR (ATR, cm^{-1}): 2957 (w), 2892 (s), 2851 (s), 2720 (w),1431 (s), 1375 (vs), 1161 (w),1019 (s), 945 (w), 799 (m), 726 (m), 693 (w).

Synthesis of $(Cp^*{}_2Y)_2(\mu$-η^2:η^2-$Bi_2)$, 1-Y

In an argon- filled glovebox, 0.0200 g (0.045 mmol) of triphenylbismuth ($BiPh_3$) and 0.1243 g of $Cp^*{}_2Y(BPh_4)$ (0.183 mmol) were weighed into two separate 20 mL scintillation vials and dissolved in 2 and 6 mL of THF, respectively. The $BiPh_3$ solution was added to the $Cp^*{}_2Y(BPh_4)$ solution, and stirred for 10 min at -30 °C. 0.0248 g (0.183 mmol) of KC_8 was added to the reaction mixture and stirred for 30 min at -30 °C. Subsequently, the reaction mixture was warmed up to room temperature and stirred for another 10 min. THF was removed in vacuum to afford a dark red solid, which was extracted with toluene, and filtered to produce a dark red solution. After removal of toluene under reduced pressure, the

yielded dark red solid was washed five times with hexane to give a brown powder. Dark red crystals of **1-Y** suitable for X-ray analysis were grown from concentrated toluene solution at -35 °C in 30% yield (0.008 g) based on elemental Bi. Anal. Calcd for $Y_2Bi_2C_{40}H_{60}$: C, 42.27; H, 5.32; N, 0. Found: C, 42.75; H, 5.43; N, 0.09. IR (ATR, cm^{-1}): 2956 (w), 2893 (s), 2852 (s), 2719 (w), 1431 (s), 1376 (vs), 1161 (w), 1020 (s), 946 (w), 802 (m), 726 (m), 692 (w). ^1H NMR (Benzene-d^6, 500 MHz): 2.19 (s, CH_3).

Synthesis of [K(crypt-222)][$(Cp^*{}_2Gd)_2(\mu$-η^2:η^2-$Bi_2)$]·2THF, 2-Gd

KC_8 (0.9 mg, 0.065 mmol) was added to 1 mL THF solution of crypt-222 (2.5 mg, 0.0065 mmol) in a Coldwell which was cooled with isopropanol and dry ice. Subsequently, 1 mL of dark red THF solution of **1-Gd** (8.6 mg, 0.0067 mmol) was added to the above solution positioned in the Coldwell. The solution color turned to purple. After 40 min of stirring in the Coldwell, the purple solution was first filtered into a 4 mL vial, then layered with diethyl ether, and subsequently kept in the freezer at -35 °C. Over the course of three days, black crystals of **2-Gd** suitable for X-ray analysis were grown in 53% yield (6.6 mg). Anal. Calcd for $Gd_2Bi_2KN_2O_6C_{58}H_{96}\cdot2(C_4H_8O)$: C, 43.24; H, 6.16; N, 1.53. Found: C, 42.33; H, 5.97; N, 1.56. IR (ATR, cm^{-1}): 2956 (w), 2873 (s), 2850 (s), 2715 (s), 1473 (m), 1439 (m), 1372 (w), 1353 (s), 1294 (m), 1257 (m), 1237 (w), 1171 (w), 1133 (m), 1101 (vs), 1072 (vs), 1025 (m), 950 (s), 931 (s), 830 (m), 820 (m),753 (m).

Synthesis of [K(crypt-222)][$(Cp^*{}_2Dy)_2(\mu$-η^2:η^2-$Bi_2)$]·2THF, 2-Dy

A 1 mL THF solution of crypt-222 (5.0 mg, 0.013 mmol) was added to a stirring 1 mL dark red THF solution of **1-Dy** (19.3 mg, 0.015 mmol). The mixture was stirred for 10 min in a Coldwell which was cooled with isopropanol and dry ice. Subsequently, KC_8 (1.8 mg, 0.013 mmol) was added at once to the reaction mixture, whereby the solution color turned to purple. After 30 min of stirring in the Coldwell, the purple solution was first filtered into a 4 mL vial, then layered with diethyl ether, and subsequently kept in the freezer at -35 °C. Over the course of three days, black crystals of **2-Dy** suitable for X-ray analysis were grown in 31% yield (8.7 mg). Anal. Calcd for $Dy_2Bi_2KN_2O_6C_{58}H_{96}\cdot2(C_4H_8O)$: C, 42.99; H, 6.12; N, 1.51. Found: C, 42.27; H, 6.48; N, 1.56. IR (ATR, cm^{-1}): 2958 (w), 2857 (s), 2827 (s), 2713 (s), 1474 (m), 1439 (m), 1372 (w), 1354 (s), 1295 (m), 1259 (m), 1237 (w), 1172 (w), 1130 (m), 1100 (vs), 1072 (vs), 1022 (m), 950 (s), 931 (s), 830 (m), 820 (m), 752 (m).

Synthesis of [K(crypt-222)][$(Cp^*{}_2Tb)_2(\mu$-η^2:η^2-$Bi_2)$]·2THF, 2-Tb

Following the synthesis procedure described for **2-Dy**, the reaction of **1-Tb** (17.1 mg, 0.013 mmol) in 1 mL of THF, crypt-222 (4.6 mg, 0.012 mmol) in 1 mL of THF, and KC_8 (1.7 mg, 0.012 mmol) afforded a purple solution. Black crystals of **2-Tb** suitable for X-ray analysis were grown from diethyl ether layered THF solution in the freezer at -35 °C in 44% yield (10.9 mg). Anal. Calcd for $Tb_2Bi_2KN_2O_6C_{58}H_{96}\cdot2(C_4H_8O)$: C, 43.16; H, 6.14; N, 1.52. Found: C, 42.53; H,

6.46; N, 1.51. IR (ATR, cm^{-1}): 2954 (w), 2850 (s), 2716 (s), 1475 (m), 1440 (m), 1373 (w), 1353 (s), 1293 (m), 1258 (m), 1236 (w), 1171 (w), 1130 (m), 1100 (vs), 1072 (s), 1025 (m), 950 (s), 931 (s), 830 (m), 820 (m),751 (m).

Synthesis of [K(crypt-222)][$(Cp^*{}_2Y)_2(\mu$-η^2:η^2-$Bi_2)$]·2THF, 2-Y

Following the synthesis procedure described for **2-Gd**, the reaction of **1-Y** (7.6 mg, 0.0067 mmol) in 1 mL of THF, crypt-222 (2.4 mg, 0.0063 mmol) in 1 mL of THF, and KC_8 (0.9 mg, 0.0065 mmol) afforded a purple solution. Black crystals of **2-Y** suitable for X-ray analysis were grown from diethyl ether layered THF solution in the freezer at -35 °C in 57% yield (6.5 mg). Anal. Calcd for $Y_2Bi_2KN_2O_6C_{58}H_{96}\cdot 2(C_4H_8O)$: C, 46.72; H, 6.65; N, 1.65. Found: C, 46.61; H, 6.52; N, 1.71. IR (ATR, cm^{-1}): 2953 (w), 2874 (s), 2851 (s), 2716 (s), 1475 (m), 1440 (m), 1373 (w), 1354 (s), 1294 (m), 1259 (m), 1236 (w), 1171 (w), 1132 (m), 1101 (vs), 1075 (s), 1021 (m), 949 (s), 932 (s), 830 (m), 818 (m),753 (m).

Crystallography

For complexes **1-Dy**, **2-Dy**, and **2-Tb**, data were collected on a Bruker CCD (charge coupled device) based diffractometer using either MoKα or CuKα radiation. The instrument was equipped with an Oxford Cryostream low-temperature apparatus operating at 173 K. Data were measured using omega and phi scans of 1.0° per frame for 30 s. The total number of images was based on results from the program COSMO[4] where redundancy was expected to be 4.0 and completeness of 100% out to 0.83 Å. Cell parameters were retrieved using APEX II software[5] and refined using SAINT on all observed reflections. Data reduction was performed using the SAINT software[6] which corrects for Lp. Scaling and absorption corrections were applied using SADABS[7] multi-scan technique, supplied by George Sheldrick.

For complexes **1-Gd**, **1-Y**, **1-Tb**, **2-Gd**, **2-Y**, data were collected using an XtaLAB Synergy, Dualflex, HyPix diffractometer operating at T = 100 K. Data were measured using scans of 0.5° per frame using Cu Kα or MoKα radiation. The total number of runs and images was based on the strategy calculation from the program CrysAlisPro (Rigaku, V1.171.40.69a, 2020). The diffraction pattern was indexed. The total number of runs and images was based on the strategy calculation from the program CrysAlisPro (Rigaku, V1.171.40.69a, 2020). Data reduction, scaling and absorption corrections were performed using CrysAlisPro[8] (Rigaku, V1.171.40.69a, 2020). Numerical absorption correction was based on Gaussian integration over a multifaceted crystal model. Empirical absorption correction was done using spherical harmonics as implemented in SCALE3 ABSPACK[9] scaling algorithm (spherical harmonics and frame scaling).

Using Olex2,[10] the structures were solved with the ShelXT[11] structure solution program using intrinsic phasing and refined with version ShelXL[12] using least squares minimization. All non-hydrogen atoms are refined anisotropically. Hydrogen atoms were calculated by geometrical methods and refined as a

riding model. The crystals used for the diffraction study showed no decomposition during data collection.

Magnetic measurements
Magnetic susceptibility measurements were carried out using a Quantum Design MPMS3 SQUID magnetometer equipped with a 7 T magnet. Molten eicosane was added to crystalline samples of **1-RE** and **2-RE**, respectively, to immobilize the crystallites and to ensure good thermal contact between sample and the bath. The samples were sealed airtight and transferred to the SQUID magnetometer. Direct current (dc) magnetic measurements were performed with an external magnetic field of 1000 Oe in the temperature range 2-300 K, and the alternating-current (ac) magnetic measurements were measured with a 3.0 Oe ac field oscillating at frequencies from 0.1 to 1000 Hz. The experimental magnetic susceptibility data are corrected for the diamagnetism estimated from Pascal's tables[13] and sample holder calibration. The diamagnetic correction of **2-Y** was performed by subtracting the magnetic moments of blank sample at different temperatures to get accurate values due to the small magnetic moment of the radical.

Computational methodology
All computations were performed using XRD geometry with no optimization. Broken-symmetry DFT calculations were performed using Gaussian 09 rev. D,[14] with the B3LYP functional,[15-17] the 6-31G* basis set (C and H),[18] the 46 core electron Stuttgart-Dresden ECP and double-zeta valence basis set (Bi),[19-21] and the 46 core electron Cundari Stevens Double Zeta ECP and valence basis set (Gd).[22] Calculations were performed for high-spin and broken-symmetry solutions (Table S14), and exchange couplings obtained using the Noodleman method.[23] CASSCF type calculations were performed with OpenMolcas v21.06,[24] using Cholesky decomposition for the two-electron integrals (10^{-8} threshold), second order DKH relativistic decoupling,[25] and basis sets from the ANO-RCC library with VTZP quality (RE), VDZP quality (Bi), VDZ quality (C), and MB quality (H).[26,27] MCPDFT calculations used the tPBE functional,[28] and MSCASPT2 calculations[29] used an imaginary level shift of 0.1 a.u.

To obtain parameters for the Ln^{III}-radical exchange coupling we: i) construct an active space for **2-Ln** containing only the 14 4f orbitals and the Bi_2 $\pi_z{}^*$ SOMO; ii) perform a (2n+1,15) SA-CASSCF calculation for X roots of the highest-spin multiplicity (n = 8, X = 49 and S = 13/2 for **2-Tb**; n = 9, X = 121 and S = 11/2 for **2-Dy**); iii) localise the active orbitals using the Cholesky projected atomic orbital technique;[30] iv) replace one of the Ln^{III} ions with closed-shell Lu^{III} and perform (n+1,8) SA-CASSCF calculations in which the radical SOMO is not allowed to relax, for X roots in the S+1/2 and S-1/2 spin states (X = 7 and S = 3 for **2-Tb**, X = 11 and S = 2.5 for **2-Dy**); v) optionally perform multistate second-order

perturbation theory (MSCASPT2) calculations to account for dynamic correlation in the spin-free states; vi) mix the states with SO coupling; vii) transform the Hamiltonian from the electronic eigenbasis to the $|S, L, m_S, m_L\rangle$ basis and perform a Clebsch-Gordan decomposition to further transform into the $|S_{Ln}, m_{S_{Ln}}, S_{rad}, m_{S_{rad}} L, m_L\rangle$ basis;[31] viii) project the *ab initio* Hamiltonian onto a model Hamiltonian (Equation 2 in the main text) accounting for SO coupling and CF splitting at the Ln^{III} site, and exchange coupling with the radical.[32, 33] Projections of the model Hamiltonian parameters were performed using *molcas_suite*, and model Hamiltonians of the full molecules were calculated using *angmom_suite*; both are available on the PyPI repository.

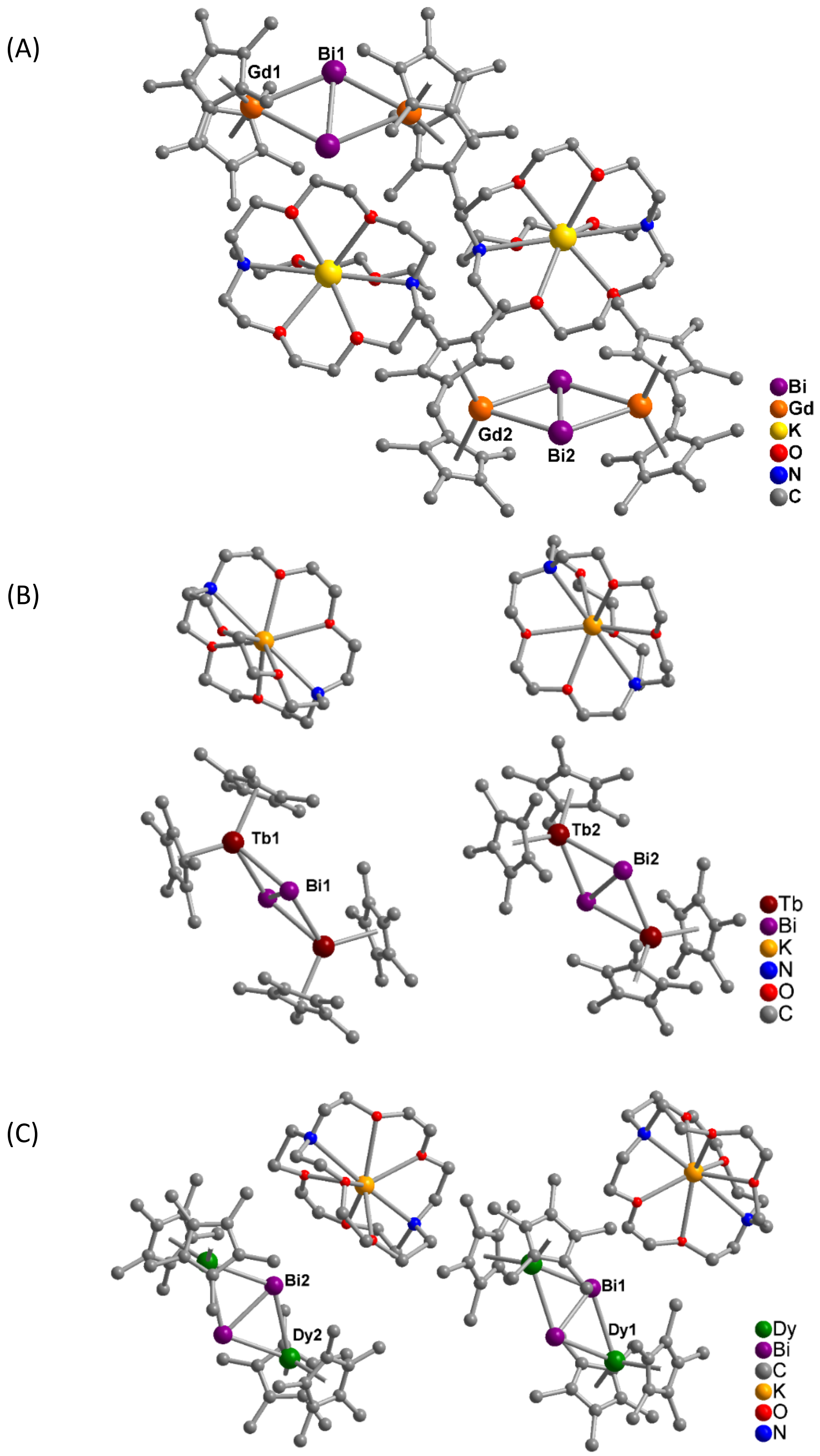

Figure S1. Structures of two independent molecules in **2-Gd** (A), **2-Tb** (B), and **2-Dy** (C) crystallizing in monoclinic I2/a, C2/c, and C2/m, respectively. Orange, dark red, green, yellow, red, blue, and gray spheres represent Gd, Tb, Dy, K, O, N, and C atoms, respectively. H atoms have been omitted for clarity. **2-Y** is isostructural to **2-Dy**.

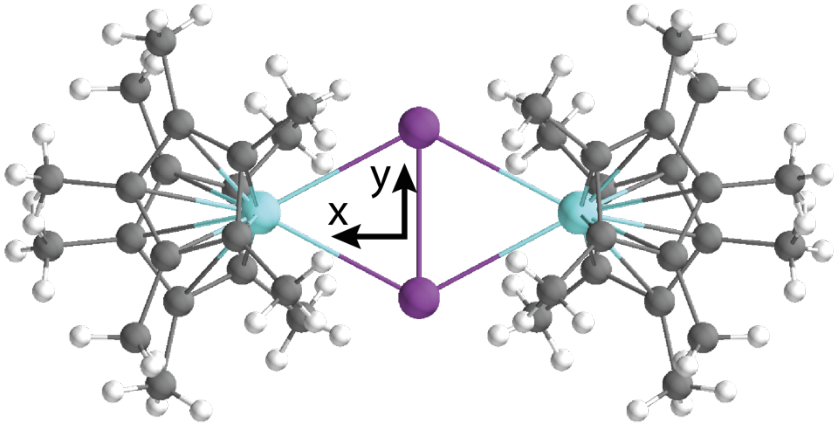

Figure S2. Molecular reference frame for **1-RE** and **2-RE** (structure of **2-Y** shown). Blue: Y; purple: Bi; grey: C; white: H.

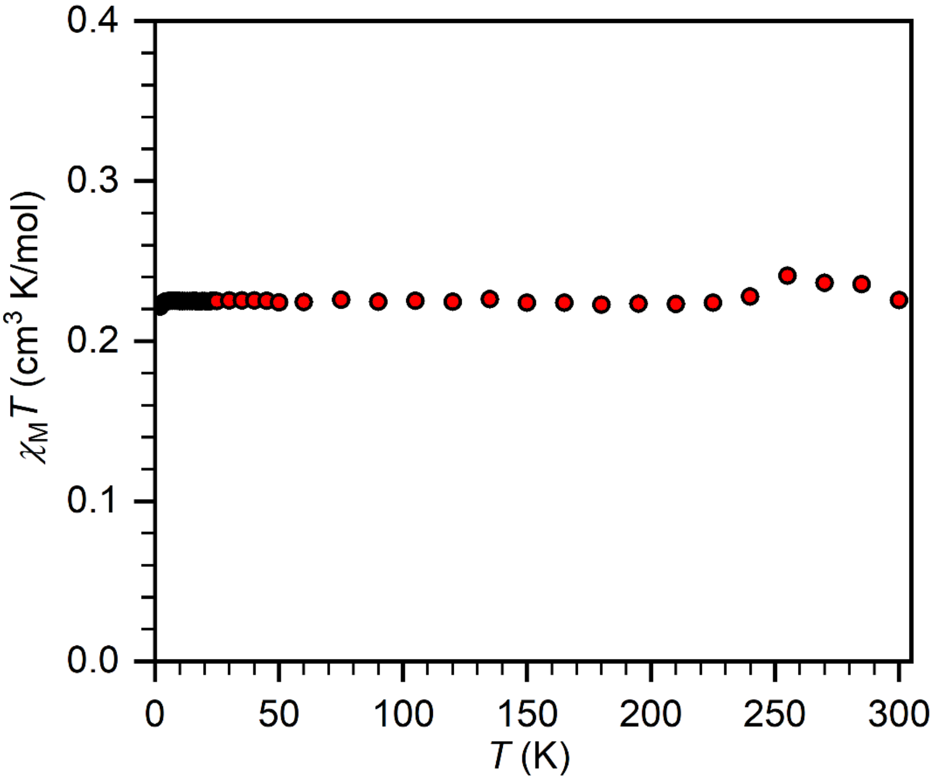

Figure S3. Temperature dependence of the $\chi_M T$ product for polycrystalline **1-Y** under a 1000 Oe applied dc field.

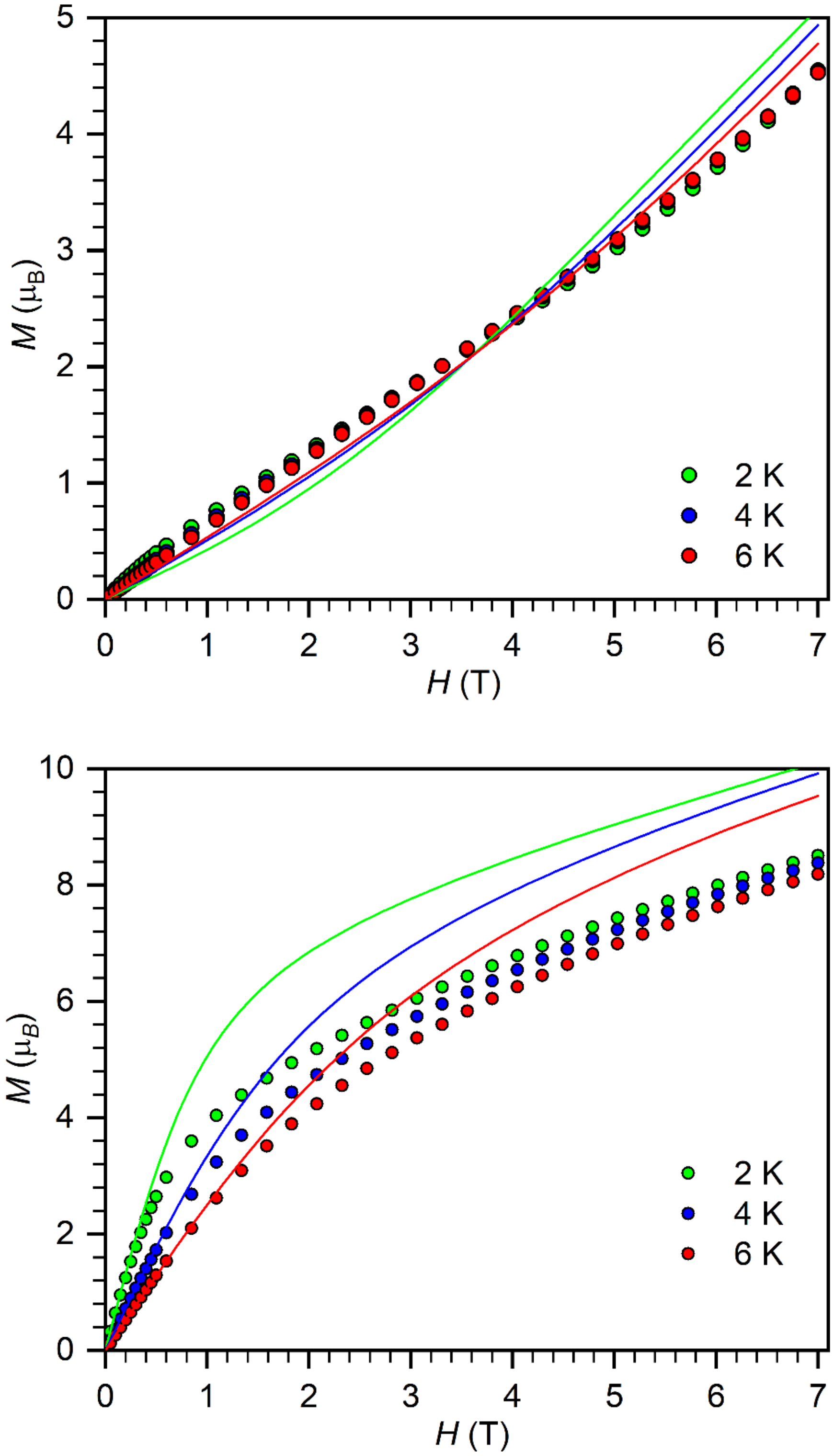

Figure S4. The field (*H*) dependence of the magnetization (*M*) for compounds **1-Gd** and **2-Gd**. (The solid lines correspond to fits in PHI[34])

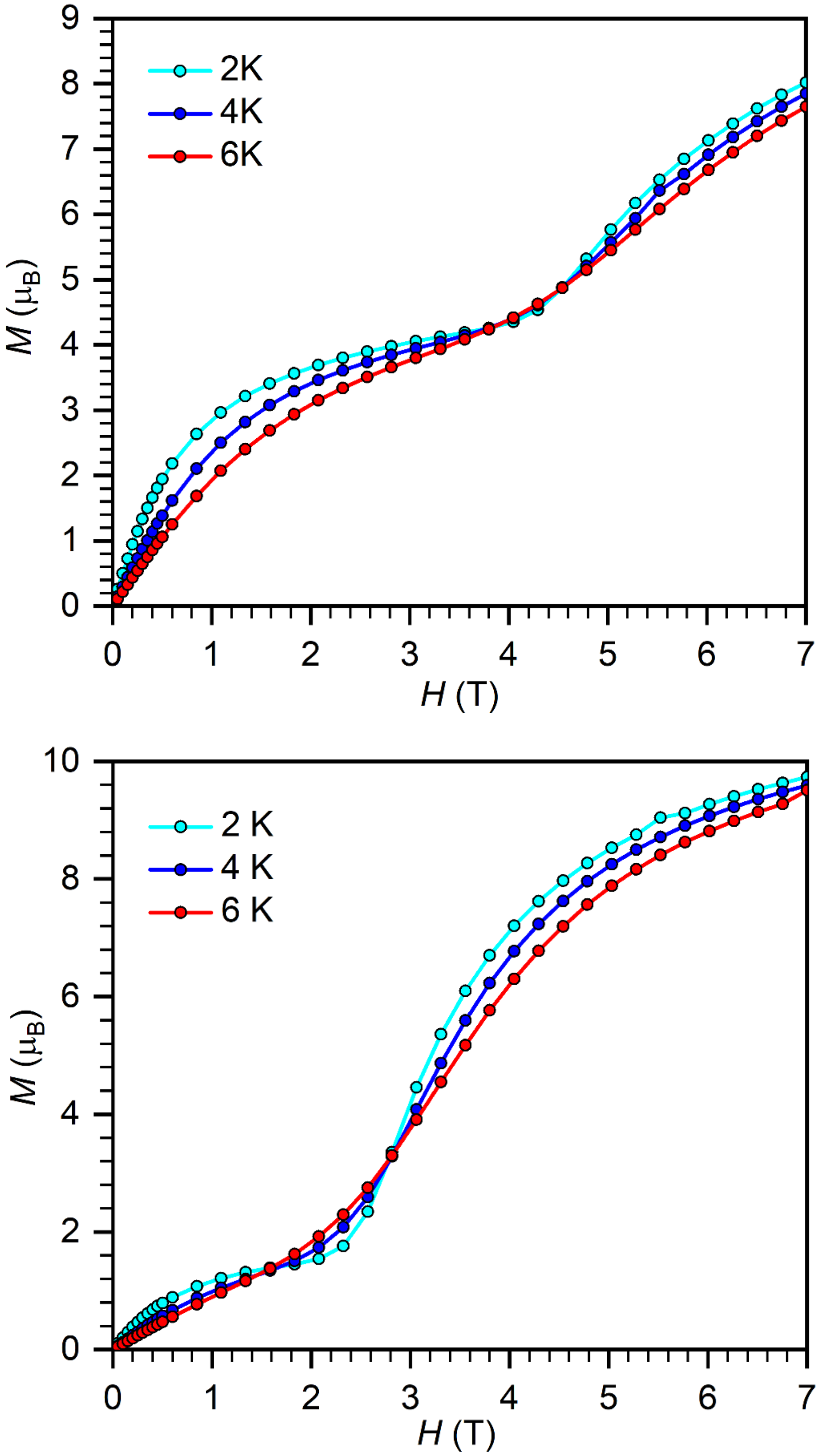

Figure S5. The field (*H*) dependence of the magnetization (*M*) for compounds **1-Tb** (top), **1-Dy** (bottom). Solid lines are guides for the eye.

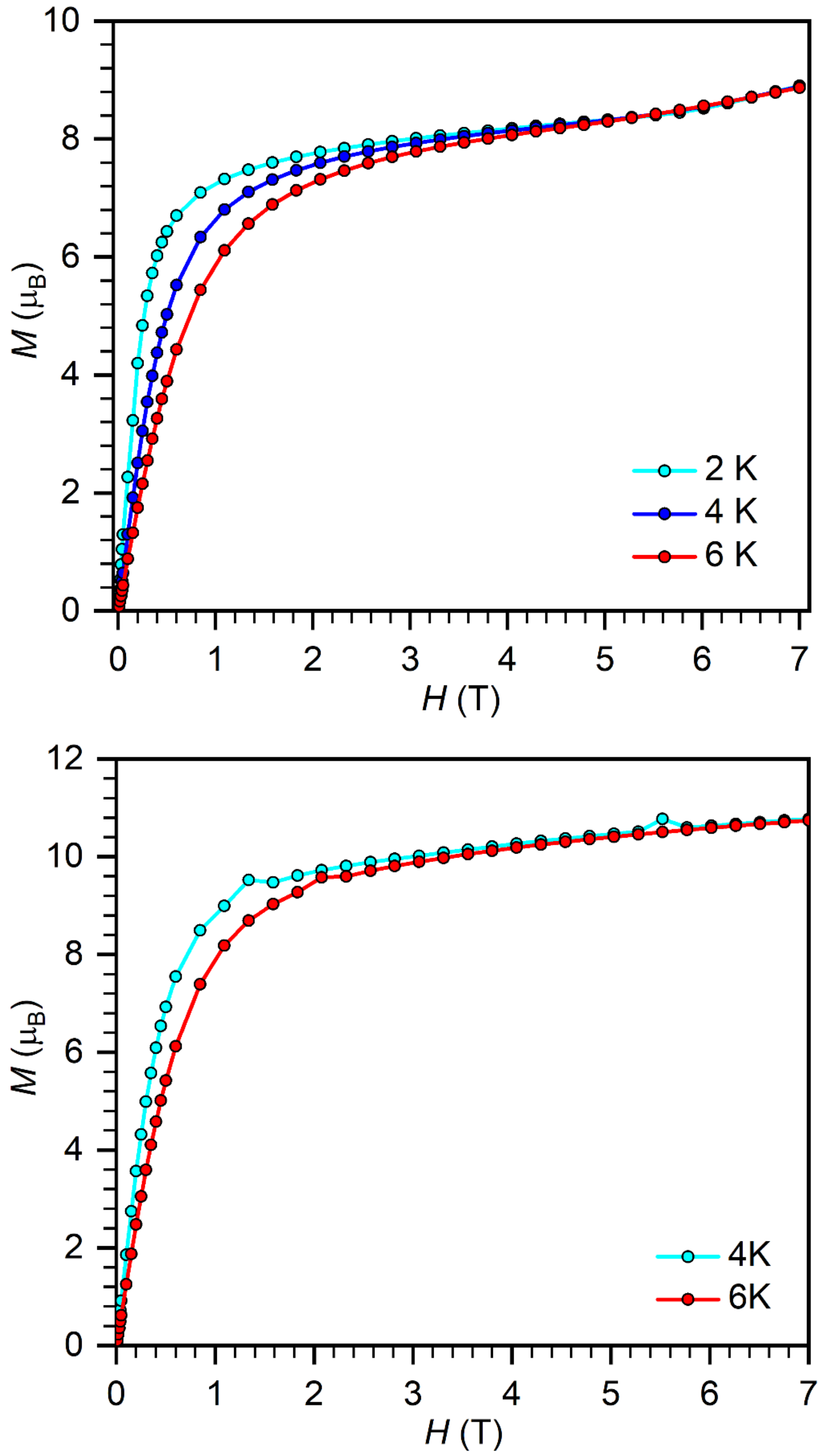

Figure S6. The field (H) dependence of the magnetization (M) for compounds **2-Tb** (top), and **2-Dy** (bottom), respectively. Solid lines are guides for the eye.

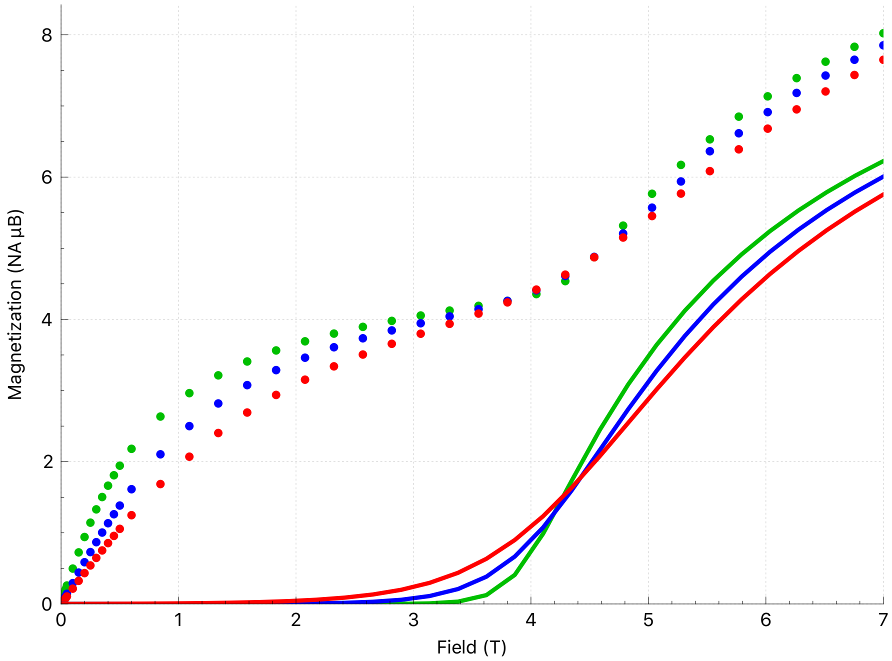

Figure S7. Simulated magnetization curves for **1-Tb** with an Ising spin Hamiltonian (lines) compared to experimental data (points). Green = 2 K, blue = 4 K, red = 6 K.

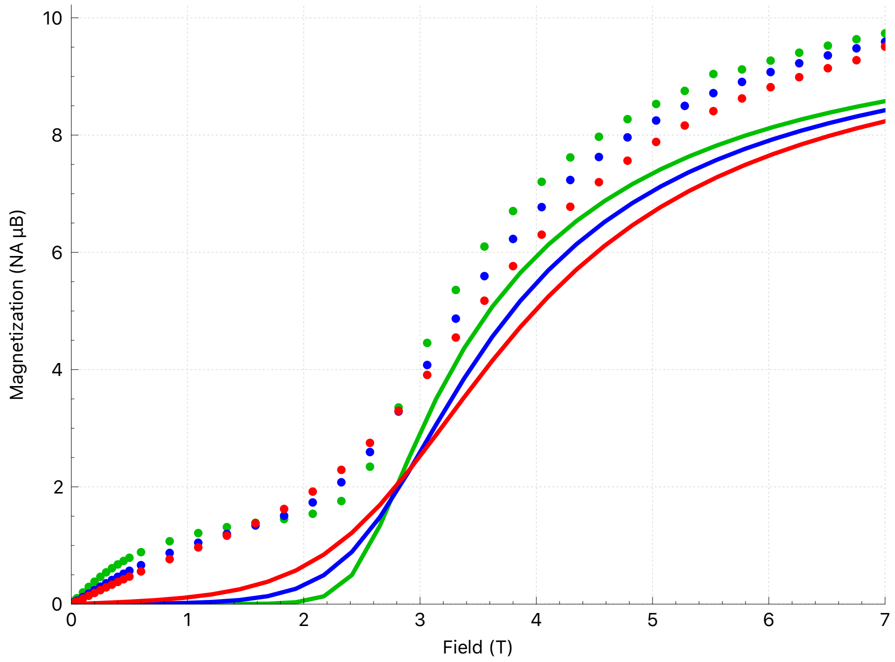

Figure S8. Simulated magnetization curves for **1-Dy** with an Ising spin Hamiltonian (lines) compared to experimental data (points). Green = 2 K, blue = 4 K, red = 6 K.

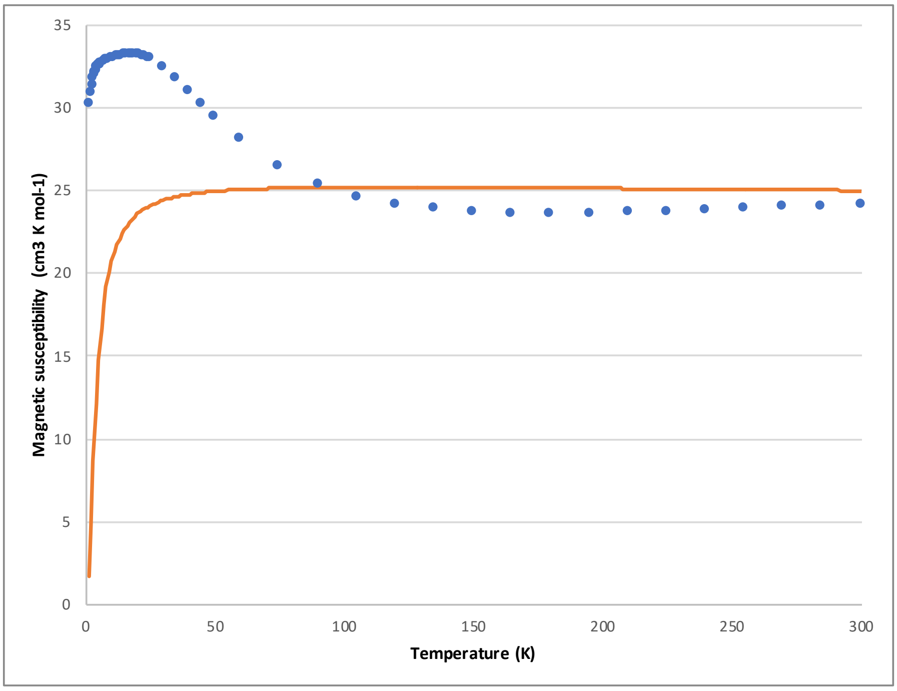

Figure S9. Simulated magnetic susceptibility for **2-Tb** using the projected pair-wise Hamiltonian parameters from CASSCF-SO (line) compared to experimental data (points).

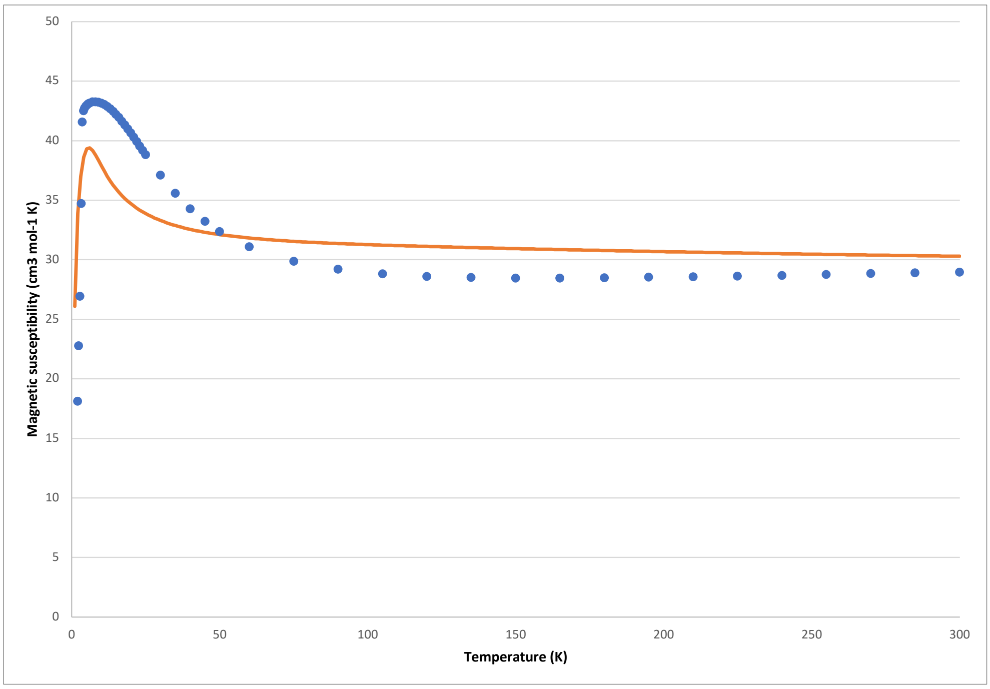

Figure S10. Simulated magnetic susceptibility for **2-Dy** using the projected pair-wise Hamiltonian parameters from CASSCF-SO (line) compared to experimental data (points).

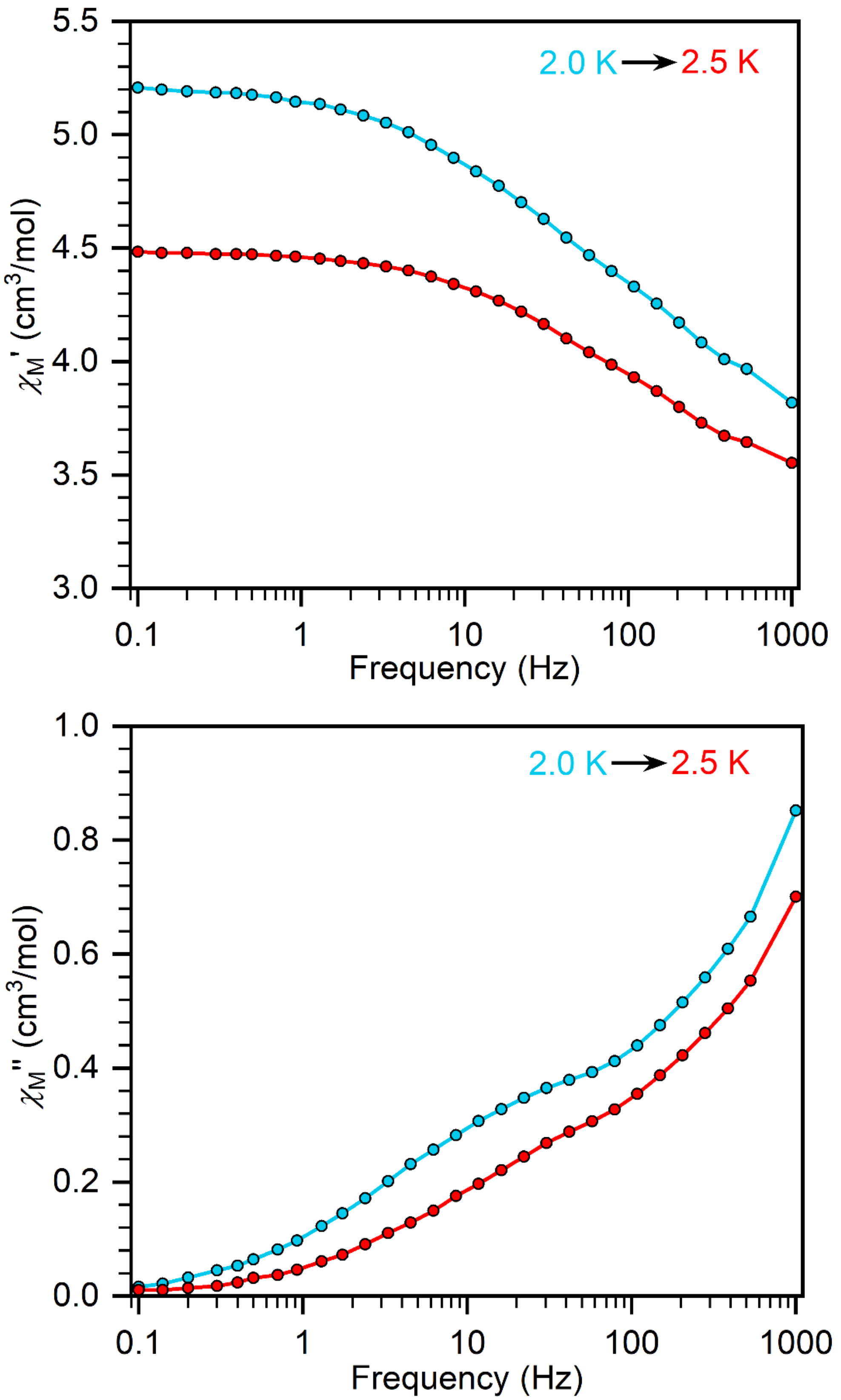

Figure S11. Variable-temperature, variable-frequency in-phase (χ_M', top) and out-of-phase (χ_M'', bottom) ac magnetic susceptibility data collected for **1-Dy** in a zero applied dc field. Solid lines are guides for the eye.

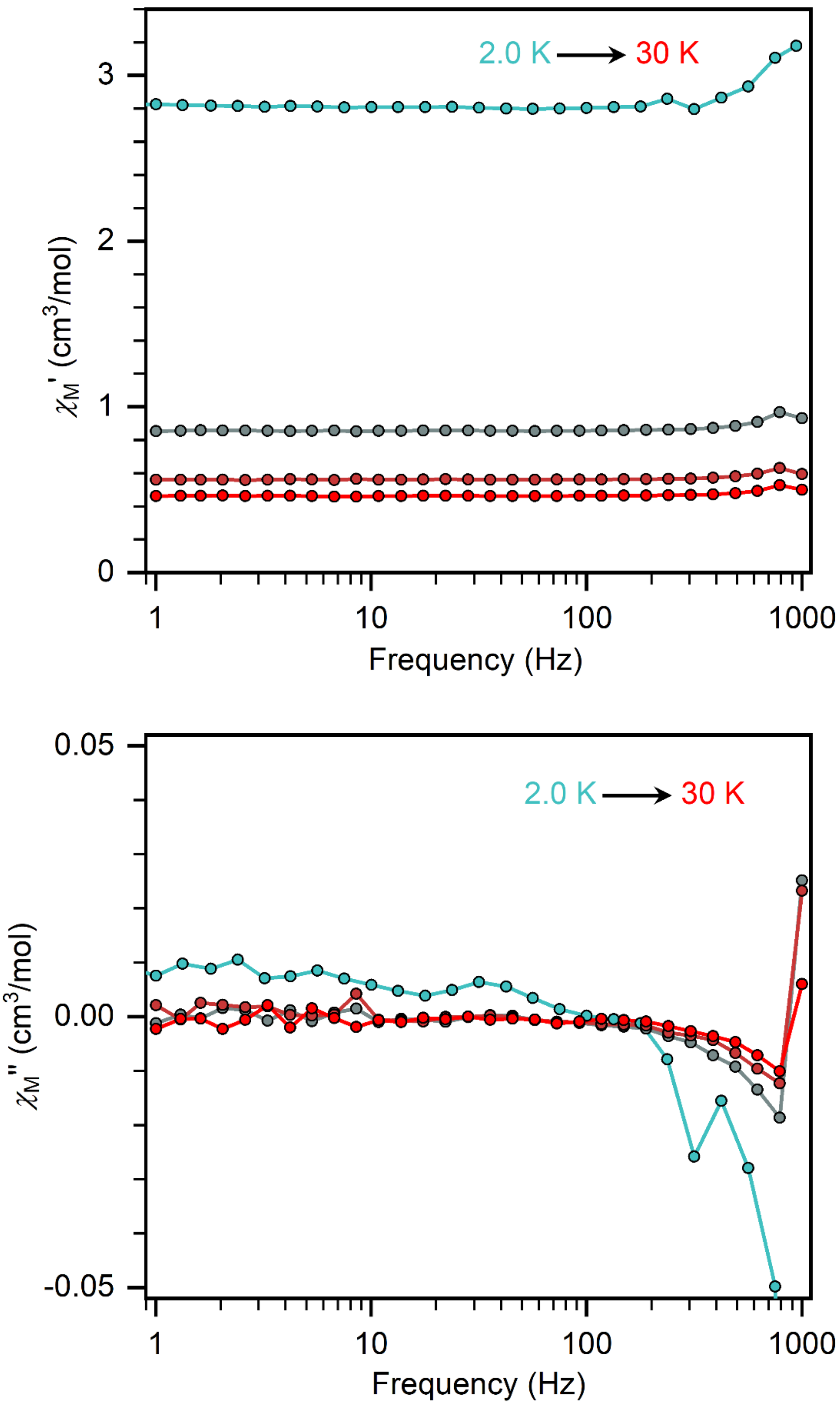

Figure S12. Variable-temperature, variable-frequency in-phase (χ_M', top) and out-of-phase (χ_M'', bottom) ac magnetic susceptibility data collected for **1-Tb** in a zero applied dc field. Solid lines are guides for the eye.

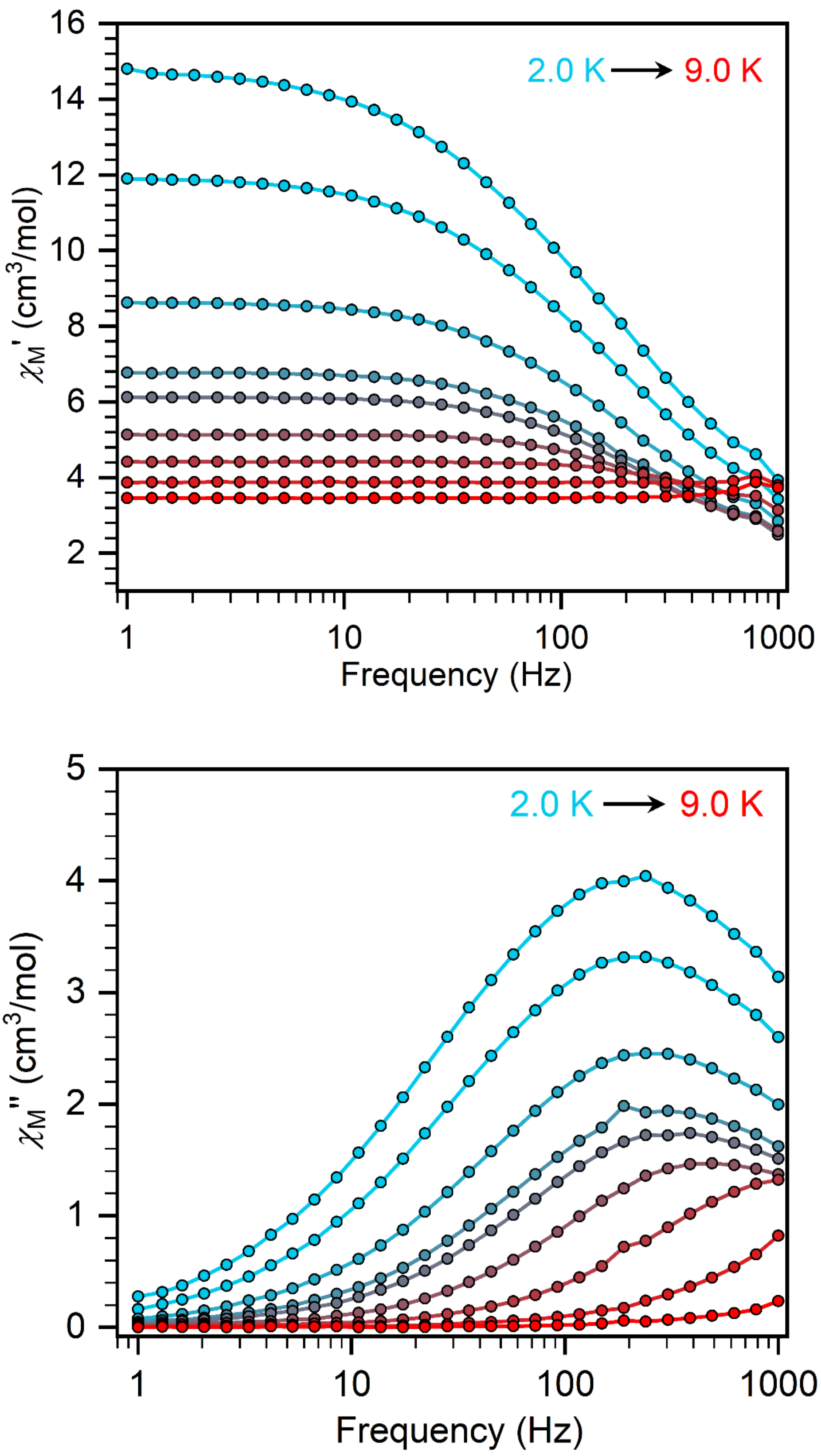

Figure S13. Variable-temperature, variable-frequency in-phase (χ_M') and out-of-phase (χ_M'') ac magnetic susceptibility data collected for **2-Tb** in a zero applied dc field. Solid lines are guides for the eye.

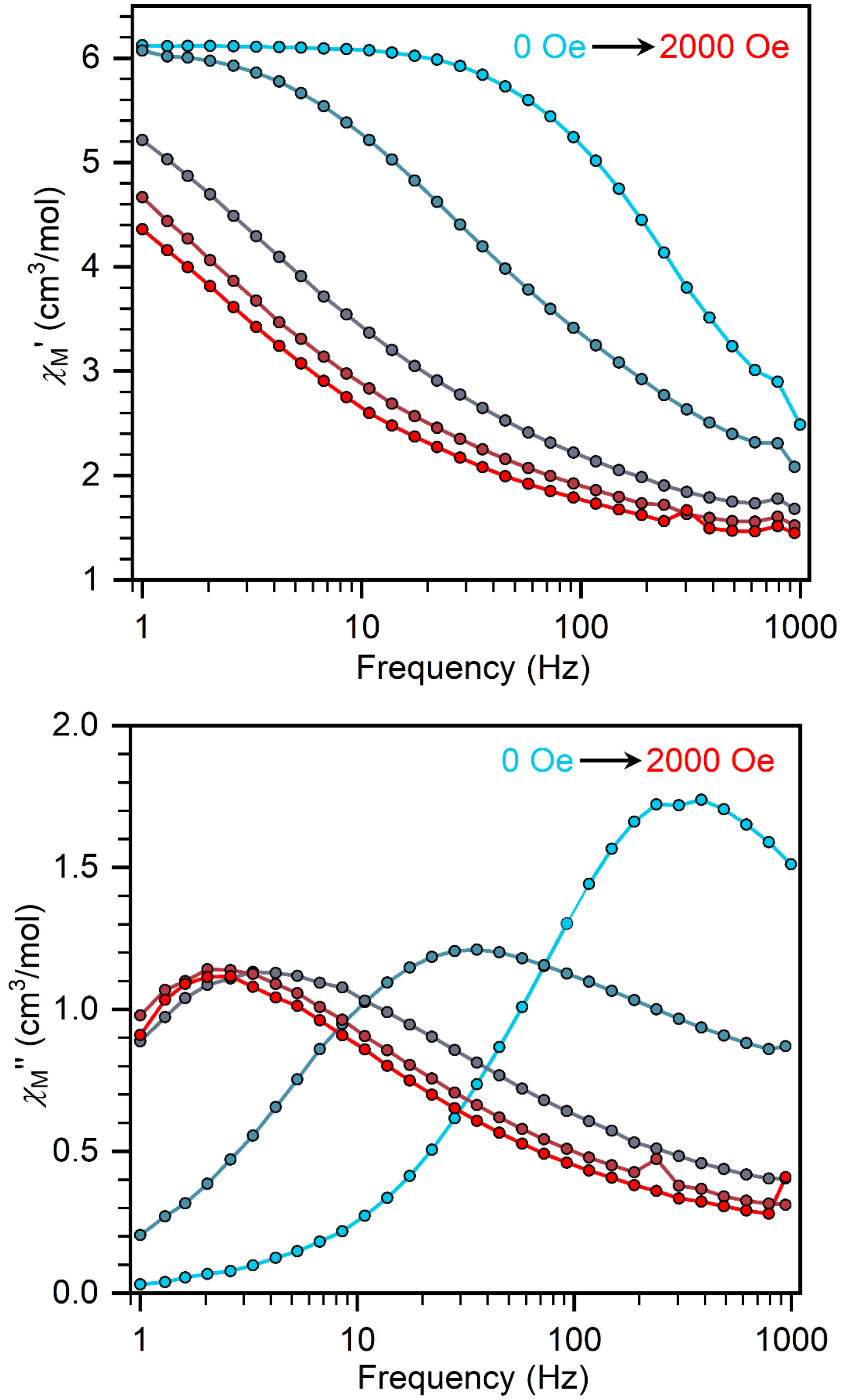

Figure S14. In-phase (χ_M', top) and out-of-phase (χ_M'', bottom) ac magnetic susceptibility collected on **2-Tb** at 5 K under dc fields ranging from 0 to 2000 Oe applied dc field. The change in the low-frequency χ_M'' peak with increasing field appeared to saturate at 1500 Oe.

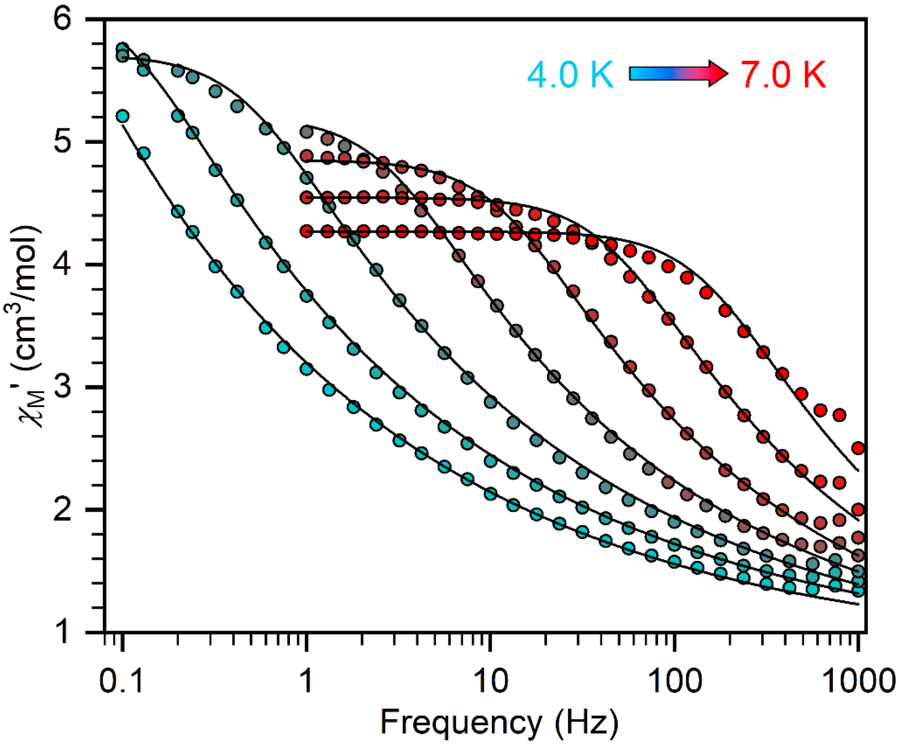

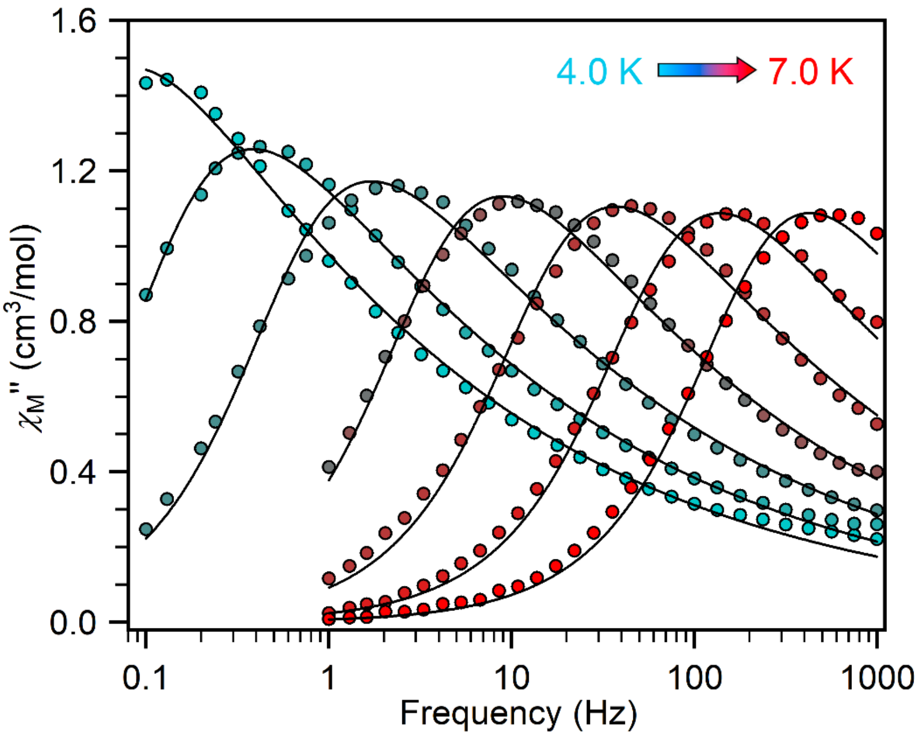

Figure S15. Variable-temperature, variable-frequency in-phase (χ_M', top) and out-of-phase (χ_M'', bottom) ac magnetic susceptibility data collected for **2-Tb** in an applied dc field of 1500 Oe. The solid lines indicate the fits to the Cole-Davidson model.

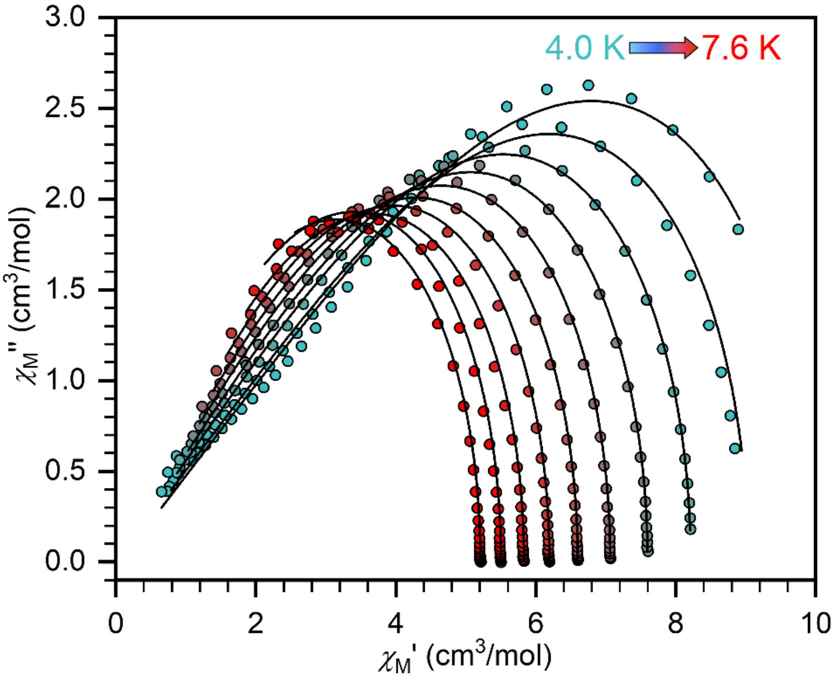

Figure S16 Cole-Cole plots for **2-Dy** in zero applied dc field. The solid lines indicate the fits to the Cole-Davidson model.

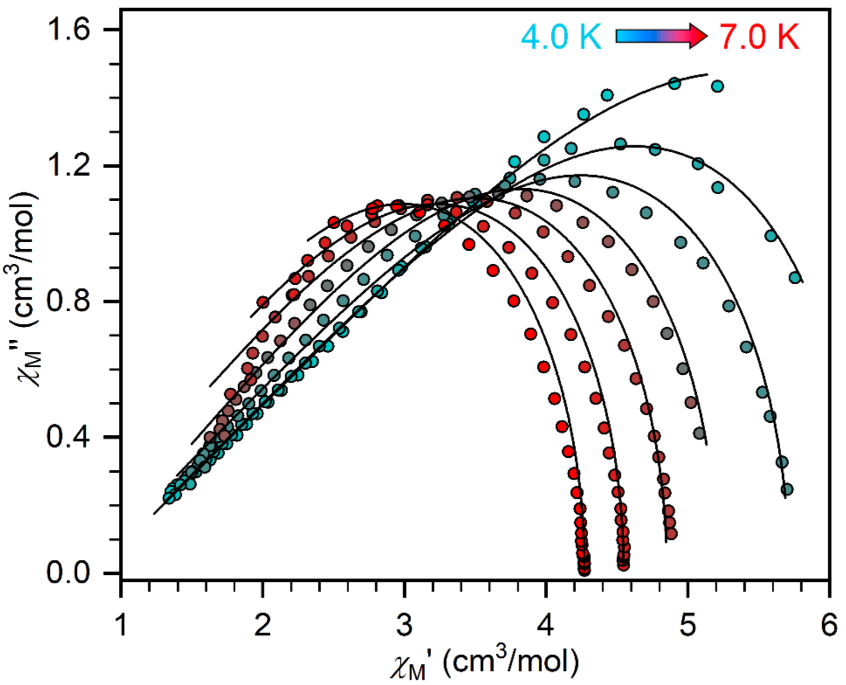

Figure S17 Cole-Cole plots for **2-Tb** in 1500 Oe applied dc field. The solid lines indicate the fits to the Cole-Davidson model.

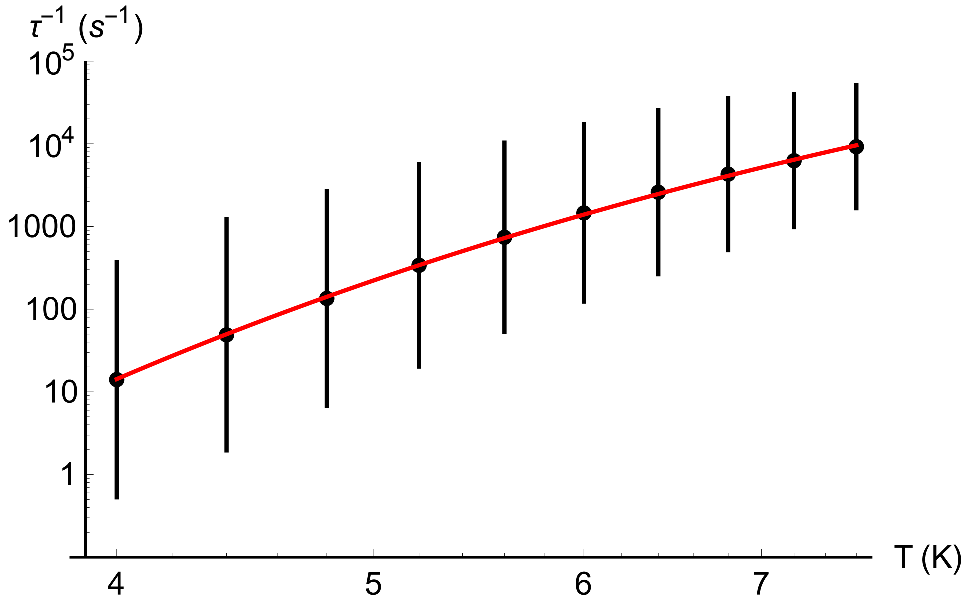

Figure S18. Magnetic relaxation rate for **2-Dy** from ac magnetic susceptibility measurements in a zero applied dc field at temperatures from 4.0 to 7.6 K. A fit to the Orbach expression Log[1/τ] = Log[10^{-A}exp(-U_{eff}/kT)] yielded an effective relaxation barrier of U_{eff} = 38(15) cm^{-1} and a pre-exponential factor of τ_0 = $10^{-7(2)}$ s (red solid line). Error bars indicate one estimated standard deviation using the logarithmic moments of the Cole-Davidson model defined by Zorn.[35]

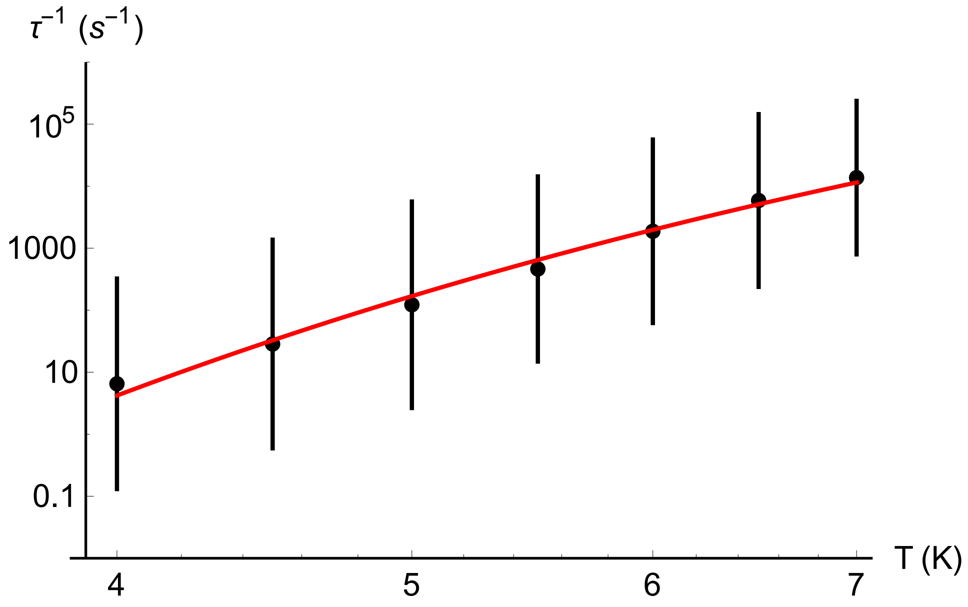

Figure S19. Arrhenius plot of relaxation time data for **2-Tb** from ac magnetic susceptibility measurements under an applied dc field of 1500 Oe at temperatures from 4.0 to 7.0 K. A fit to the Orbach expression Log[1/τ] = Log[10^{-A}exp(-U_{eff}/kT)] yielded an effective relaxation barrier of U_{eff} = 51(26) cm^{-1} and a pre-exponential factor of τ_0 = $10^{-9(3)}$ s (red solid line). Error bars indicate one estimated standard deviation using the logarithmic moments of the Cole-Davidson model defined by Zorn.[35]

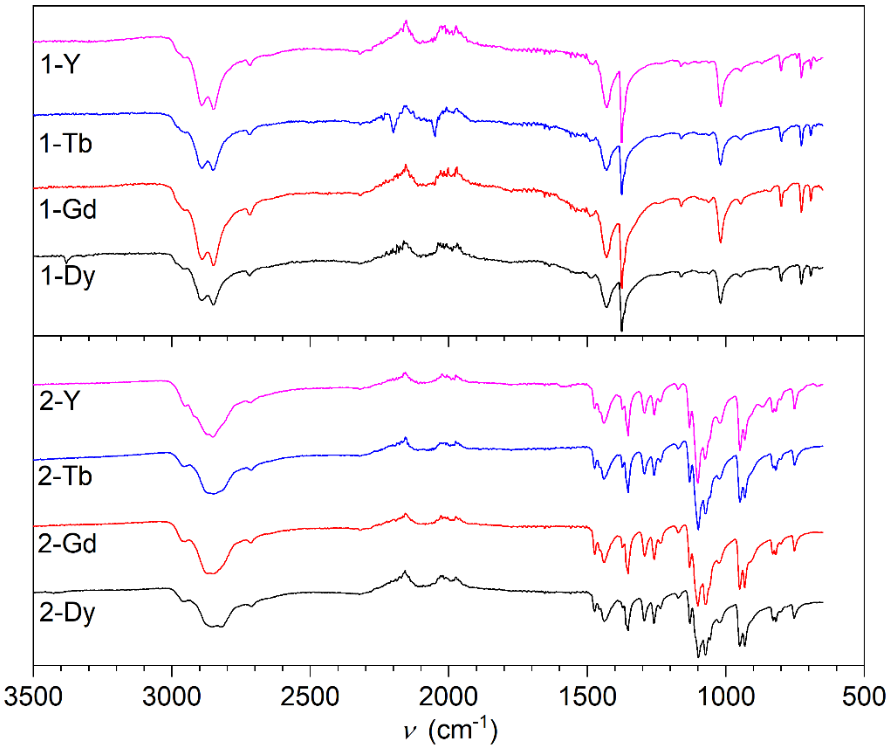

Figure S20 IR spectra for **1-RE** and **2-RE**.

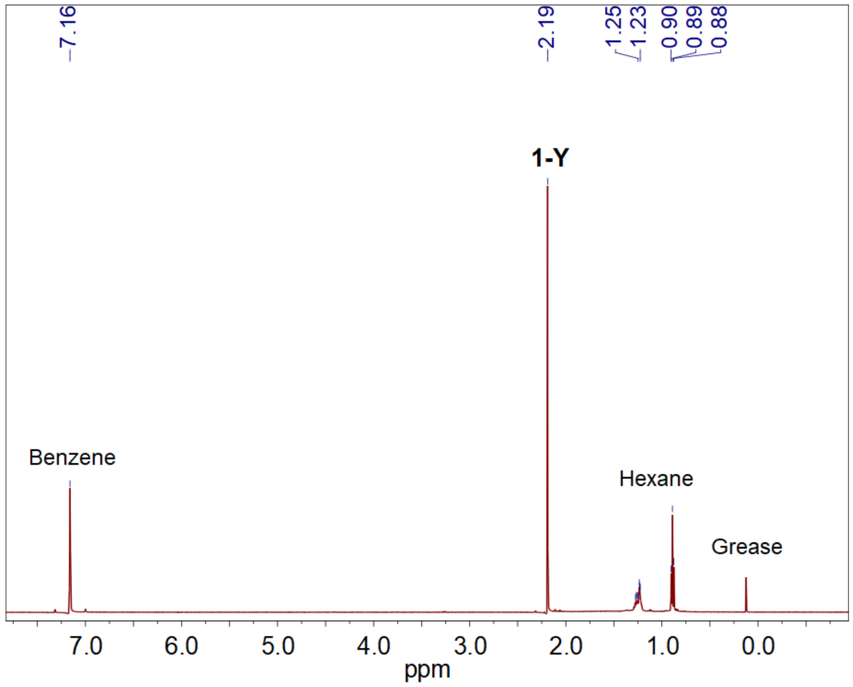

Figure S21 ^{1}H NMR spectrum for **1-Y** in benzene-d^6.

Table S1. Crystal data and structure refinement for 1-Gd.

Identification code	**1-Gd**
CCDC Number	2238559
Empirical formula	$C_{40}H_{60}Bi_2Gd_2$
Formula weight	1273.34
Temperature/K	100(2)
Crystal system	monoclinic
Space group	$P2_1$
a/Å	12.9679(2)
b/Å	10.8873(2)
c/Å	15.2525(2)
α/°	90
β/°	114.185(2)
γ/°	90
Volume/Å^3	1964.42(6)
Z	2
ρ_{calc}/g/cm^3	2.153
μ/mm^{-1}	38.916
F(000)	1188.0
Crystal size/mm^3	0.133 × 0.037 × 0.012
Radiation	CuKα (λ = 1.54184 Å)
2Θ range for data collection/°	6.352 to 154.448
Index ranges	$-16 \le h \le 13, -13 \le k \le 13, -18 \le l \le 19$
Reflections collected	25341
Independent reflections	7943 [R_{int} = 0.0505, R_{sigma} = 0.0493]
Data/restraints/parameters	7943/1/412
Goodness-of-fit on F^2	1.070
Final R indexes [I>=2σ (I)]	R_1 = 0.0423, wR_2 = 0.1124
Final R indexes [all data]	R_1 = 0.0437, wR_2 = 0.1134
Largest diff. peak/hole / e Å^{-3}	1.52/-2.07

Table S2. Crystal data and structure refinement for 1-Tb.

Identification code	**1-Tb**
CCDC Number	2238565
Empirical formula	$C_{40}H_{60}Bi_2Tb_2$
Formula weight	1276.68
Temperature/K	100.00(10)
Crystal system	monoclinic

Space group	$P2_1$
a/Å	8.5667(3)
b/Å	21.3741(7)
c/Å	11.4128(4)
α/°	90
β/°	106.188(4)
γ/°	90
Volume / $Å^3$	2006.89(13)
Z	2
ρ_{calc} /g/cm^3	2.113
μ/mm^{-1}	34.046
F(000)	1192.0
Crystal size/mm^3	0.117 × 0.03 × 0.015
Radiation	Cu Kα (λ = 1.54184 Å)
2Θ range for data collection/°	8.066 to 153.91
Index ranges	-10 ≤ h ≤ 8, -25 ≤ k ≤ 26, -14 ≤ l ≤ 13
Reflections collected	24018
Independent reflections	7607 [R_{int} = 0.0780, R_{sigma} = 0.0678]
Data/restraints/parameters	7607/1/388
Goodness-of-fit on F^2	1.099
Final R indexes [I>=2σ (I)]	R_1 = 0.0560, wR_2 = 0.1433
Final R indexes [all data]	R_1 = 0.0624, wR_2 = 0.1470
Largest diff. peak/hole / e Å^{-3}	1.13/-2.52

Table S3. Crystal data and structure refinement for 1-Dy.

Identification code	**1-Dy**
CCDC Number	2238558
Empirical formula	$C_{40}H_{60}Bi_2Dy_2$
Formula weight	1283.84
Temperature/K	173.15
Crystal system	monoclinic
Space group	$P2_1$
a/Å	8.6008(11)
b/Å	21.463(3)
c/Å	11.4353(15)
α/°	90
β/°	106.263(2)
γ/°	90
Volume/$Å^3$	2026.5(5)

Z	2
ρ_{calc}/g/cm^3	2.104
μ/mm^{-1}	12.326
F(000)	1196.0
Crystal size/mm^3	0.23 × 0.129 × 0.078
Radiation	MoKα (λ = 0.71073 Å)
2Θ range for data collection/°	3.71 to 50.636
Index ranges	-10 ≤ h ≤ 10, -25 ≤ k ≤ 25, -13 ≤ l ≤ 13
Reflections collected	16319
Independent reflections	7254 [R_{int} = 0.0369, R_{sigma} = 0.0579]
Data/restraints/parameters	7254/1/411
Goodness-of-fit on F^2	0.985
Final R indexes [I>=2σ (I)]	R_1 = 0.0338, wR_2 = 0.0652
Final R indexes [all data]	R_1 = 0.0444, wR_2 = 0.0693
Largest diff. peak/hole / e Å^{-3}	1.42/-0.71

Table S4. Crystal data and structure refinement for 1-Y.

Identification code	**1-Y**
CCDC Number	2238566
Empirical formula	$C_{40}H_{60}Bi_2Y_2$
Formula weight	1136.66
Temperature/K	100.00(10)
Crystal system	monoclinic
Space group	P2$_1$
a/Å	8.5648(3)
b/Å	21.4432(7)
c/Å	11.3324(4)
α/°	90
β/°	106.679(4)
γ/°	90
Volume/Å^3	1993.71(13)
Z	2
ρ_{calc}/g/cm^3	1.893
μ/mm^{-1}	20.949
F(000)	1088.0
Crystal size/mm^3	0.096 × 0.048 × 0.046
Radiation	Cu Kα (λ = 1.54184 Å)
2Θ range for data collection/°	8.144 to 133.2

Index ranges	$-10 \le h \le 10$, $-20 \le k \le 25$, $-13 \le l \le 13$
Reflections collected	26199
Independent reflections	6578 [R_{int} = 0.0466, R_{sigma} = 0.0376]
Data/restraints/parameters	6578/1/416
Goodness-of-fit on F^2	1.128
Final R indexes [I>=2σ (I)]	R_1 = 0.0439, wR_2 = 0.1178
Final R indexes [all data]	R_1 = 0.0452, wR_2 = 0.1191
Largest diff. peak/hole / e Å^{-3}	3.10/-4.34

Table S5. Crystal data and structure refinement for 2-Gd.

Identification code	**2-Gd**
CCDC Number	2238568
Empirical formula	$C_{66}H_{112}Bi_2Gd_2KN_2O_8$
Formula weight	1833.13
Temperature/K	100.00(10)
Crystal system	monoclinic
Space group	I2/a
a/Å	20.7554(2)
b/Å	16.12920(10)
c/Å	43.2915(4)
α/°	90
β/°	94.3530(10)
γ/°	90
Volume/Å^3	14450.8(2)
Z	8
ρ_{calc}/g/cm^3	1.685
μ/mm^{-1}	21.966
F(000)	7192.0
Crystal size/mm^3	0.135 × 0.099 × 0.067
Radiation	Cu Kα (λ = 1.54184 Å)
2Θ range for data collection/°	5.85 to 155.624
Index ranges	$-26 \le h \le 21$, $-20 \le k \le 20$, $-46 \le l \le 54$
Reflections collected	85281
Independent reflections	15063 [R_{int} = 0.0496, R_{sigma} = 0.0280]
Data/restraints/parameters	15063/531/1030
Goodness-of-fit on F^2	1.081
Final R indexes [I>=2σ (I)]	R_1 = 0.0489, wR_2 = 0.1366
Final R indexes [all data]	R_1 = 0.0527, wR_2 = 0.1393

Largest diff. peak/hole / e Å^{-3}	2.46/-1.84

Table S6. Crystal data and structure refinement for 2-Tb.

Identification code	**2-Tb**
CCDC Number	2238588
Empirical formula	$C_{66}H_{112}Bi_2KN_2O_8Tb_2$
Formula weight	1836.47
Temperature/K	173.15
Crystal system	monoclinic
Space group	C2/c
a/Å	46.723(4)
b/Å	16.1409(15)
c/Å	20.8558(19)
α/°	90
β/°	111.7300(10)
γ/°	90
Volume/Å^3	14611(2)
Z	8
ρ_{calc}/g/cm^3	1.670
μ/mm^{-1}	6.821
F(000)	7208.0
Crystal size/mm^3	0.105 × 0.089 × 0.062
Radiation	MoKα (λ = 0.71073 Å)
2*Θ* range for data collection/°	2.692 to 50.792
Index ranges	-56 ≤ h ≤ 56, -19 ≤ k ≤ 19, -25 ≤ l ≤ 25
Reflections collected	58218
Independent reflections	13425 [R_{int} = 0.0614, R_{sigma} = 0.0545]
Data/restraints/parameters	13425/478/878
Goodness-of-fit on F^2	1.021
Final R indexes [I>=2σ (I)]	R_1 = 0.0502, wR_2 = 0.1212
Final R indexes [all data]	R_1 = 0.0819, wR_2 = 0.1414
Largest diff. peak/hole / e Å^{-3}	2.77/-1.06

Table S7. Crystal data and structure refinement for 2-Dy.

Identification code	**2-Dy**
CCDC Number	2238567
Empirical formula	$C_{58}H_{98}Bi_2Dy_2KN_2O_6$

Formula weight	1701.44
Temperature/K	173.15
Crystal system	monoclinic
Space group	C2/m
a/Å	20.9384(14)
b/Å	16.1535(11)
c/Å	21.7162(15)
α/°	90
β/°	94.9710(10)
γ/°	90
Volume/Å^3	7317.4(9)
Z	4
ρ_{calc}/g/cm^3	1.544
μ/mm^{-1}	6.910
F(000)	3300.0
Crystal size/mm^3	0.149 × 0.105 × 0.078
Radiation	MoKα (λ = 0.71073 Å)
2Θ range for data collection/°	3.188 to 50.994
Index ranges	-25 ≤ h ≤ 25, -19 ≤ k ≤ 19, -26 ≤ l ≤ 26
Reflections collected	13329
Independent reflections	7006 [R_{int} = 0.0601, R_{sigma} = 0.0941]
Data/restraints/parameters	7006/799/536
Goodness-of-fit on F^2	1.085
Final R indexes [I>=2σ (I)]	R_1 = 0.0737, wR_2 = 0.1884
Final R indexes [all data]	R_1 = 0.1254, wR_2 = 0.2283
Largest diff. peak/hole / e Å^{-3}	3.35/-2.04

Table S8. Crystal data and structure refinement for 2-Y.

Identification code	**2-Y**
CCDC Number	2238589
Empirical formula	$C_{58}H_{98}Bi_2KN_2O_6Y_2$
Formula weight	1552.24
Temperature/K	100.01(10)
Crystal system	monoclinic
Space group	C2/m
a/Å	20.7102(5)
b/Å	16.0940(4)
c/Å	21.6261(6)
α/°	90

β/°	94.997(2)
γ/°	90
Volume/Å^3	7180.8(3)
Z	4
ρ_{calc}/g/cm^3	1.436
μ/mm^{-1}	6.585
F(000)	3076.0
Crystal size/mm^3	0.121 × 0.055 × 0.035
Radiation	Mo Kα (λ = 0.71073 Å)
2Θ range for data collection/°	4.524 to 61.922
Index ranges	-25 ≤ h ≤ 29, -18 ≤ k ≤ 23, -24 ≤ l ≤ 28
Reflections collected	34339
Independent reflections	9345 [R_{int} = 0.0512, R_{sigma} = 0.0556]
Data/restraints/parameters	9345/1624/777
Goodness-of-fit on F^2	1.022
Final R indexes [I>=2σ (I)]	R_1 = 0.0496, wR_2 = 0.0914
Final R indexes [all data]	R_1 = 0.0725, wR_2 = 0.0980
Largest diff. peak/hole / e Å^{-3}	2.90/-2.87

Table S9. Natural orbital occupations and energies for the seven doublet roots of Bi_2^{3-}, computed with CASCI-MCPDFT.

**Orbital/Root**	**1+2**	**3+4**	**5**	**6+7**
$Bi_2(\sigma)$	1.93	1.88	1.02	1.98
$Bi_2(\pi)$	3.96	3.1	3.98	3.8
$Bi_2(\pi^*)$	3.03	3.9	3.96	2.2
$Bi_2(\sigma^*)$	0.07	0.13	0.04	1.02
MCPDFT Energy (cm^{-1})	0	11,999	16,557	16,623

Table S10. Average (pseudo-natural) orbitals for **1-Y** from SA-CASSCF with 12 singlet roots. Isosurfaces plotted at 0.05 and 0.02 a.u.

**Orbital**	**Isosurface**	**Occupation**	**Designation**
269	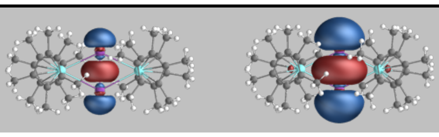	1.74	$Bi_2(\sigma)$
270	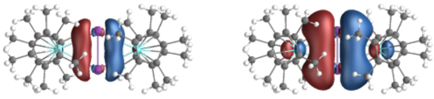	1.83	$Bi_2(\pi_x)$
271	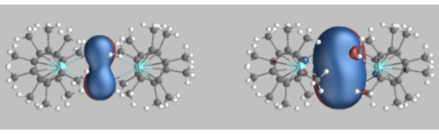	1.53	$Bi_2(\pi_z)$
272	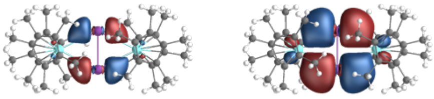	1.45	$Bi_2(\pi_x^*)$
273	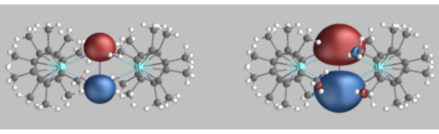	1.05	$Bi_2(\pi_z^*)$
274	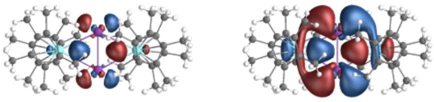	0.02	Bi(6d)/Y(4d)
275	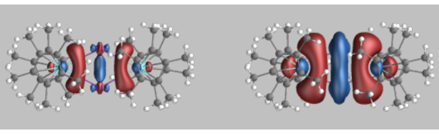	0.02	Bi(6d)/Y(4d)
276	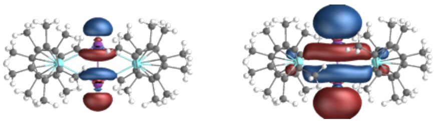	0.34	$Bi_2(\sigma^*)$
277	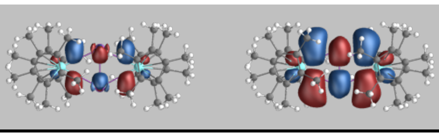	0.01	Bi(6d)/Y(4d)

Table S11. Average (pseudo-natural) orbitals for **2-Y** from SA-CASSCF with 16 doublet roots. Isosurfaces plotted at 0.05 and 0.02 a.u.

Orbital	Isosurface	Occupation	Designation
269	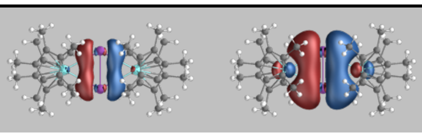	1.90	Bi2(π_x)
270	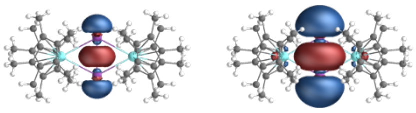	1.88	Bi2(σ)
271	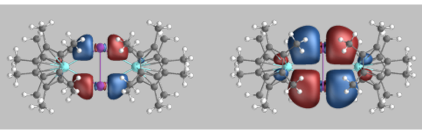	1.64	Bi2(π_x^*)
272	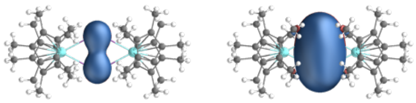	1.69	Bi2(π_z)
273	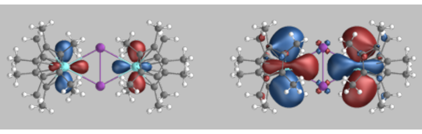	0.26	Y($4d_{x^2-y^2}$)
274	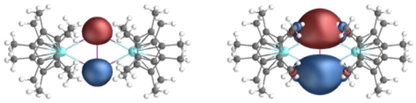	1.12	Bi2(π_z^*)
275	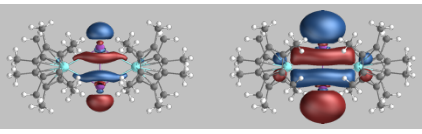	0.32	Bi2(σ^*)
276	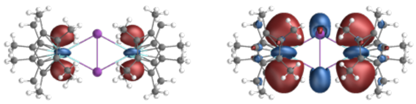	0.20	Y($4d_{y^2}$)

Table S12. Natural orbital occupations and energies for the 16 doublet roots of **2-Y**, computed with SA-CASSCF-MCPDFT.

**Orbital/Root**	**1**	**2**	**3**	**4**	**5**	**6**	**7**	**8**
$Bi_2(\pi_x)$	1.99	2.00	1.99	1.96	1.99	1.99	1.99	1.99
$Bi_2(\sigma)$	1.93	1.98	1.88	1.93	1.94	1.92	1.47	1.54
$Bi_2(\pi_x^*)$	1.99	1.99	1.99	1.03	1.97	1.97	1.99	1.99
$Bi_2(\pi_z)$	1.96	1.78	1.11	2.00	1.78	1.78	1.54	1.46
$Y(4d_{x^2-y^2})$	0.01	0.01	0.01	0.01	0.97	0.04	0.01	0.01
$Bi_2(\pi_z^*)$	1.03	0.23	1.86	1.99	0.23	0.24	1.51	1.42
$Bi_2(\sigma^*)$	0.07	1.02	0.15	0.08	0.09	0.10	0.47	0.59
$Y(4d_{y^2})$	0.01	0.01	0.01	0.01	0.04	0.95	0.03	0.01
MCPDFT Energy (cm^{-1})	0	10,170	13,234	15,640	8,447	9,293	19,911	20,042
**Orbital/Root**	**9**	**10**	**11**	**12**	**13**	**14**	**15**	**16**
$Bi_2(\pi_x)$	1.99	1.82	1.93	1.98	1.88	1.16	1.89	1.90
$Bi_2(\sigma)$	1.94	1.97	1.93	1.93	1.93	1.83	1.91	1.93
$Bi_2(\pi_x^*)$	1.96	1.17	1.11	1.92	1.11	1.83	1.14	1.08
$Bi_2(\pi_z)$	1.02	1.9	1.87	1.08	1.91	1.99	1.92	1.91
$Y(4d_{x^2-y^2})$	0.96	0.01	0.92	0.10	0.05	0.01	0.05	0.98
$Bi_2(\pi_z^*)$	1.00	1.17	1.07	1.10	1.23	1.98	1.08	1.09
$Bi_2(\sigma^*)$	0.08	0.93	0.08	0.10	0.12	0.20	0.94	0.08
$Y(4d_{y^2})$	0.05	0.03	0.09	0.80	0.78	0.01	0.06	0.03
MCPDFT Energy (cm^{-1})	17,086	22,903	16,620	18,357	18,040	24,794	24,794	17,800

Table S13. Exchange coupling constants, *J*, for dinuclear Gd complexes with various diamagnetic bridging ligands.

Complexes	Bridges	*J* (cm^{-1})	References
**[Gd(salicylato)$_3$(H_2O)]$_2$**	R-COO^-	0.025	a
**[Gd(AcO)$_3$(H_2O)$_2$]$_2$**	AcO^-	0.03	b
**[Gd(AmPh)]$_2$**	Ph-O^-	-0.045	c
**[Gd(Hsabhea)(NO_3)]$_2$**	Ph-O^-	-0.099	d
**[Gd_2(valdien)$_2$(NO_3)$_2$]**	Ph-O^-	-0.089	e
**[Cp_2Gd(2-NH-4,6-Me2pm)]$_2$**	R-NH^-	-0.076	f
**[Zn_2(valpn)$_2$$Gd_2$($N_3$)$_6$]**	N_3^-	-0.03	g
**([(Me_3Si)$_2$N]$_2$(THF)Gd)$_2$(*μ*-η^2:η^2-N_2)**	N_2^{2-}	-0.49	h
**[Dy(Cy_2N)$_2$(μ-Cl)(THF)]$_2$**	Cl^-	-0.037	i
**[Cp'$_2$Gd(*μ*-SSiPh$_3$)]$_2$**	R-S^-	-0.105	j
**Gd_2(COT")$_3$**	COT^{2-}	-0.224	k
**[(NN^{TBS})Gd]$_2$(*μ*-biphenyl)[K(18-crown-6)]$_2$**	$Biphenyl^{4-}$	-0.664	l
**[KGd_2(C_7H_7)(N($SiMe_3$)$_2$)$_4$]**	$C_7H_7^{3-}$	-0.134	m

(a) Costes, J.-P.; Clemente-Juan, J. M.; Dahan, F.; Nicodème, F.; Verelst, M. *Angew. Chem. Int. Ed.* **2002**, *41*, 323; (b) Hatscher, S. T.; Urland, W. *Angew. Chem. Int. Ed.* **2003**, *42*, 2862; (c) Liu, S.; Gelmini, L.; Rettig, S. J.; Thompson, R. C.; Orvig, C. *J. Am. Chem. Soc.* **1992**, *114*, 6081. (**AmPh** = tris(((2-hydroxybenzyl)amino) ethyl)amine); (d) Plass, W.; Fries, G. *Z. Anorg. Allg. Chem.* **1997**, *623*, 1205. (**Hsabhea** = N-salicylidene-2-(bis(2-hydroxyethyl) amino)ethylamine); (e) Long, J.; Habib, F.; Lin, P.-H.; Korobkov, I.; Enright, G.; Ungur, L.; Wernsdorfer, W.; Chibotaru, L. F.; Murugesu, M. *J. Am. Chem. Soc.* **2011**, *133*, 5319. (**valdien** = N1,N3-bis(3-methoxysalicylidene) diethylenetriamine); (f) Layfield, R. A.; Bashall, A.; McPartlin, M.; Rawson, J. M.; Wright, D. S. *Dalton Trans.* **2006**, 1660. (**2-NH-4,6-Me2pm** = 2-amino-4,6-dimethylpyrimidine); (g) Huang, X.-C.; Zhou, C.; Wei, H.-Y.; Wang, X.-Y. *Inorg. Chem.* **2013**, *52*, 7314. (**valpn** = 1,3-propanediylbis(2-iminomethylene-6-methoxyphenol)); (h) Rinehart, J. D.; Fang, M.; Evans, W. J.; Long, J. R. *Nat. Chem.* **2011**, *3*, 538; (i) Han, T.; Ding, Y.-S.; Li, Z.-H.; Yu, K.-X.; Zhai, Y.-Q.; Chilton, N. F.; Zheng, Y.-Z. *Chem. Commun.* **2019**, *55*, 7930. (**Cy_2N** = dicyclohexylamine); (j) Tuna, F.; Smith, C. A.; Bodensteiner, M.; Ungur, L.; Chibotaru, L. F.; McInnes, E. J. L.; Winpenny, R. E. P.; Collison, D.; Layfield, R. A. *Angew. Chem. Int. Ed.* **2012**, *51*, 6976; (k) Le Roy, J. J.; Jeletic, M.; Gorelsky, S. I.; Korobkov, I.; Ungur, L.; Chibotaru, L. F.; Murugesu, M. *J. Am. Chem. Soc.* **2013**, *135*, 3502; (l) Huang, W.; Le Roy, J. J.; Khan, S. I.; Ungur, L.; Murugesu, M.; Diaconescu, P. L. *Inorg. Chem.* **2015**, *54*, 2374. (**NN^{TBS}** = 1,1'-fc(NSi^{t}BuMe$_2$)$_2$); (m) Harriman, K. L. M.; Le Roy, J. J.; Ungur, L.; Holmberg, R. J.; Korobkov, I.; Murugesu, M. *Chem. Sci.* **2017**, *8*, 231.

Table S14. Broken-symmetry DFT results on **1-Gd** and **2-Gd** using the B3LYP functional (see Methods for details).

**1-Gd**		
**State/Site**	**Spin configuration Gd1 - Gd2**	**Relative Energy (cm^{-1})**
**High-spin**	↑ - ↑	0
**BS1**	↓ - ↑	-66.7
**BS2**	↑ - ↓	-66.7
**2-Gd**		
**State/Site**	**Spin configuration Gd1 - Gd2 - radical**	**Relative Energy (cm^{-1})**
**High-spin**	↑ - ↑ - ↑	0
**BS1**	↓ - ↑ - ↑	-211.8
**BS2**	↑ - ↓ - ↑	-211.8
**BS2**	↑ - ↑ - ↓	-216.8

Table S15. Cole-Davidson fits of AC data for **2-Dy**.

**T (K)**	**τ_{AC} (s)**	**β**	**χ_T (cm^3/mol)**	**χ_S (cm^3/mol)**
4.0	1.355205	0.298169	9.8704	0.0558
4.4	0.365812	0.303386	9.0304	0.0213
4.8	0.103848	0.325137	8.2100	0.0000
5.2	0.034548	0.342858	7.5950	0.0000
5.6	0.013043	0.364283	7.0619	0.0000
6.0	0.005519	0.386514	6.5964	0.0000
6.4	0.002545	0.413833	6.1919	0.0000
6.8	0.001287	0.441165	5.8290	0.0000
7.2	0.000666	0.492452	5.5000	0.0000
7.6	0.000390	0.522573	5.2069	0.0000

Table S16. Cole-Davidson fits of AC data for **2-Tb**.

**T (K)**	**τ_{AC} (s)**	**β**	**χ_T (cm^3/mol)**	**χ_S (cm^3/mol)**
4.0	5.702700	0.2522	7.1709	0.8137
4.5	1.254940	0.2544	6.2050	0.8117
5.0	0.280116	0.2570	5.7110	0.7192
5.5	0.048935	0.2850	5.2050	0.7029
6.0	0.011684	0.2875	4.8505	0.4806
6.5	0.003016	0.3043	4.5457	0.4000
7.0	0.000883	0.3395	4.2692	0.4000

Table S17. SA-CASSCF-SO results for Tb1 and Tb2 in **1-Tb**. CF wavefunctions calculated in the L,m_L basis in a 0.1 T field along the main magnetic axis of the ground state, and given in the J,m_J basis for components > 10%, rounded to the nearest %.

**Tb1**			**Tb2**		
**Energy (cm^{-1})**	***g***	**CF Wavefunction**	**Energy (cm^{-1})**	***g***	**CF Wavefunction**
0.0	17.94	99% $|\pm6\rangle$	0.0	17.96	99% $|\pm6\rangle$
0.0			0.0		
117.5	14.36	86% $|\pm5\rangle$	125.0	14.43	85% $|\pm5\rangle$
117.9			125.4		
221.5	10.53	39% $|\pm4\rangle$ + 35% $|\mp4\rangle$ + 11% $|\pm2\rangle$ + 10% $|\mp2\rangle$	230.8	10.59	40% $|\pm4\rangle$ + 35% $|\mp4\rangle$ + 10% $|\pm2\rangle$
229.9		44% $|\mp4\rangle$ + 39% $|\pm4\rangle$	239.2		44% $|\mp4\rangle$ + 39% $|\pm4\rangle$
310.6	-	24% $|\pm3\rangle$ + 24% $|\mp3\rangle$ + 22% $|\pm1\rangle$ + 22% $|\mp1\rangle$	318.1	-	25% $|\pm3\rangle$ + 25% $|\mp3\rangle$ + 21% $|\pm1\rangle$ + 21% $|\mp1\rangle$
361.2	-	39% $|\mp3\rangle$ + 39% $|\pm3\rangle$	368.9	-	40% $|\mp3\rangle$ + 39% $|\pm3\rangle$
399.7	-	39% $|0\rangle$ + 19% $|\pm2\rangle$ + 19% $|\mp2\rangle$ + 11% $|\pm4\rangle$ + 11% $|\mp4\rangle$	406.7	-	38% $|0\rangle$ + 20% $|\pm2\rangle$ + 20% $|\mp2\rangle$ + 10% $|\pm4\rangle$ + 10% $|\mp4\rangle$
531.3	14.52	42% $|\pm2\rangle$ + 42% $|\mp2\rangle$	540.1	14.48	42% $|\pm2\rangle$ + 42% $|\mp2\rangle$
539.3		27% $|\pm1\rangle$ + 27% $|\mp1\rangle$ + 21% $|\pm3\rangle$ + 21% $|\mp3\rangle$	548.3		28% $|\pm1\rangle$ + 28% $|\mp1\rangle$ + 20% $|\pm3\rangle$ + 20% $|\mp3\rangle$
716.7	17.84	43% $|\pm1\rangle$ + 43% $|\mp1\rangle$	734.2	17.81	43% $|\pm1\rangle$ + 43% $|\mp1\rangle$
717.4		57% $|0\rangle$ + 20% $|\pm2\rangle$ + 20% $|\mp2\rangle$	735.0		57% $|0\rangle$ + 19% $|\pm2\rangle$ + 19% $|\mp2\rangle$

Table S18. SA-CASSCF-SO results for Dy1 in **1-Dy**. CF wavefunctions calculated in the L,m_L basis in a 0.1 T field along the main magnetic axis of the ground state, and given in the J,m_J basis for components > 10%, rounded to the nearest %.

Energy (cm^{-1})	g_x	g_y	g_z	CF Wavefunction
0.0	0.01	0.00	19.51	90% $\|\pm15/2\rangle$
176.8	0.08	0.07	16.76	90% $\|\pm13/2\rangle$
355.8	5.77	2.28	11.34	56% $\|\pm11/2\rangle$ + 14% $\|\pm7/2\rangle$
398.2	11.11	5.16	1.50	25% $\|\pm11/2\rangle$ + 24% $\|\mp1/2\rangle$ + 17% $\|\mp5/2\rangle$ + 15% $\|\pm3/2\rangle$
474.1	3.47	8.57	7.15	63% $\|\pm9/2\rangle$ + 11% $\|\mp3/2\rangle$
546.3	2.97	13.32	2.37	49% $\|\pm7/2\rangle$ + 16% $\|\mp5/2\rangle$ + 14% $\|\mp9/2\rangle$ + 12% $\|\mp1/2\rangle$
656.9	0.05	16.89	0.08	24% $\|\pm5/2\rangle$ + 18% $\|\mp3/2\rangle$ + 17% $\|\mp5/2\rangle$ + 12% $\|\mp7/2\rangle$ + 10% $\|\pm7/2\rangle$
883.9	0.04	19.79	0.02	25% $\|\pm1/2\rangle$ + 22% $\|\mp1/2\rangle$ + 17% $\|\pm3/2\rangle$ + 16% $\|\mp3/2\rangle$

Table S19. SA-CASSCF-SO results for Dy2 in **1-Dy**. CF wavefunctions calculated in the L,m_L basis in a 0.1 T field along the main magnetic axis of the ground state, and given in the J,m_J basis for components > 10%, rounded to the nearest %.

Energy (cm^{-1})	g_x	g_y	g_z	CF Wavefunction
0.0	0.01	0.01	19.49	89% $\|\pm15/2\rangle$ + 10% $\|\pm11/2\rangle$
179.7	0.13	0.10	16.70	88% $\|\pm13/2\rangle$
355.0	10.33	2.84	7.78	41% $\|\pm11/2\rangle$ + 17% $\|\pm7/2\rangle$ + 14% $\|\pm3/2\rangle$ + 12% $\|\mp1/2\rangle$
399.3	7.55	5.89	3.35	37% $\|\pm11/2\rangle$ + 20% $\|\mp1/2\rangle$ + 16% $\|\mp5/2\rangle$ + 10% $\|\pm3/2\rangle$
484.5	3.87	9.32	6.28	57% $\|\pm9/2\rangle$ + 13% $\|\mp3/2\rangle$
564.0	2.13	13.83	1.68	44% $\|\pm7/2\rangle$ + 18% $\|\mp9/2\rangle$ + 14% $\|\mp5/2\rangle$ + 12% $\|\mp1/2\rangle$
683.3	0.15	17.03	0.10	27% $\|\pm7/2\rangle$ + 18% $\|\mp7/2\rangle$ + 16% $\|\mp3/2\rangle$ + 12% $\|\mp5/2\rangle$ + 10% $\|\pm5/2\rangle$
918.3	0.03	19.82	0.02	25% $\|\pm1/2\rangle$ + 22% $\|\mp1/2\rangle$ + 17% $\|\pm3/2\rangle$ + 16% $\|\pm3/2\rangle$

Table S20. CF parameters from SA-CASSCF-SO calculations Tb1 and Tb2 in **1-Tb**, in the L,m_L basis.

**Parameter**	**Tb1 (cm^{-1})**	**Tb2 (cm^{-1})**
λ_1	-297	-297
B_2^{-2}	-60	-86
B_2^{-1}	6	55
B_2^{0}	537	551
B_2^{+1}	-5	74
B_2^{+2}	578	573
B_4^{-4}	4	5
B_4^{-3}	6	56
B_4^{-2}	-14	-3
B_4^{-1}	-15	-4
B_4^{0}	21	22
B_4^{+1}	-5	3
B_4^{+2}	-6	30
B_4^{+3}	28	64
B_4^{+4}	16	21
B_6^{-6}	11	1
B_6^{-5}	-98	130
B_6^{-4}	5	-7
B_6^{-3}	-48	71
B_6^{-2}	18	-25
B_6^{-1}	-23	14
B_6^{0}	4	8
B_6^{+1}	30	33
B_6^{+2}	-395	-384
B_6^{+3}	-2	-19
B_6^{+4}	12	-35
B_6^{+5}	-18	28
B_6^{+6}	-51	-48

Table S21. CF parameters from SA-CASSCF-SO calculations Dy1 and Dy2 in **1-Dy**.

**Parameter**	**Dy1 (cm^{-1})**	**Dy2 (cm^{-1})**
λ_1	-396	-396
B_2^{-2}	34	60
B_2^{-1}	-90	87
B_2^{0}	583	593
B_2^{+1}	64	-104
B_2^{+2}	670	740
B_4^{-4}	4	18
B_4^{-3}	21	-1
B_4^{-2}	-4	-8
B_4^{-1}	-14	14
B_4^{0}	21	23
B_4^{+1}	5	-13
B_4^{+2}	9	20
B_4^{+3}	-17	80
B_4^{+4}	-3	4
B_6^{-6}	-5	7
B_6^{-5}	85	-115
B_6^{-4}	-8	22
B_6^{-3}	63	-85
B_6^{-2}	-42	27
B_6^{-1}	10	-16
B_6^{0}	6	7
B_6^{+1}	4	36
B_6^{+2}	-378	-381
B_6^{+3}	-18	-5
B_6^{+4}	-17	-32
B_6^{+5}	-48	30
B_6^{+6}	-58	-51

Table S22. Projected model Hamiltonian parameters from SA-CASSCF-SO calculations for the Tb^{III}-radical pairs in **2-Tb**. Calculations performed for inequivalent Tb1 and Tb2 sites. Exchange parameters with average magnitude > 5 cm^{-1} shown.

Parameter	Tb1 (cm^{-1})	Tb2 (cm^{-1})	Parameter	Tb1 (cm^{-1})	Tb2 (cm^{-1})	Effective Operator
λ_1	−297	−297	$J^{RSL}_{+1,1,+1,4,+4}$	-39	-37	$\hat{R}_x\hat{S}_x\hat{O}_4^{+4}$
B_2^{-2}	0	0	$J^{RSL}_{-1,1,-1,4,+4}$	-39	-37	$\hat{R}_y\hat{S}_y\hat{O}_4^{+4}$
B_2^{-1}	-40	524	$J^{RSL}_{0,1,0,4,+4}$	-39	-37	$\hat{R}_z\hat{S}_z\hat{O}_4^{+4}$
B_2^{0}	390	310	$J^{RSL}_{+1,1,+1,4,-3}$	6	-15	$\hat{R}_x\hat{S}_x\hat{O}_4^{-3}$
B_2^{+1}	0	0	$J^{RSL}_{-1,1,-1,+1,4,-3}$	6	-15	$\hat{R}_y\hat{S}_y\hat{O}_4^{-3}$
B_2^{+2}	779	735	$J^{RSL}_{0,1,0,4,-3}$	6	-15	$\hat{R}_z\hat{S}_z\hat{O}_4^{-3}$
B_4^{-4}	0	0	$J^{RSL}_{+1,1,+1,6,+4}$	7	7	$\hat{R}_x\hat{S}_x\hat{O}_6^{+4}$
B_4^{-3}	31	82	$J^{RSL}_{-1,1,-1,6,+4}$	7	7	$\hat{R}_y\hat{S}_y\hat{O}_6^{+4}$
B_4^{-2}	0	0	$J^{RSL}_{0,1,0,6,+4}$	7	7	$\hat{R}_z\hat{S}_z\hat{O}_6^{+4}$
B_4^{-1}	11	24	$J^{RSL}_{+1,1,+1,4,0}$	-7	-7	$\hat{R}_x\hat{S}_x\hat{O}_4^{0}$
B_4^{0}	18	11	$J^{RSL}_{-1,1,-1,4,0}$	-7	-7	$\hat{R}_y\hat{S}_y\hat{O}_4^{0}$
B_4^{+1}	0	0	$J^{RSL}_{0,1,0,4,0}$	-7	-7	$\hat{R}_z\hat{S}_z\hat{O}_4^{0}$
B_4^{+2}	62	50	$J^{RSL}_{0,1,-1,4,-1}$	6	5	$\hat{R}_z\hat{S}_y\hat{O}_4^{-1}$
B_4^{+3}	0	0	$J^{RSL}_{-1,1,0,4,-1}$	-6	-5	$\hat{R}_y\hat{S}_z\hat{O}_4^{-1}$
B_4^{+4}	0	1	$J^{RS}_{+1,1,+1}$	-2	8	$\hat{R}_x\hat{S}_x$
B_6^{-6}	0	0	$J^{RS}_{-1,1,-1}$	-2	8	$\hat{R}_y\hat{S}_y$
B_6^{-5}	18	-62	$J^{RS}_{0,1,0}$	-2	8	$\hat{R}_z\hat{S}_z$
B_6^{-4}	0	0				
B_6^{-3}	13	-280				
B_6^{-2}	0	0				
B_6^{-1}	18	-52				
B_6^{0}	31	27				
B_6^{+1}	0	0				
B_6^{+2}	-440	-376				
B_6^{+3}	0	0				
B_6^{+4}	-115	-68				

B_6^{+5}	0	0				
B_6^{+6}	-8	-3				

Table S23. Projected model Hamiltonian parameters from SA-CASSCF-CASPT2-SO calculations for the Tb^{III}-radical pairs in **2-Tb**. Calculations performed for inequivalent Tb1 and Tb2 sites. Exchange parameters with average magnitude > 20 cm^{-1} shown.

Parameter	Tb1 (cm^{-1})	Tb2 (cm^{-1})	Parameter	Tb1 (cm^{-1})	Tb2 (cm^{-1})	Effective Operator
λ_1	-297	-297	$J^{RSL}_{+1,1,+1,4,+4}$	-137	-125	$\hat{R}_x\hat{S}_x\hat{O}_4^{+4}$
B_2^{-2}	0	0	$J^{RSL}_{-1,1,-1,4,+4}$	-137	-125	$\hat{R}_y\hat{S}_y\hat{O}_4^{+4}$
B_2^{-1}	-51	355	$J^{RSL}_{0,1,0,4,+4}$	-137	-125	$\hat{R}_z\hat{S}_z\hat{O}_4^{+4}$
B_2^{0}	600	174	$J^{RS}_{+1,1,+1}$	-145	-79	$\hat{R}_x\hat{S}_x$
B_2^{+1}	1	0	$J^{RS}_{-1,1,-1}$	-145	-79	$\hat{R}_y\hat{S}_y$
B_2^{+2}	1388	445	$J^{RS}_{0,1,0}$	-145	-79	$\hat{R}_z\hat{S}_z$
B_4^{-4}	0	0	$J^{RSL}_{+1,1,+1,2,-1}$	-6	-170	$\hat{R}_x\hat{S}_x\hat{O}_2^{-1}$
B_4^{-3}	14	-73	$J^{RSL}_{-1,1,-1,2,-1}$	-6	-170	$\hat{R}_y\hat{S}_y\hat{O}_2^{-1}$
B_4^{-2}	0	0	$J^{RSL}_{0,1,0,2,-1}$	-6	-170	$\hat{R}_z\hat{S}_z\hat{O}_2^{-1}$
B_4^{-1}	-2	-40	$J^{RSL}_{+1,1,+1,4,-3}$	-5	120	$\hat{R}_x\hat{S}_x\hat{O}_4^{-3}$
B_4^{0}	59	1	$J^{RSL}_{-1,1,-1,4,-3}$	-5	120	$\hat{R}_y\hat{S}_y\hat{O}_4^{-3}$
B_4^{+1}	0	0	$J^{RSL}_{0,1,0,4,-3}$	-5	120	$\hat{R}_z\hat{S}_z\hat{O}_4^{-3}$
B_4^{+2}	36	-20	$J^{RSL}_{+1,1,+1,2,+2}$	-23	-86	$\hat{R}_x\hat{S}_x\hat{O}_2^{+2}$
B_4^{+3}	0	0	$J^{RSL}_{-1,1,-1,2,+2}$	-23	-86	$\hat{R}_y\hat{S}_y\hat{O}_2^{+2}$
B_4^{+4}	140	-190	$J^{RSL}_{0,1,0,2,+2}$	-23	-86	$\hat{R}_z\hat{S}_z\hat{O}_2^{+2}$
B_6^{-6}	0	0	$J^{RSL}_{+1,1,+1,6,+4}$	23	46	$\hat{R}_x\hat{S}_x\hat{O}_6^{+4}$
B_6^{-5}	11	-22	$J^{RSL}_{-1,1,-1,6,+4}$	23	46	$\hat{R}_y\hat{S}_y\hat{O}_6^{+4}$
B_6^{-4}	0	0	$J^{RSL}_{0,1,0,6,+4}$	23	46	$\hat{R}_z\hat{S}_z\hat{O}_6^{+4}$
B_6^{-3}	-15	-422	$J^{RSL}_{+1,1,+1,6,-5}$	-4	65	$\hat{R}_x\hat{S}_x\hat{O}_6^{-5}$
B_6^{-2}	0	0	$J^{RSL}_{-1,1,-1,6,-5}$	-4	65	$\hat{R}_y\hat{S}_y\hat{O}_6^{-5}$
B_6^{-1}	35	-72	$J^{RSL}_{0,1,0,6,-5}$	-4	65	$\hat{R}_z\hat{S}_z\hat{O}_6^{-5}$
B_6^{0}	29	45	$J^{RSL}_{+1,1,+1,4,0}$	-27	-29	$\hat{R}_x\hat{S}_x\hat{O}_4^{0}$

B_6^{+1}	0	0	$J_{-1,1,-1,4,0}^{RSL}$	-27	-29	$\hat{R}_y\hat{S}_y\hat{O}_4^0$
B_6^{+2}	-580	-592	$J_{0,1,0,4,0}^{RSL}$	-27	-29	$\hat{R}_z\hat{S}_z\hat{O}_4^0$
B_6^{+3}	-1	0	$J_{+1,1,+1,6,-3}^{RSL}$	-1	-38	$\hat{R}_x\hat{S}_x\hat{O}_6^{-3}$
B_6^{+4}	-15	-68	$J_{-1,1,-1,6,-3}^{RSL}$	-1	-38	$\hat{R}_y\hat{S}_y\hat{O}_6^{-3}$
B_6^{+5}	2	0	$J_{0,1,0,6,-3}^{RSL}$	-1	-38	$\hat{R}_z\hat{S}_z\hat{O}_6^{-3}$
B_6^{+6}	102	12				

Table S24. CF energies and wavefunctions for Tb1 in **2-Tb** based on projected parameters from SA-CASSCF-SO calculations on a Tb^{III}-radical pair. CF wavefunctions calculated in the *L,m* basis in a 0.1 T field along the main magnetic axis of the ground state, and given in the J,m_J basis for components > 10%, rounded to the nearest %.

**Energy (cm^{-1})**	***g***	**CF Wavefunction**
0.0	17.65	86% $\|-6\rangle$
0.6		87% $\|+6\rangle$
52.1	-	28% $\|-5\rangle$ + 25% $\|+5\rangle$ + 15% $\|-3\rangle$ + 14% $\|+3\rangle$
68.6	-	39% $\|+5\rangle$ + 37% $\|-5\rangle$ + 11% $\|+3\rangle$ + 10% $\|-3\rangle$
80.6	-	20% $\|-2\rangle$ + 20% $\|+2\rangle$ + 19% $\|-4\rangle$ + 18% $\|+4\rangle$ + 16% $\|0\rangle$
129.3	-	35% $\|-4\rangle$ + 35% $\|+4\rangle$ + 13% $\|+2\rangle$ + 12% $\|-2\rangle$
153.8	-	22% $\|-1\rangle$ + 22% $\|+1\rangle$ + 20% $\|-5\rangle$ + 20% $\|+5\rangle$
253.2	-	30% $\|-3\rangle$ + 30% $\|+3\rangle$ + 26% $\|-4\rangle$ + 11% $\|+5\rangle$ + 11% $\|+5\rangle$
264.9	-	32% $\|0\rangle$ + 26% $\|+4\rangle$ + 12% $\|-4\rangle$
437.2	15.05	37% $\|-2\rangle$ + 37% $\|+2\rangle$ + 12% $\|+4\rangle$
439.4		28% $\|-3\rangle$ + 28% $\|+3\rangle$ + 19% $\|-1\rangle$ + 19% $\|+1\rangle$
646.0	17.94	43% $\|-1\rangle$ + 43% $\|+1\rangle$
646.5		51% $\|0\rangle$ + 22% $\|-2\rangle$ + 22% $\|+2\rangle$

Table S25. CF energies and wavefunctions for Tb1 in **2-Tb** based on projected parameters from SA-CASSCF-CASPT2-SO calculations on a Tb^{III}-radical pair. CF wavefunctions calculated in the L,m_L basis in a 0.1 T field along the main magnetic axis of the ground state, and given in the J,m_J basis for components > 10%, rounded to the nearest %.

**Energy (cm^{-1})**	***g***	**CF Wavefunction**
0.0	16.22	41% $\|-6\rangle$ + 32% $\|+6\rangle$
6.7		47% $\|+6\rangle$ + 38% $\|-6\rangle$
30.3	-	26% $\|-5\rangle$ + 25% $\|+5\rangle$ + 16% $\|-3\rangle$ + 16% $\|+3\rangle$

58.9	-	37% $\|+5\rangle$ + 36% $\|-5\rangle$ + 12% $\|+3\rangle$ + 12% $\|-3\rangle$
93.0	-	18% $\|-2\rangle$ + 17% $\|+2\rangle$ + 15% $\|0\rangle$ + 12% $\|+6\rangle$ + 12% $\|-6\rangle$ + 12% $\|-4\rangle$ + 12% $\|+4\rangle$
164.6	-	30% $\|+4\rangle$ + 30% $\|-4\rangle$ + 12% $\|+2\rangle$ + 12% $\|-2\rangle$
201.3	-	21% $\|+5\rangle$ + 21% $\|-5\rangle$ + 22% $\|-1\rangle$ + 22% $\|+1\rangle$
353.6	-	27% $\|-3\rangle$ + 27% $\|+3\rangle$ + 12% $\|+5\rangle$ + 12% $\|-5\rangle$
366.2	-	25% $\|+4\rangle$ + 25% $\|-4\rangle$ + 32% $\|0\rangle$
643.9	15.24	35% $\|-2\rangle$ + 35% $\|+2\rangle$ + 12% $\|+4\rangle$ + 12% $\|-4\rangle$
647.5		28% $\|+3\rangle$ + 28% $\|-3\rangle$ + 17% $\|-1\rangle$ + 17% $\|+1\rangle$
1076	17.92	40% $\|+1\rangle$ + 40% $\|-1\rangle$
1077		48% $\|0\rangle$ + 23% $\|+2\rangle$ + 23% $\|-2\rangle$

Table S26. Projected model Hamiltonian parameters from SA-CASSCF-SO calculations for the Dy^{III}-radical pair in **2-Dy**. Calculations performed for symmetry-equivalent Dy1 sites (inversion related). Exchange parameters with magnitude > 10 cm^{-1} shown.

Parameter	Value (cm^{-1})	Parameter	Value (cm^{-1})	Effective Operator
λ_1	-395	$J^{RSL}_{+1,1,+1,4,+4}$	42	$\hat{R}_x\hat{S}_x\hat{O}_4^{+4}$
B_2^{-2}	0	$J^{RSL}_{-1,1,-1,4,+4}$	42	$\hat{R}_y\hat{S}_y\hat{O}_4^{+4}$
B_2^{-1}	247	$J^{RSL}_{0,1,0,4,+4}$	42	$\hat{R}_z\hat{S}_z\hat{O}_4^{+4}$
B_2^{0}	310	$J^{RSL}_{+1,1,+1,6,+4}$	-31	$\hat{R}_x\hat{S}_x\hat{O}_6^{+4}$
B_2^{+1}	0	$J^{RSL}_{-1,1,-1,6,+4}$	-31	$\hat{R}_y\hat{S}_y\hat{O}_6^{+4}$
B_2^{+2}	1033	$J^{RSL}_{0,1,0,6,+4}$	-31	$\hat{R}_z\hat{S}_z\hat{O}_6^{+4}$
B_4^{-4}	0	$J^{RSL}_{-1,1,0,6,-5}$	-21	$\hat{R}_y\hat{S}_z\hat{O}_6^{-5}$
B_4^{-3}	191	$J^{RSL}_{0,1,-1,6,-5}$	21	$\hat{R}_z\hat{S}_y\hat{O}_6^{-5}$
B_4^{-2}	0	$J^{RS}_{+1,1,+1}$	19	$\hat{R}_x\hat{S}_x$
B_4^{-1}	24	$J^{RS}_{-1,1,-1}$	19	$\hat{R}_y\hat{S}_y$
B_4^{0}	8	$J^{RS}_{0,1,0}$	19	$\hat{R}_z\hat{S}_z$
B_4^{+1}	0	$J^{RSL}_{0,2,-1,5,-2}$	-15	$\hat{R}_z\hat{O}_2^{-1}\hat{O}_5^{-2}$
B_4^{+2}	90	$J^{RSL}_{+1,1,+1,4,-3}$	13	$\hat{R}_x\hat{S}_x\hat{O}_4^{-3}$
B_4^{+3}	0	$J^{RSL}_{-1,1,-1,+1,4,-3}$	13	$\hat{R}_y\hat{S}_y\hat{O}_4^{-3}$
B_4^{+4}	15	$J^{RSL}_{0,1,0,4,-3}$	13	$\hat{R}_z\hat{S}_z\hat{O}_4^{-3}$

B_6^{-6}	0	$J^{RSL}_{+1,1,+1,6,-5}$	-12	$\hat{R}_x\hat{S}_x\hat{O}_6^{-5}$
B_6^{-5}	-325	$J^{RSL}_{-1,1,-1,6,-5}$	-12	$\hat{R}_y\hat{S}_y\hat{O}_6^{-5}$
B_6^{-4}	0	$J^{RSL}_{0,1,0,6,-5}$	-12	$\hat{R}_z\hat{S}_z\hat{O}_6^{-5}$
B_6^{-3}	-517	$J^{RSL}_{0,2,+1,5,-2}$	12	$\hat{R}_z\hat{O}_2^{+1}\hat{O}_5^{-2}$
B_6^{-2}	0	$J^{RSL}_{+1,1,+1,4,+2}$	-12	$\hat{R}_x\hat{S}_x\hat{O}_4^{+2}$
B_6^{-1}	12	$J^{RSL}_{-1,1,-1,4,+2}$	-12	$\hat{R}_y\hat{S}_y\hat{O}_4^{+2}$
B_6^{0}	39	$J^{RSL}_{0,1,0,4,+2}$	-12	$\hat{R}_z\hat{S}_z\hat{O}_4^{+2}$
B_6^{+1}	0	$J^{RSL}_{0,2,+1,5,+5}$	10	$\hat{R}_z\hat{O}_2^{+1}\hat{O}_5^{+5}$
B_6^{+2}	-375	$J^{RSL}_{0,2,-1,5,-5}$	10	$\hat{R}_z\hat{O}_2^{-1}\hat{O}_5^{-5}$
B_6^{+3}	0	$J^{RSL}_{+1,2,+1,5,+2}$	-10	$\hat{R}_x\hat{O}_2^{+1}\hat{O}_5^{+2}$
B_6^{+4}	-16	$J^{RSL}_{-1,2,-1,5,+2}$	-10	$\hat{R}_y\hat{O}_2^{-1}\hat{O}_5^{+2}$
B_6^{+5}	0			
B_6^{+6}	-27			

Table S27. Projected model Hamiltonian parameters from SA-CASSCF-CASPT2-SO calculations for the Dy^{III}-radical pair in **2-Dy**. Calculations performed for symmetry-equivalent Dy1 sites (inversion related). Exchange parameters with magnitude > 20 cm^{-1} shown.

Parameter	Value (cm^{-1})	Parameter	Value (cm^{-1})	Effective Operator
λ_1	-395	$J^{RS}_{+1,1,+1}$	-121	$\hat{R}_x\hat{S}_x$
B_2^{-2}	0	$J^{RS}_{-1,1,-1}$	-121	$\hat{R}_y\hat{S}_y$
B_2^{-1}	169	$J^{RS}_{0,1,0}$	-121	$\hat{R}_z\hat{S}_z$
B_2^{0}	171	$J^{RSL}_{+1,1,+1,8,-5}$	-102	$\hat{R}_x\hat{S}_x\hat{O}_8^{-5}$
B_2^{+1}	0	$J^{RSL}_{-1,1,-1,8,-5}$	-102	$\hat{R}_y\hat{S}_y\hat{O}_8^{-5}$
B_2^{+2}	819	$J^{RSL}_{0,1,0,8,-5}$	-102	$\hat{R}_z\hat{S}_z\hat{O}_8^{-5}$
B_4^{-4}	0	$J^{RSL}_{+1,1,+1,4,+4}$	99	$\hat{R}_x\hat{S}_x\hat{O}_4^{+4}$
B_4^{-3}	265	$J^{RSL}_{-1,1,-1,4,+4}$	99	$\hat{R}_y\hat{S}_y\hat{O}_4^{+4}$

B_4^{-2}	0	$J_{0,1,0,4,+4}^{RSL}$	99	$\hat{R}_z\hat{S}_z\hat{O}_4^{+4}$
B_4^{-1}	-53	$J_{+1,1,+1,6,+4}^{RSL}$	-85	$\hat{R}_x\hat{S}_x\hat{O}_6^{+4}$
B_4^{0}	-3	$J_{-1,1,-1,6,+4}^{RSL}$	-85	$\hat{R}_y\hat{S}_y\hat{O}_6^{+4}$
B_4^{+1}	0	$J_{0,1,0,6,+4}^{RSL}$	-85	$\hat{R}_z\hat{S}_z\hat{O}_6^{+4}$
B_4^{+2}	170	$J_{+1,1,+1,8,+8}^{RSL}$	-63	$\hat{R}_x\hat{S}_x\hat{O}_8^{+8}$
B_4^{+3}	0	$J_{-1,1,-1,8,+8}^{RSL}$	-63	$\hat{R}_y\hat{S}_y\hat{O}_8^{+8}$
B_4^{+4}	-22	$J_{0,1,0,8,+8}^{RSL}$	-63	$\hat{R}_z\hat{S}_z\hat{O}_8^{+8}$
B_6^{-6}	0	$J_{+1,1,+1,8,-7}^{RSL}$	-61	$\hat{R}_x\hat{S}_x\hat{O}_8^{-7}$
B_6^{-5}	-553	$J_{-1,1,-1,8,-7}^{RSL}$	-61	$\hat{R}_y\hat{S}_y\hat{O}_8^{-7}$
B_6^{-4}	0	$J_{0,1,0,8,-7}^{RSL}$	-61	$\hat{R}_z\hat{S}_z\hat{O}_8^{-7}$
B_6^{-3}	-596	$J_{+1,1,+1,6,-5}^{RSL}$	-59	$\hat{R}_x\hat{S}_x\hat{O}_6^{-5}$
B_6^{-2}	0	$J_{-1,1,-1,6,-5}^{RSL}$	-59	$\hat{R}_y\hat{S}_y\hat{O}_6^{-5}$
B_6^{-1}	61	$J_{0,1,0,6,-5}^{RSL}$	-59	$\hat{R}_z\hat{S}_z\hat{O}_6^{-5}$
B_6^{0}	44	$J_{+1,1,+1,4,+2}^{RSL}$	-32	$\hat{R}_x\hat{S}_x\hat{O}_4^{+2}$
B_6^{+1}	0	$J_{-1,1,-1,4,+2}^{RSL}$	-32	$\hat{R}_y\hat{S}_y\hat{O}_4^{+2}$
B_6^{+2}	-399	$J_{0,1,0,4,+2}^{RSL}$	-32	$\hat{R}_z\hat{S}_z\hat{O}_4^{+2}$
B_6^{+3}	0	$J_{+1,1,+1,8,+2}^{RSL}$	29	$\hat{R}_x\hat{S}_x\hat{O}_8^{+2}$
B_6^{+4}	-58	$J_{-1,1,-1,8,+2}^{RSL}$	29	$\hat{R}_y\hat{S}_y\hat{O}_8^{+2}$
B_6^{+5}	0	$J_{0,1,0,8,+2}^{RSL}$	29	$\hat{R}_z\hat{S}_z\hat{O}_8^{+2}$
B_6^{+6}	52	$J_{+1,1,+1,6,-3}^{RSL}$	-21	$\hat{R}_x\hat{S}_x\hat{O}_6^{-3}$
		$J_{-1,1,-1,6,-3}^{RSL}$	-21	$\hat{R}_y\hat{S}_y\hat{O}_6^{-3}$
		$J_{0,1,0,6,-3}^{RSL}$	-21	$\hat{R}_z\hat{S}_z\hat{O}_6^{-3}$
		$J_{+1,1,+1,4,0}^{RSL}$	21	$\hat{R}_x\hat{S}_x\hat{O}_4^{0}$
		$J_{-1,1,-1,4,0}^{RSL}$	21	$\hat{R}_y\hat{S}_y\hat{O}_4^{0}$
		$J_{0,1,0,4,0}^{RSL}$	21	$\hat{R}_z\hat{S}_z\hat{O}_4^{0}$
		$J_{0,1,-1,6,-5}^{RSL}$	21	$\hat{R}_z\hat{S}_y\hat{O}_6^{-5}$
		$J_{-1,1,0,6,-5}^{RSL}$	-21	$\hat{R}_y\hat{S}_z\hat{O}_6^{-5}$

Table S28. SA-CASSCF-SO results for the Dy^{III} sites in **2-Dy**. CF wavefunctions calculated in the *L,m_L* basis in a 0.1 T field along the main magnetic axis of the ground state, and given in the *J,m_J* basis for components > 10%, rounded to the nearest %.

Energy (cm^{-1})	g_x	g_y	g_z	CF Wavefunction
0.0	7.31	1.09	13.43	41% $\|\pm15/2\rangle$ + 21% $\|\pm11/2\rangle$
36.6	0.22	3.08	5.83	30% $\|\pm13/2\rangle$ + 11% $\|\mp15/2\rangle$
91.6	11.13	3.01	5.36	34% $\|\pm13/2\rangle$ + 16% $\|\mp1/2\rangle$
262.3	10.37	4.66	6.52	23% $\|\pm7/2\rangle$ + 20% $\|\pm15/2\rangle$ + 16% $\|\mp5/2\rangle$ + 11% $\|\pm11/2\rangle$
342.1	1.98	11.28	2.74	29% $\|\pm11/2\rangle$ + 16% $\|\mp9/2\rangle$ + 12% $\|\pm15/2\rangle$ + 10% $\|\pm3/2\rangle$ + 10% $\|\mp3/2\rangle$
439.5	0.04	14.30	0.88	34% $\|\pm9/2\rangle$ + 18% $\|\mp7/2\rangle$ + 15% $\|\mp11/2\rangle$ + 12% $\|\pm1/2\rangle$
556.0	0.49	17.41	0.55	23% $\|\pm7/2\rangle$ + 16% $\|\pm5/2\rangle$ + 13% $\|\pm3/2\rangle$ + 13% $\|\mp5/2\rangle$
772.4	0.01	19.96	0.01	19% $\|\pm1/2\rangle$ + 18% $\|\pm3/2\rangle$ + 17% $\|\mp1/2\rangle$ + 15% $\|\pm5/2\rangle$ + 12% $\|\mp3/2\rangle$

Table S29. SA-CASSCF-CASPT2-SO results for the Dy^{III} sites in **2-Dy**. CF wavefunctions calculated in the *L,m_L* basis in a 0.1 T field along the main magnetic axis of the ground state, and given in the *J,m_J* basis for components > 10%, rounded to the nearest %.

Energy (cm^{-1})	g_x	g_y	g_z	CF Wavefunction
0.0	12.30	1.00	8.52	24% $\|\pm15/2\rangle$ + 24% $\|\pm11/2\rangle$ + 12% $\|\pm1/2\rangle$
23.5	6.90	2.39	3.71	33% $\|\pm13/2\rangle$ + 14% $\|\mp15/2\rangle$ + 11% $\|\mp11/2\rangle$
66.1	13.45	1.87	3.62	30% $\|\pm13/2\rangle$ + 21% $\|\mp1/2\rangle$ + 11% $\|\pm3/2\rangle$
224.8	13.94	2.84	1.43	27% $\|\pm5/2\rangle$ + 24% $\|\mp7/2\rangle$ + 17% $\|\mp3/2\rangle$ + 11% $\|\mp15/2\rangle$
301.9	6.14	9.98	4.84	25% $\|\pm11/2\rangle$ + 21% $\|\pm15/2\rangle$ + 10% $\|\mp9/2\rangle$
370.7	1.09	13.14	0.42	18% $\|\pm9/2\rangle$ + 15% $\|\mp9/2\rangle$ + 13% $\|\pm15/2\rangle$
434.7	0.79	17.52	1.07	26% $\|\pm7/2\rangle$ + 18% $\|\pm5/2\rangle$
595.0	0.03	19.89	0.01	18% $\|\pm1/2\rangle$ + 18% $\|\pm3/2\rangle$ + 16% $\|\pm5/2\rangle$ + 16% $\|\mp1/2\rangle$ + 11% $\|\pm7/2\rangle$ + 11% $\|\mp3/2\rangle$

References

1. Evans, W. J.; Kozimor, S. A.; Ziller, J. W.; Kaltsoyannis, N. *J. Am. Chem. Soc.* 2004, 126, 14533–14547.
2. Demir, S.; Zadrozny, J. M.; Nippe, M.; Long, J. R. *J. Am. Chem. Soc.* 2012, 134, 18546-18549.
3. Zhang, P; Benner, F; Chilton, N. F.; Demir, S. *Chem* 2022, 8, 717–730
4. COSMO V1.61, *Software for the CCD Detector Systems for Determining Data Collection Parameters*. Bruker Analytical X-ray Systems, Madison, WI (2009).
5. APEX2 V2010.11-3. *Software for the CCD Detector System*. Bruker Analytical X-ray Systems, Madison, WI (2010).
6. SAINT V 7.68A *Software for the Integration of CCD Detector System*. Bruker Analytical X-ray Systems, Madison, WI (2010).
7. SADABS V2.008/2 Program for absorption corrections using Bruker-AXS CCD based on the method of Robert Blessing; Blessing, R.H. *Acta Cryst.* A51, 1995, 33-38.
8. CrysAlisPro Software System, Rigaku Corporation, Oxford, 2020.
9. SCALE3 ABSPACK Empirical Absorption Correction, CrysAlisPro Software Package, Rigaku Corporation, Oxford, 2020.
10. Dolomanov, O. V.; Bourhis, L. J.; Gildea, R. J.; Howard, J. A. K.; Puschmann, H. *J. Appl. Crystallogr.*, 2009, 42, 339–341.
11. Sheldrick, G. M. *Acta Crystallogr., Sect. A: Cryst. Phys., Diffr., Theor. Gen. Crystallogr.*, 2015, 71, 3–8.
12. Sheldrick, G. M. *Acta Crystallogr., Sect. C: Struct. Chem.*, 2015, 71, 3–8.
13. E. A. Boudreaux and L. N. Mulay, *Theory and Applications of Molecular Paramagnetism*, John Wiley & Sons, New York, 1976.
14. Frisch, M. J.; Trucks, G. W.; Schlegel, H. B.; Scuseria, G. E.; Robb, M. A.; Cheeseman, J. R.; Scalmani, G.; Barone, V.; Mennucci, B.; Petersson, G. A.; Nakatsuji, H.; Caricato, M.; Li, X.; Hratchian, H. P.; Izmaylov, A. F.; Bloino, J.; Zheng, G.; Sonnenberg, J. L.; Hada, M.; Ehara, M.; Toyota, K.; Fukuda, R.; Hasegawa, J.; Ishida, M.; Nakajima, T.; Honda, Y.; Kitao, O.; Nakai, H.; Vreven, T.; Montgomery, J. A., Jr.; Peralta, J. E.; Ogliaro, F.; Bearpark, M.; Heyd, J. J.; Brothers, E.; Kudin, K. N.; Staroverov, V. N.; Kobayashi, R.; Normand, J.; Raghavachari, K.; Rendell, A.; Burant, J. C.; Iyengar, S. S.; Tomasi, J.; Cossi, M.; Rega, N.; Millam, J. M.; Klene, M.; Knox, J. E.; Cross, J. B.; Bakken, V.; Adamo, C.; Jaramillo, J.; Gomperts, R.; Stratmann, R. E.; Yazyev, O.; Austin, A. J.; Cammi, R.; Pomelli, C.; Ochterski, J. W.; Martin, R. L.; Morokuma, K.; Zakrzewski, V. G.; Voth, G. A.; Salvador, P.; Dannenberg, J. J.; Dapprich, S.; Daniels, A. D.; Farkas, Ö.; Foresman, J. B.; Ortiz, J. V.; Cioslowski, J.; Fox, D. J. Gaussian 09, Revision D.01, Wallingford CT 2013.
15. Becke, A.D. *J. Chem. Phys.* **1993**, *98*, 5648-5652.
16. Lee, C.; Yang, W.; Parr, R.G. *Phys. Rev. B*, **1998**, *37*, 785-789.
17. Stephens, P.J.; Devlin, F.J.; Chabalowski, C.F.; Frisch, M.J. *J. Phys. Chem*. **1994**, *98*, 11623-11627.
18. Ditchfield, R; Hehre, W.J; Pople, J. A. *J. Chem. Phys*. **1971**, *54*, 724–728.
19. Chan, W. T. *Chem. Phys. Lett*. **1999**, *315*, 257–265.
20. Valdés, Á.; Prosmiti, R.; Villarreal, P.; Delgado-Barrio, G. *Mol. Phys.* **2004**, *102*, 2277–

2283.
21. Lei, M.; Wang, N.; Zhu, L.H.; Tang, H.Q. *Chemosphere* **2016**, *150*, 536–544.
22. Cundari, T. R.; Stevens, W. J. *J. Chem. Phys.*, **1993**, *98*, 5555–5565.
23. Noodleman, L.; Case, D. A.; Aizman, A. *J. Am. Chem. Soc.* **1988**, *110*, 1001–1005
24. Fdez. Galván, I.; Vacher, M.; Alavi, A.; Angeli, C.; Aquilante, F.; Autschbach, J.; Bao, J. J.; Bokarev, S. I.; Bogdanov, N. A.; Carlson, R. K.; Chibotaru, L. F.; Creutzberg, J.; Dattani, N.; Delcey, M. G.; Dong, S. S.; Dreuw, A.; Freitag, L.; Frutos, L. M.; Gagliardi, L.; Gendron, F.; Giussani, A.; González, L.; Grell, G.; Guo, M.; Hoyer, C. E.; Johansson, M.; Keller, S.; Knecht, S.; Kovačević, G.; Källman, E.; Li Manni, G.; Lundberg, M.; Ma, Y.; Mai, S.; Malhado, J. P.; Malmqvist, P. Å.; Marquetand, P.; Mewes, S. A.; Norell, J.; Olivucci, M.; Oppel, M.; Phung, Q. M.; Pierloot, K.; Plasser, F.; Reiher, M.; Sand, A. M.; Schapiro, I.; Sharma, P.; Stein, C. J.; Sørensen, L. K.; Truhlar, D. G.; Ugandi, M.; Ungur, L.; Valentini, A.; Vancoillie, S.; Veryazov, V.; Weser, O.; Wesołowski, T. A.; Widmark, P.-O.; Wouters, S.; Zech, A.; Zobel, J. P.; Lindh, R. *J. Chem. Theory Comput.* **2019**, *15*, 5925–5964.
25. Reiher, M. *Theor Chem Acc*, **2006**, *116*, 241–252.
26. Roos, B. O.; Lindh, R.; Malmqvist, P.A.; Veryazov, V.; Widmark, P.O. *J. Phys. Chem. A*, **2005**, *109*, 6575–6579.
27. Roos, B. O.; Lindh, R.; Malmqvist, P.A.; Veryazov, V.; Widmark, P.O. *J. Phys. Chem. A*, **2004**, *108*, 2851–2858.
28. Manni, G. L.; Carlson, R. K.; Luo, S.; Ma, D.; Olsen, J.; Truhlar, D. G.; Gagliardi, L. *J. Chem. Theory Comput.* **2014**, *10*, 3669–3680.
29. Finley, J.; Malmqvist, P.-Å.; Roos, B. O.; Serrano-Andrés, L. *Chem. Phys. Lett.* **1998**, *288*, 299–306.
30. Aquilante, F.; Pedersen, T. B.; Merás, A. S. d.; Koch, H. *J. Chem. Phys.* **2006**, 125, 174101.
31. Erratum for the Report "Ultrahard magnetism from mixed-valence dilanthanide complexes with metal-metal bonding," by C. A. Gould et al. *Science* **2022**, *378*, eadf5804.
32. Gould, C. A.; McClain, K. R.; Reta, D.; Kragskow, J. G. C.; Marchiori, D. A.; Lachman, E.; Choi, E.-S.; Analytis, J. G.; Britt, R. D.; Chilton, N. F.; Harvey, B. G.; Long, J. R. *Science* **2022**, *375*, 198-202.
33. Iwahara, N.; Chibotaru, L. F. *Phys. Rev. B* **2015**, *91*, 174438.
34. Chilton, N. F.; Anderson, R. P.; Turner, L. D.; Soncini, A.; Murray, K. S. *J. Comput. Chem*. **2013**, *34*, 1164-1175.
35. Zorn, R. *J. Chem. Phys.* **2002**, *116*, 3204–3209.
